# An exploration of differences in the scaling of life history traits with body mass within reptiles and between amniotes

**DOI:** 10.1002/ece3.4069

**Published:** 2018-05-02

**Authors:** Konstantin Hallmann, Eva Maria Griebeler

**Affiliations:** ^1^ Institute of Organismic and Molecular Evolution – Evolutionary Ecology Johannes Gutenberg‐University Mainz Mainz Rhineland‐Palatinate Germany

**Keywords:** crocodiles, fast‐slow continuum, Rhynchocephalia, allometry, squamates, turtles

## Abstract

Allometric relationships linking species characteristics to body size or mass (scaling) are important in biology. However, studies on the scaling of life history traits in the reptiles (the nonavian Reptilia) are rather scarce, especially for the clades Crocodilia, Testudines, and Rhynchocephalia (single extant species, the tuatara). Previous studies on the scaling of reptilian life history traits indicated that they differ from those seen in the other amniotes (mammals and birds), but so far most comparative studies used small species samples and also not phylogenetically informed analyses. Here, we analyzed the scaling of nine life history traits with adult body mass for crocodiles (*n *=* *22), squamates (*n *=* *294), turtles (*n *=* *52), and reptiles (*n *=* *369). We used for the first time a phylogenetically informed approach for crocodiles, turtles, and the whole group of reptiles. We explored differences in scaling relationships between the reptilian clades Crocodilia, Squamata, and Testudines as well as differences between reptiles, mammals, and birds. Finally, we applied our scaling relationships, in order to gain new insights into the degree of the exceptionality of the tuatara's life history within reptiles. We observed for none of the life history traits studied any difference in their scaling with body mass between squamates, crocodiles, and turtles, except for clutch size and egg weight showing small differences between these groups. Compared to birds and mammals, scaling relationships of reptiles were similar for time‐related traits, but they differed for reproductive traits. The tuatara's life history is more similar to that of a similar‐sized turtle or crocodile than to a squamate.

## 1 INTRODUCTION

Allometric relationships linking species characteristics to body size or mass (scaling) are very common in biology (including paleobiology) because every aspect of life is more or less associated with body size and metabolic rate (Brown, Gillooly, Allen, Savage, & West, 2004; Kleiber, 1932; Peters, 1983; Schmidt‐Nielsen, 1984). After Snell's (1891) first use of an allometric equation describing the relationship between brain mass and body mass in mammals, subsequent studies unveiled the importance of allometric scaling not only for organs (e.g., blood, heart, lungs, skeleton), but also for physiological functions (e.g., locomotion, blood and gas transport, oxygen supply, temperature regulation), and even for ecological and evolutionary aspects of life (e.g., animal abundance, home‐range sizes, life history strategies; summarized in Peters, 1983 and Schmidt‐Nielsen, 1984). These investigations led ultimately to the discovery of a “fast–slow” continuum in mammalian life histories, where species are arranged on a single body mass axis with small, early maturing, highly fecund, and short‐lived mammals on the “fast” side and large mammals with opposite trait characteristics on the “slow” side of the continuum (Stearns, 1983).

The fundamental relevance of allometric relationships was recently confirmed and refined by the metabolic theory of ecology (MTE; Brown et al., 2004). The MTE relies on a ¾ power scaling of resting (basal) metabolic rate with body mass and utilizes an Arrhenius approach to model differences in metabolic rates of similar‐sized species by differences in their body temperature. The MTE “predicts how metabolic rate, by setting the rates of resource uptake from the environment and resource allocation to survival, growth, and reproduction, controls ecological processes at all levels of organization from individuals to the biosphere” (Brown et al., 2004).

**Figure 1:**
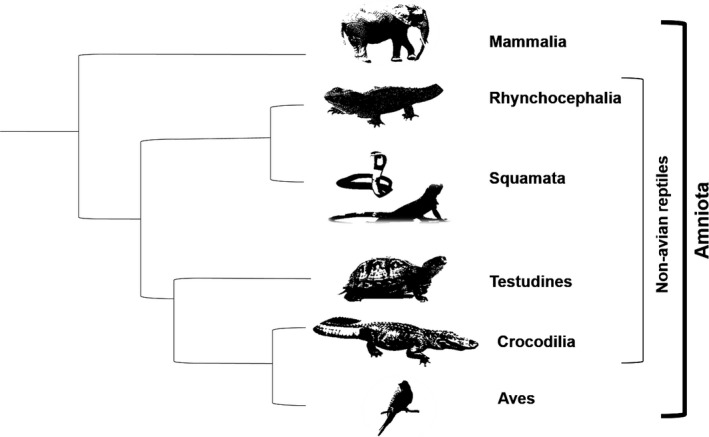
Cladogram of the tetrapod clade Amniota. The clades Rhynchocephalia, Squamata, Testudines, and Crocodilia form together the paraphyletic (nonavian) reptiles. The other amniote clades Mammalia and Aves (birds) are also shown

Although most allometric analyses, especially those on life history traits, have been carried out for mammals (e.g., Bekoff, Diamond, & Mitton, 1981; Dobson, 1992; Dobson & Oli, 2007; Gittleman, 1985; Jones, 1985; Schmitz & Lavigne, 1984; Stearns, 1983; Swihart, 1984; Tuomi, 1980), the impact of body size on life histories has also been demonstrated for many other taxonomic groups (from viruses to mammals: Blueweiss et al., 1978; Peters, 1983; Schmidt‐Nielsen, 1984; in plants: Hendriks & Mulder, 2008). The majority of these studies did not correct for the shared evolutionary history of species, although phylogeny is known to strongly alter regression coefficients of allometric equations compared to ordinary regression analysis (e.g., Clauss, Dittmann, Müller, Zerbe, & Codron, 2014; Harvey & Pagel, 1991; Lemaître, Müller, & Clauss, 2014). The lack of phylogenetically corrected allometries in the literature is due that phylogenetic correction requires phylogenies of taxa. Detailed large‐scale phylogenies of amniote clades (Figure 1) have only become available in the last few years (Bininda‐Emonds, Gittleman, & Steel, 2002 for mammals; Jetz, Thomas, Joy, Hartmann, & Mooers, 2012 or Suh, Smeds, & Ellegren, 2015 for birds).

Studies on allometric scaling of life history traits in reptiles (the nonavian Reptilia: crocodiles, squamates, turtles and the tuatara, *Sphenodon punctatus*, Figure 1) are generally rather scarce and are mostly limited to squamates (“lizards”, snakes, and amphisbaenians—”worm lizards”; Andrews, Pough, Zoology, & Apr, 1985; Dunham & Miles, 1985; Dunham, Miles, & Reznick, 1988; Hallmann and Griebeler, 2015; Scharf et al., 2014; Stearns, 1984; Tinkle, 1969; Tinkle, Wilbur, & Tilley, 1970; Warne & Charnov, 2008). This is most probably due to that 96.3% (>9,000 species) of all reptilian species are squamates (Pincheira‐Donoso, Bauer, Meiri, & Uetz, 2013) and that lizards and snakes show considerable morphological and physiological differences (e.g., lizards possess eyelids, ear openings, and a fleshy tongue, while snakes cover eyes with fixed scales, do not possess ears, and have broad scales on the abdomen and a forked tongue). Previous studies on the scaling of life history traits in reptiles suggest that allometric relationships differ from those seen in similar‐sized mammals and birds (Promislow, Clobert, & Barbault, 1992; Werner & Griebeler, 2011). For example, allometric relationships on life history traits among lizards have smaller scaling exponents and become less significant with increasing clade age, while in mammals the exponents and the significance of the exponent and constant of the allometric power function increase with an increasing taxonomic level (Promislow et al., 1992). The life histories of some reptilian taxa also deviate from the fast–slow continuum described for mammals (Stearns, 1983). The lizard species of the family Lacertidae have small clutches but relatively large young and are placed at the “fast” end, and species with large clutches and small young are placed at the “slow” end of the continuum (Bauwens & Díaz‐Uriarte, 1997). Bauwens and Díaz‐Uriarte (1997) also emphasized that different life history strategies exist in lacertids that are linked to body size; for example, small species mature earlier than large species. A recent large‐scale comparative study comprising the whole squamate group and the tuatara (*Sphenodon punctatus*) revealed a significant lower impact of body size on longevity than it has in mammals and birds (Scharf et al., 2014).

In contrast to squamates, for the clades Crocodilia, Testudines, and Rhynchocephalia far less is known about their allometric scaling of life history traits with body mass. For the clade Crocodilia, so far the scaling of clutch size and egg mass was to the best of our knowledge only studied without correcting for phylogeny (Thorbjarnarson, 1996; Werner & Griebeler, 2013). Information on the allometric scaling of turtles’ life histories is mostly available for single species (e.g., Portelinha, Malvasio, Piña, & Bertoluci, 2013; Ryan & Lindeman, 2007), and in the few interspecific studies, small sample sizes and nonphylogenetic analyses were used (e.g., Werner & Griebeler, 2013). In some cases, authors used carapace length of turtles and tortoises instead of body mass (e.g., Elgar & Heaphy, 1989; Iverson, 1992; Wilbur & Morin, 1988) making it difficult to compare allometries derived to other taxa, for example, to birds or mammals, due to differences in body shapes of animals.

Allometric relationships on life history traits and body mass are not only informative in understanding differences in life history strategies seen between extant vertebrate groups. They are also important for extinct groups. Allometric regressions on extant taxa are routinely used as comparative models for characters preserved or to estimate characters being unpreserved in the fossil record (e.g., reproductive traits of dinosaurs, Werner & Griebeler, 2011, 2013). Thus, most accurate allometries (with body mass as a predictor) on extant reptiles as a whole or on particular reptile taxa, which were so far not available, are indispensable for their reliable application to extinct (nonavian) reptiles.

The clade Rhynchocephalia is nowadays only represented by one extant species, the tuatara, *Sphenodon punctatus* (Hay, Sarre, Lambert, Allendorf, & Daugherty, 2010), which obviously makes any allometric analysis on this group based on extant species impossible. Nevertheless, the tuatara is a perfect species to demonstrate how allometric equations can be used to gain insights into the exceptional life history strategy of extant and even extinct Rhynchocephalia within reptiles (Deckert et al., 1991). Almost all studies using nuclear and mitochondrial markers as well as morphological traits (e.g., Renesto & Bernardi, 2014) place the tuatara and thus Rhynchocephalia as sister group to squamates (summarized in Rheubert, Siegel, & Trauth, 2014), whereas only few others suggest a more basal position for Rhynchocephalia and place then between the turtles and crocodiles (Hedges & Poling, 1999; Jamieson, 2014; Lyson et al., 2012).

In this study, we established allometric relationships between life history traits and body mass for reptile clades. For the first time, we consistently used a phylogenetically informed approach for nine life history traits and studied different reptilian taxa (Squamata, snakes, lizards, turtles, crocodiles, and all reptiles). We aimed at the following questions:

1. Which life history traits show an allometric relationship to body mass in reptiles? We therefore first explored whether there are differences in these relationships between the reptilian clades Crocodilia, Squamata, and Testudines (Figure 1). And also whether there are even differences in relationships within these clades, that is, have the paraphyletic lizards from the species‐rich squamates (Lacertilia, Günther 1867; comprising the squamate clades Dibamidae, Gekkota, Scincomorpha, Lacertoidea, Iguania, and Anguimorpha) and the squamate clade snakes (Serpentes, Linnaeus 1758) similar allometries on life history traits, or do these differ between lizards and snakes?

As we observed virtually no differences in finally established allometric relationships between life history traits and body mass at these lower taxonomic levels of reptiles, we further asked:

- Are there differences in the allometric relationships of life history traits between different amniote groups? Do reptiles as a whole differ in their relationships from mammals and birds, which form together with the crocodylians the taxon Archosauria (Lecointre & Guyader, 2005), but evolved considerably differing lifestyles?

We finally discuss our allometric relationships on reptiles, in the context of a fast–slow continuum of life history strategies (Stearns, 1983), the Metabolic Theory of Ecology (Brown et al., 2004), and with respect to the degree of the exceptionality of tuatara's (*S. punctatus*) life history, and thus of the clade Rhynchocephalia within reptiles (Deckert et al., 1991).

## 2 MATERIAL AND METHODS

### 2.1 Data collection

We conducted an extensive search for data on life history traits and adult weight for reptilian species (Table S1). We used primary literature (e.g., Thorbjarnarson, 1996 for crocodiles; Meiri, 2010 and Feldman & Meiri, 2013 for squamate body sizes), encyclopedias (e.g., for reptiles: “Handbuch der Reptilien und Amphibien Europas,” Böhme, 1981, 1984, 1986, 1993; Bischoff, 1998; Böhme, 1999; Fritz, 2001, 2005; Joger & Stümpel, 2005; for turtles: “Turtles of the World”, Ernst & Barbour, 1989), textbooks for particular reptile groups (e.g., for geckos: Henkel & Schmidt, 1991; Rogner, 1992; Rösler, 1995), as well as the Internet databases AnAge, which provides additional information (mainly on maximum longevity and age at maturity) on worldwide distributed reptilian species (Tacutu et al., 2013), and The Animal Diversity Web (ADW, http://animaldiversity.org, Myers et al., 2015). We focused on nine life history traits which cover the complete life of an animal: egg weight (g), clutch size, number of clutches per year, incubation/gestation time (days), birthweight (g), birth size (total length, cm), age at female maturity (days), size at maturity (cm), and maximum longevity (years). We selected adult weight (g) instead of body length (total length or snout‐vent‐length) as a measure of animal adult size to make our allometries on reptiles independent of animals’ body shapes and to allow a comparison of allometries to the respective ones of mammals and birds. Anecdotal remarks (without any reference) on species’ life history traits given in these data sources were not taken into account. When we found ranges or multiple values on traits for a species, values were always averaged for subsequent statistical analyses. In total, we collected data on the aforementioned nine life history traits from 743 species. Information on adult weight was finally only available for 369 reptile species (294 squamates, 52 turtles, 22 crocodiles, and the tuatara, *Sphenodon punctatus*). We used this dataset on all reptile species (Table S1) without the tuatara (*n *=* *368) for all analyses conducted on reptiles.

Squamates: Within squamates, we finally selected the lizards as a study group. This is based on the traditional view of the group Lacertilia (Günther, 1867). Lacertilia is defined as all extant members of the Lepidosauria that are neither sphenodonts nor snakes. The allometric analyses of life history traits of the squamate clades Dibamidae, Gekkota, Scincomorpha, Lacertoidea (including Amphisbaenia), Iguania, and Anguimorpha turned out to be statistically problematic due to small sample sizes on species obtained by us for clades (results on these clades are found in Tables S8 through S12). Data on life history and adult weight were available for 294 squamate species: 173 lizards (58.8% of the species covered in our database), 119 snakes (40.5%), and two amphisbaenids (0.7%). Most lizards in our dataset belonged to the families Lacertidae (*n *=* *29), Gekkonidae (*n *=* *22), and Scincidae (*n *=* *19). Most snakes were from the families Colubridae (*n *=* *35) and Viperidae (*n *=* *35). According to a recent assessment of the global diversity of living reptiles, 96.3% of all reptiles are squamates (59% lizards, 35% snakes, and 2% amphisbaenians; Pincheira‐Donoso et al., 2013). Most of the lizards belong to the families Scincidae and Gekkonidae, while most snake species belong to the family Colubridae (Pincheira‐Donoso et al., 2013). Our dataset covered very well the global squamate diversity, with only the amphisbaenids being underrepresented (*n *=* *2) at the clade and family level.

Turtles: We collected data on life history traits and adult weight of 52 turtle species. Thus, 17% of all 327 currently known turtle species on earth were represented in our dataset (Pincheira‐Donoso et al., 2013). Most of our turtle species were from the clade Cryptodira (*n *=* *50) and the family Testudinidae (*n *=* *35). The families Cheloniidae and Emydidae accounted for five species each. Only two species were from the clade Pleurodira: *Podocnemis expansa* and *Podocnemis unifilis* (family Podocnemididae). From the most species‐rich turtle family Geoemydidae (Pincheira‐Donoso et al., 2013), no species was in our dataset.

Crocodiles: Data on life history traits and adult weight were available for 22 crocodile species. From the species covered in the phylogeny of Oaks (2011), only *Caiman yacare* was missing in our dataset. In their global assessment of living reptile species, Pincheira‐Donoso et al. (2013) considered two crocodile species absent in the phylogeny of Oaks (2011): *Crocodylus raninus* (Ross, 1992) and *Crocodylus suchus*. The latter taxon was formerly assigned to *Crocodylus niloticus* and is now thought to be a distinct species (Hekkala et al., 2011). As the systematic status of *C. raninus* and *C. suchus* is highly controversial (Uetz & Hošek, 2013) and for these two new crocodile species information on their life history traits is not available, we did not consider them in our study. Unfortunately, we were unable to gather information on all nine life history traits of crocodilian species. Information on birth size was only available for six species, on birthweight for four species and on the number of clutches per year for two species. Thus, for crocodiles, we could finally analyze only six of the nine life history traits studied in the other reptile clades.

Geographic distribution: To account for differences in the geographic distribution of reptile species and their potential influence on life history traits of species, we additionally collected for each species information on the maximum altitude (m) of their areas inhabited (Adolph & Porter, 1993; Hodges, 2004; Tinkle & Gibbons, 1977). Information on maximum altitude was extracted from various sources (see Supporting information), except for the crocodilian species where it was mainly taken from ADW. We preferred altitude over latitude because elevational reptile richness most strongly correlates with temperature (McCain, 2010). Latitudinal‐scale studies have shown that richness correlates with precipitation, which in turn correlates with temperature (McCain, 2010).

### 2.2 Data analyses

All statistical analyses were performed with the statistical software R v3.0.2 (R Development Core Team, 2013) and additional packages (see below) available for this software. Initially, we tested for a correlation between maximum altitude and each life history trait for species, by taking into consideration body mass. We therefore established for each of the reptile groups generalized least squares linear regression models (GLS) for each of the life history traits (log_10_‐transformed). For these models, we used maximum altitude and adult weight (both log_10_‐transformed) as independent variables and applied multivariate regression analysis without and with phylogenetic correction (see next section). The nonphylogenetic analyses (Tables S2 through S7) enabled us to compare our results to previous studies. We pooled lizards (including the two amphisbaenian species) and snakes as Squamata (see Figure S2 for taxa covered by the clade Squamata) for our further analyses. Our allometric regression analyses had revealed no differences in the scaling of life history traits between both clades (see Results). Overall, we found no qualitative differences between the results obtained from phylogenetically informed and nonphylogenetically informed regression models for the reptiles as a whole and for lower taxonomic levels (Figure 4, Tables S2 through S7).

Allometries on life history traits of different reptilian taxa: For each of the different reptilian clades (Squamata, Testudines, Crocodilia) and for the two squamate groups, the lizards, and snakes, we established phylogenetic univariate linear regression models to investigate the relationship between each of the life history traits (log_10_‐transformed) and adult weight (log_10_‐transformed).

We therefore used the function *gls* from the R‐package *nlme* (Pinheiro, Bates, DebRoy, & Sarkar, 2015). This method of generalized least squares (GLS) relaxes the assumption that observations have the same variance, and that the covariance equals zero (Paradis, 2011). The latter is important because observations on species are never statistically independent, they correlate due to the species’ shared evolutionary history. Thus, closely related species are expected to produce more similar residuals from a regression line than less closely related. Nevertheless, the GLS method also allows correcting for phylogeny during the fitting process by considering a specific variance–covariance matrix that captures the correlation structure in a trait among species.

To estimate the variances of this matrix, we used the residual maximum likelihood (REML) fitting method provided by the function *gls*, because it enables an estimation of regression coefficients prior to the calculation of the variance. Our trait evolution model used to estimate the variances was the Brownian‐motion model as modified by Pagel (1999). We chose this model because a comparison of intercept‐only models with different models of trait (here adult weight) evolution resulted in the best models (smallest AIC value) for all studied reptile groups (Table S13). For the Brownian‐motion model, the covariance between species *i* and *j* is *v*_*ij*_ **=* *σ^*2*^ *d*_*a*_; with *d*_*a*_ the distance between the root and the most common recent ancestor of species *i* and *j*, and σ^*2*^ the variance of the Brownian process (Paradis, 2011). In Pagel's model version, the off‐diagonal elements of the variance–covariance matrix are additionally multiplied by the parameter λ (Pagel, 1999) describing the strength of the phylogenetic signal. We created this phylogenetic correlation structure with the function *corPagel* (Paradis, Claude, & Strimmer, 2004) and used it in the function *gls*. Depending on the species sample, we generated the phylogenetic trees used in the function *corPagel* (Paradis et al., 2004) by pruning already published phylogenies of the different reptile groups under study.

The phylogenetic tree of squamates was extracted from a recently published maximum likelihood tree of 4161 species (Pyron & Burbrink, 2014). This time‐calibrated molecular tree (seven nuclear genes, five mitochondrial genes) covers all currently known squamate families and subfamilies. For the phylogenetic tree of the crocodilians, we also used a time‐calibrated tree, which has 23 species and is based on the interspecific variability of four mitochondrial loci and nine nuclear loci (Oaks, 2011). We used the program TREETHIEF v. 1.0 (Rambaut, 2000) to extract this majority‐rule consensus tree from this publication. We did not consider the separation of *Crocodylus niloticus* and *Osteolaemus tetraspis* into two distinct species as proposed by Oaks (2011) because life history data were only available for (the old) *C. niloticus* but not for *Osteolaemus tetraspis* and the new *C. niloticus*. We thus accordingly modified the extracted tree via branch deletion for our analyses (see Figure S1 on the crocodilian tree). For the turtles, we used a maximum likelihood tree based on mitochondrial and nuclear genes of 230 turtle species (Guillon, Guéry, Hulin, & Girondot, 2012). As this tree is not time‐calibrated and not ultrametric, we had to use weights for the *gls* function. Therefore, we first used the function *vcv.phylo* of the R‐package *ape* to compute the expected phylogenetic variances and covariances of a continuous trait given a respective phylogenetic tree, thereby assuming it evolves under a Brownian‐motion model. We next extracted the diagonal values of the computed variance–covariance matrix with the function *diag* from the package *base*. We finally set the diagonal of the variance–covariance matrix as the fixed variance weights for the GLS model with the constructor‐function *varFixed* to correct for different root to tip lengths (i.e., in a nonultrametric tree root to tip lengths differ between species) within the phylogenetic tree of the turtles (Revell, 2012).

In the remaining text, we use the abbreviation GLS for nonphylogenetically informed regression analysis, and PGLS for phylogenetically informed regression analysis (GLS with any variance–covariance matrix correcting for the shared evolutionary history of species). To compare allometric models between different reptilian taxa, we used the 95% confidence intervals of their estimated slopes and intercepts. We applied the function *intervals* from the R‐package *nlme* to calculate these intervals.

Life history allometries of reptiles and their comparison with mammals and birds: For all reptiles (*n *=* *369, without the tuatara)*,* we analogously established phylogenetic and nonphylogenetic univariate linear regression models relating each of the life history traits (log_10_‐transformed) to adult weight (log_10_‐transformed). For phylogenetic models on reptiles, we first created a composite tree from the phylogenetic tree of squamates (Pyron and Burbrink, 2014), of crocodiles (Oaks, 2011), and of turtles (Guillon et al., 2012). As a basis for merging the three different trees being based on different markers, we used the cladogram given in Pincheira‐Donoso et al. (2013, Figure S2). We then created a topology where all branch lengths were set to unity to make the branch lengths comparable between the individual trees (see Supporting Information for the general reptile topology). This final topology was used in the phylogenetic regression analyses on all reptiles.

To compare life history allometries on all reptiles to the respective seen in mammals and birds, we searched for allometric equations in the literature. Only those equations on mammals and birds were used that met at least two of three criteria: A large sample size, a current publication, and a recent phylogeny were used for phylogenetic correction (Table 1).

**Table 1:** Allometric equations on life history traits of mammals and birds

Trait	Taxonomic group	Coefficients	Sample size	Phylogenetic correction	Reference
Clutch size	Birds (Paleognathae, Galliformes, Anseriformes)	*s* = 0.06, *i* = 0.82	*n* = 116	PGLS	Werner and Griebeler (2011)
Clutch size (litter size)	Mammals	*s* = −0.05, *i* = 0.16	*n* = 353	PGLS	Werner and Griebeler (2011)
Egg weight (g)	Birds (passerine)	*s* = 0.746, *i* = 0	*n* = 74	Independent contrasts	Martin et al. (2006)
Egg weight (g)	Mammals (monotremata)	n.a.	*n* = 1^a^	n.a.	n.a.^a^
Age at maturity (days)	Birds	*s* = 0.303, *i* = 1.89^b^	*n* = 69	Independent contrasts	De Magalhães et al. (2007)
Age at maturity (days)	Mammals (without cetaceans)	*s* = 0.214, *i* = 1.98^b^	*n* = 606	Independent contrasts	De Magalhães et al. (2007)
Incubation time (days)	Birds	*s* = 0.167, *i* = 0.96^c^	n.a.	None	Rahn (1975) in Schmidt‐Nielsen (1984)
Incubation time (gestation time, days)	Mammals (eutherian)	*s* = 0.09, *i* = 1.72^c^	*n* = 1214	PGLS with Pagel's λ transformation	Clauss et al. (2014)
Maximum longevity (years)	Mammals	*s* = 0.127, *i *=* *1.18^c^; *s* = 0.170, *i* = 9.8	*n* = 938 (AnAge); *n* = 919 (Pantheria)	PGLS with Pagel's λ transformation	Lemaître et al. (2014)
Maximum longevity (years)	Birds	*s* = 0.218, *i* = 0.72^c^	*n* = 518	Independent contrasts	De Magalhães et al. (2007)
Size at maturity (cm)	Birds	*s* = 1, *i* = 0	n.a.	n.a.	Charnov (1993)
Size at maturity (cm)	Mammals	*s* = 1, *i* = 0	n.a.	n.a.	Charnov (1993)

*s*, slope; *i*, intercept; n.a., not available.

Listed are the taxonomic groups studied by authors, regression coefficients of allometries (log_10_‐log_10_‐transformed data), sample sizes on species analyzed, regression method used, and the reference for the study.

a A single data point for the monotreme echidna (egg‐laying mammal, family Tachyglossidae but species not given in the source Wildcare Australia Inc., http://wildcare.org.au/species-information/echidnas/, it is most probably *Tachyglossus aculeatus*, which is the only one known to live in mainland Australia, adult weight is 4500 g, egg weight is 1.75 g) was added to scatterplot in Figure 3.

b Original intercepts were multiplied by 365 herein for a transformation of age from years to days and then log_10_‐transformed (for original values, see De Magalhães et al., 2007).

c We log_10_‐transformed the original intercepts (for original values, see Rahn 1975, Clauss et al., 2014; Lemaître et al., 2014).

Application of reptile allometries to the tuatara: To assess the degree of the exceptionality of the tuatara's life history within today's reptiles, we compared eight life history trait values (we found no estimate on the tuatara's birth size in the literature, see Table S1) to the respective phylogenetically corrected regression line on squamates, crocodiles, and turtles. We therefore calculated for each reptile clade the residual distance, which is the value expected for a species of an equal body mass from the clade minus the tuatara value (Werner & Griebeler, 2013).

## 3 RESULTS

Differences in the geographic distribution of reptile species as assessed by maximum altitude had no significant effect on any of the life history traits of squamates, crocodiles, and turtles studied, and also not on all reptiles (results of multiple GLS regressions, see Table S14 in the Supporting Information). These analyses reconfirmed that for some of the life history traits (e.g., birthweight, clutch size or max. longevity) adult weight is an important predictor. We therefore excluded maximum altitude from our further analyses.

### 3.1 Allometric relationships of life history traits within the reptiles

We were able to establish phylogenetic and nonphylogenetic allometric regression models for all life history traits studied for both groups lizards and snakes and for the clades Crocodilia, Squamata, and Testudines. The 95% confidence intervals (CI) of scaling exponents (hereafter slopes) and constants (hereafter intercepts) of GLS and PGLS models indicated similarities and dissimilarities in the scaling of life history traits to adult weight for taxa.

**Figure 2:**
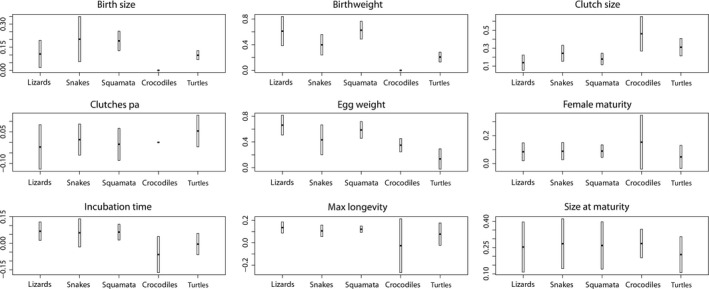
Estimated slopes (scaling exponents) with 95% confidence intervals obtained from phylogenetic regression analyses (PGLS, see Section 2) for different reptilian taxa. Equations of regression models (exact values of slopes) are found in Table S2 through S7. Missing bars indicate lack of data for the respective taxonomic group (only applicable to the crocodiles)

The groups lizards and snakes: The CIs of the slopes and intercepts from the phylogenetic regression models of lizards and snakes overlapped for all life history traits (Figures 1 and 2, Tables S3 and S4). This indicates no statistical difference between scaling of traits within both taxa.

**Figure 3:**
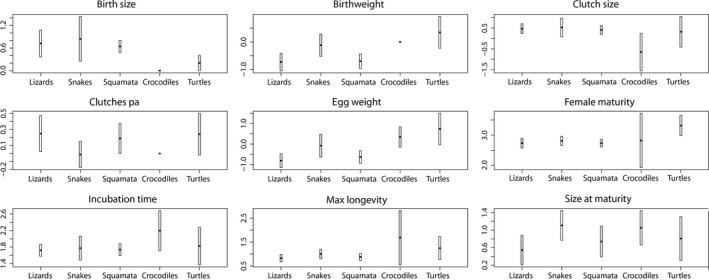
Estimated intercepts (scaling constants) with 95% confidence intervals obtained from phylogenetic regression analyses (PGLS, see Section 2) for different reptilian taxa. Equations of regression models (exact values of intercepts) are found in Table S2 through S7. Missing bars indicate lack of data for the respective taxonomic group (only applicable to the crocodiles)

The clades Crocodilia, Squamata, Testudines: For crocodiles and turtles, the CIs of slopes and intercepts overlapped for all life history traits studied (Figures 1 and 2). In all other pairwise comparisons of reptilian clades, differences were observed, except for birth size, birthweight, clutch size, egg weight, and age at female maturity (Figures 1 and 2). For birth size and birthweight, slopes and intercepts were only significant for squamates and turtles, whereas for crocodiles sample sizes were too small to establish a significant regression model on these traits (*n *=* *6, *n *=* *4, Table 4). The CI of the intercept of the birth size model differed between squamates (lower and upper value of CI intercept: 0.483, 0.806; Figure 3) and turtles (CI intercept: 0.007, 0.405; Figure 3). The CIs of the slope and intercept of the birthweight model differed between squamates (CI slope: 0.502, 0.772; CI intercept: −0.978, −0.457) and turtles (CI slope: 0.131, 0.276; CI intercept: −0.200, 0.922). For clutch size, only the CI of the slope differed between squamates (CI slope: 0.116, 0.243; Figure 2) and crocodiles (CI slope: 0.268, 0.653; Figure 2). For egg weight, the CI of the slope and intercept differed between squamates (CI slope: 0.457, 0.717; CI intercept: −0.944, −0.328) and turtles (CI slope: −0.022, 0.293; CI intercept: −0.023, 1.497), and also between squamates and crocodiles (CI slope: 0.246, 0.453; CI intercept: −0.147, 0.841). For age at female maturity, only the CI of the intercept differed between squamates (CI intercept: 2.613, 2.860; Figure 3) and turtles (CI intercept: 2.928, 3.647; Figure 3).

### 3.2 Comparison of life history allometries between reptiles, mammals, and birds

Because information on birth size, birthweight, and number of clutches per year were insufficient for crocodiles (see Section 2), overall reptile models were only established for clutch size, egg weight, incubation time, maximum longevity, age at female maturity, and size at maturity. A comparison of our allometric models to respective models published on birds and mammals (Table 1) revealed several differences between amniote taxa.

**Figure 4:**
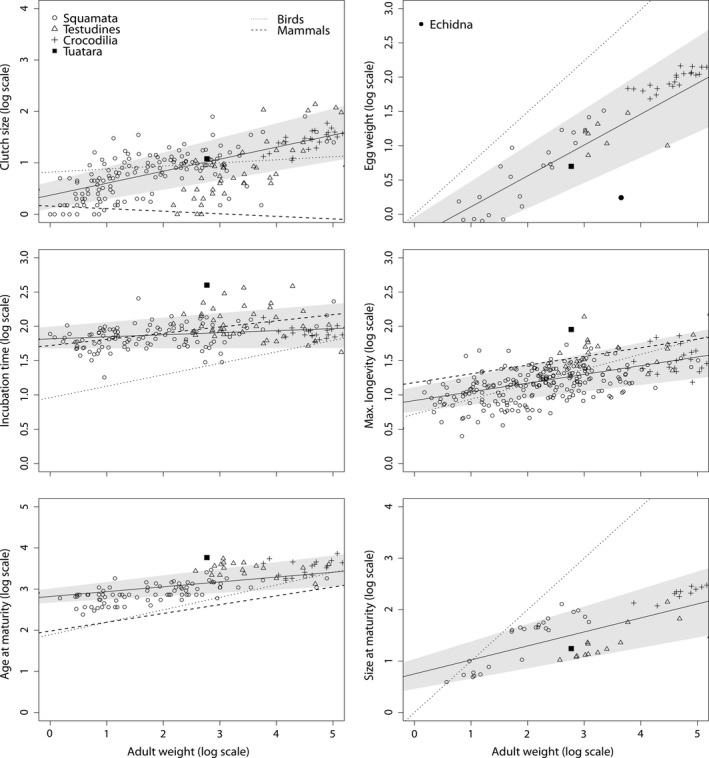
Scatter plots on life history traits and allometric relationships in reptiles, and respective relationships seen in mammals and birds. The gray areas mark the 95% confidence belts of phylogenetically corrected reptilian regression models. The dashed lines represent the respective phylogenetic relationships for reptiles. Regression lines for mammals and birds are taken from Table 1. In the plot on egg weight, the egg weight of an echidna (most probably *Tachyglossus aculeatus*) is included and represents the group egg‐laying Mammalia (filled circle, see text). As birds and mammals show determinate growth, the onset of sexual maturity is seen at full size, that is, size at maturity scales isometrically with adult weight (dotted line in this plot, slope = 1, intercept = 0; see main text for further information) in both taxa. In all plots, the filled square marks the tuatara, open diamonds mark species from Squamata, open triangles species from Testudines, and crosses species from Crocodilia

Clutch/litter size: The regression lines on clutch/litter size differed between reptiles, mammals, and birds (Figure 4, see also Table S2 for GLS and PGLS regression models; for allometric equations of mammals and birds see Table 1). The influence of adult weight on clutch size was strongest in reptiles (*s *=* *0.23, with *s* slope of the regression line). In birds (*s *=* *0.06, Table 1) and mammals, it was much weaker (*s *=* *−0.05, Table 1). While in reptiles and precocial, flightless birds (although not significant) clutch size increased with increasing adult weight, litter size did significantly decrease in mammals.

Egg weight: The effect of adult weight on egg weight was weaker in reptiles (*s *=* *0.45) than in birds (*s *=* *0.77; Figure 4 and Table 1). The egg weight of an echidna (Table 1) was considerably smaller than that of similar‐sized reptile (Figure 4).

Incubation time (gestation time): In contrast to mammals and birds, incubation time was independent of adult weight in reptiles (*s *=* *0.03, *p *=* *.06; Table 1 and Figure 4).

Maximum longevity: The slopes of the regression lines linking maximum longevity to adult weight were equal in reptiles (*s *=* *0.13) and mammals (*s *=* *0.13). They indicated a similar strong positive effect of adult weight on maximum longevity in both taxa. Compared to mammals and reptiles, maximum longevity increased the most with increasing adult weight in birds (*s *=* *0.22).

Age at female maturity: In reptiles (*s *=* *0.12), age at female maturity increased the least with increasing adult weight, the most in birds (*s *=* *0.303), and moderately in mammals (*s *=* *0.214).

Size at maturity: We found an allometry with a slope considerably smaller than one (*s* = 0.27) for reptiles, whereas in birds and mammals body mass and size at sexual maturity scaled isometrically with adult body mass (Table 1 and Figure 4).

### 3.3 The degree of exceptionality of the life history of the tuatara, *Sphenodon punctatus*, and thus of the clade Rhynchocephalia, within today's reptiles

The comparison of life history traits of the tuatara and those predicted from allometries for a similar‐sized crocodile, turtle, or squamate revealed no clear overall pattern (Figure 4). For the traits incubation time and maximum longevity, the residual values (differences between the logarithmized values and the values predicted by the regression lines for the body mass of the tuatara) were the lowest for the crocodile model (Table 2). For the traits clutch size, size at maturity, age at female maturity, and birthweight, the residual values were the lowest for the turtle model (Table 2). And for the traits egg weight and number of clutches per year, the residual values were the lowest for the squamate model (Table 2).

**Table 2:** Comparison of life history traits of the tuatara with those predicted from our allometries for a similar‐sized crocodile, turtle, or squamate

	Crocodile	Turtle	Squamate
Clutch size	0.46	0.10	0.18
Egg weight	0.62	0.40	0.29
Size at maturity	0.57	0.15	0.22
Age at female maturity	0.51	0.32	0.78
Incubation time	0.58	0.79	0.69
Max. longevity	0.34	0.49	0.74
Birthweight	–a	0.32	0.44
Number of clutches per year	–a	0.85	0.62

Shown are the residual values (the difference between the logarithmized value and the value predicted by the respective regression line from the tuatara's body mass) for eight life history traits.

Residual value in bold* *=* *smallest difference between predicted value and the tuatara's value.

a No allometry established on this trait for the taxon. Please note that information on birth size was not available for the tuatara (see Table S1).

## 4 DISCUSSION

### 4.1 Allometric relationships of life history traits within the reptiles

The clades Squamata, Crocodilia, Testudines (for a summary of results see Table 3): Although there is a good understanding of phylogenetic relationships among extant reptiles (Lecointre & Guyader, 2005), most of the previous allometric studies focused on different taxonomic levels.

**Table 3:** Summary table on allometric relationships of life history traits in reptiles (see Section 4.1)

Taxa	Birth size TL	Birthweight	Clutch size	Clutches p.a.	Egg weight	Female maturity	Incubation time	Max. longevity	Size at maturity
Squamata	0	+	0	–	+	0	0	0	+
Crocodilia	(*)	(*)	+	(*)	+	–	–	–	0
Testudines	+	+	+	–	–	–	–	–	+

The impact of body mass on nine different life history traits is shown for the clades Squamata, Crocodilia, and Testudines. +* *=* *strong impact (large regression slope) of body mass on the respective trait, 0 = moderate, –* *=* *weak or zero, and (*)* *=* *no information available. The information is taken from the phylogenetically informed regression models. More detailed information on allometric relationships of life history traits can be found in the main text or in the specific tables on the different reptile clades (see Tables S2–S7).

Squamates (lizards and snakes): In this study, we successfully established phylogenetic allometric regression models on nine life history traits for the squamate groups lizards and snakes. For all nine life history traits under study, the confidence intervals of slopes and of intercepts of allometries obtained did not significantly differ between lizards and snakes (Figures 1 and 2). This suggests that life history traits of lizards and snakes scale equally with adult body mass. When pooling the amphisbaenians, lizards, and snakes to Squamata, the allometric relationships of traits resembled again the respective seen in lizards and snakes (Table S7). Our results thus corroborate Stearns (1984) who hypothesized that the differences in life histories that he observed between lizards and snakes would disappear if the effect of body size is removed. Stearns (1984) also stressed a considerable phylogenetic effect on patterns of covariation in life history traits, but contrary to our study he did not address it. Dunham and Miles (1985), who questioned the results of Stearns (1984) (the authors corrected Stearns’ dataset), demonstrated a significant impact of phylogeny on the covariation in life history traits after removing body size effects. Shine (1994, 1996) investigated interspecific patterns in ecological traits of Australian snakes. He found high intercorrelations among reproductive traits such as clutch size and offspring size, but also with mean adult body size. He also figured out that these intercorrelations were not always due to phylogenetic conservatism. In contrast, a study on the offspring size/clutch size trade‐off and on reproductive allometries in lizards indicated that phylogenetic regression models fitted significantly better than nonphylogenetic models (Warne & Charnov, 2008).

Our results on squamates also indicate important quantitative differences in slopes and intercepts for some of the allometric relationships on life history traits when correcting for the shared evolutionary history of species or not (Table S7, significant λ values). They thus corroborate that phylogenetically informed regression analysis is preferable over ordinary analysis. In general, our phylogenetically informed allometries on the size‐related traits birthweight, egg weight, and size at maturity had the steepest slopes and these traits are thus strongly shaped by adult body weight. In contrast, our allometries on the time‐related traits age at maturity, incubation/gestation time, and maximum longevity had much shallower slopes, and thus, traits were less affected by adult body weight. Adult weight influenced the birth size and clutch size only moderately, and the number of clutches per year was even independent of adult body weight. Our results on a similar scaling of clutch size and age at maturity with adult weight in lizards and snakes contradict Stearns (1984) and Dunham and Miles (1985), but may be explained by that these authors did not use phylogenetically informed analyses.

Crocodiles: For crocodiles, we could only derive allometries on six of the nine life history traits, because we could not find enough information on species’ birth size, birthweight, and number of clutches per year. To the best of our knowledge, the allometric study of Thorbjarnarson (1996) is the only one dealing with a larger number of life history traits of crocodiles. This author established allometric relationships on reproductive traits based on 22 crocodile species but did not account for the phylogeny of species. The author argued that any phylogenetic control is inappropriate for such a small number of crocodile species, although he stated that a covariance between the independent variables was evident in his analyses. Thorbjarnarson (1996) observed significant positive slopes and significant nonzero intercepts for egg mass, clutch size, or clutch mass against adult female body size (*s *=* *0.29–0.72; *p *=* *.001). As expected, we reproduced his allometries on these traits under nonphylogenetically informed analyses, but we observed differences between phylogenetically and nonphylogenetically informed analyses for clutch size and egg weight. For both traits, our phylogenetic slopes were indeed positive, but slopes differed significantly from those estimated by Thorbjarnarson (1996) (clutch size: *s*_GLS_ **=* *0.348, *s*_PGLS_ **=* *0.461, *p *<* *.001; egg weight: *s*_GLS_ **=* *0.295, *s*_PGLS_ **=* *0.350, *p *<* *.001), whereas intercepts did not (clutch size: *i*_GLS_ **=* *−0.152, *p*_GLS_ **=* *0.684, *i*_PGLS_ **=* *−0.655, *p*_PGLS_ **=* *0.142; egg weight: *i*_GLS_ **=* *0.158, *p*_GLS_ **=* *0.010, *i*_PGLS_ **=* *0.347, *p*_PGLS_ **=* *0.158). In the study of Thorbjarnarson (1996) and our study, size and age at maturity showed a positive allometric relationship, but our slope on age at female maturity derived under phylogenetically informed analysis did not significantly differ from zero (*p *=* *.110). In contrast to Thorbjarnarson (1996), under phylogenetically informed analysis, we found that both incubation time and maximum longevity are independent of adult weight (incubation time: *s *=* *−0.064, *p *=* *.203; maximum longevity: *s *=* *−0.026, *p *=* *.822). As smaller animals generally face a higher predation risk than larger ones (Owen‐Smith, 1988), this was unexpected. Fully grown crocodiles are nearly safe from predation. Only territorial struggles with other crocodiles cause mortalities among fully grown animals (Pooley & Ross, 2002). This could explain that maximum longevity is independent of adult weight in crocodiles. In contrast, during their first year of life crocodile hatchlings experience mortalities of up to 90% due to predation (Pooley & Ross, 2002). Mortality is thus more or less independent of hatchling size over the adult body weight range covered by crocodiles. An increase in hatchling size would imply larger eggs and an increase in incubation time.

Turtles: For turtles, our results indicated no clear allometric patterns in life history traits. Egg weight, clutch size, birth size, birthweight, and size at maturity scaled positively with adult body mass. Among these traits, only the slope of the allometry on egg weight was not significant (*p *=* *.082). Slope values for the traits number of clutches per year, age at female maturity, incubation time, and maximum longevity indicated that these were independent of or only weakly dependent on adult weight. However, none of the small slopes was significant.

Many studies on the allometric scaling of life history traits in turtles have already been conducted (e.g., Congdon & Gibbons, 1985; Elgar & Heaphy, 1989; Iverson, 1992; Iverson, Balgooyen, Byrd, & Lyddan, 1993). However, most of these studies have the same limitations as those on crocodiles. Information on life history traits and body size (usually carapace length) was restricted to a few species (e.g., Congdon & Gibbons, 1985; Elgar & Heaphy, 1989; Iverson, 1992; *n *=* *12–35; but see Wilbur & Morin, 1988; Iverson et al., 1993), only a few of the traits (mostly clutch size, clutch mass, and egg size) studied by the authors were already analyzed by other authors, and most authors did not take into consideration the shared evolutionary history of species in their analyses. Nevertheless, all those previous studies on turtles found a significant, positive relationship between body size and the traits studied herein (Congdon & Gibbons, 1985: egg mass, clutch size, clutch mass; Wilbur & Morin, 1988: egg mass, clutch size, clutch mass; Elgar & Heaphy, 1989: egg weight, clutch size; Iverson, 1992: egg mass, clutch size, clutch mass, annual clutch mass, age at maturity; Werner & Griebeler, 2013: egg mass, clutch mass in tortoises). While our results on the allometric relationship between clutch size and adult body mass corroborate these previous studies, those on all other traits contradict. The latter could be explained by that species samples studied differ in size and composition, the use of nonphylogenetically informed analysis (except for age at maturity), and the use of body size (carapace length) instead of adult weight (used here) in previous studies. The relation of birth size, birthweight, incubation time, or maximum longevity to adult weight has so far not been investigated in turtles. Only for the trait age at maturity, Iverson (1992) found a significant, positive relationship with body mass (*i *=* *0.78, *s *=* *2.25, *p *<* *.001, *n *=* *35), but he did not control for the shared evolutionary history of species in his regression analysis. His observation contradicts our phylogenetically informed but also our nonphylogenetically informed analysis as both indicate that age at maturation is independent of adult body weight. However, our confidence intervals of estimated slopes are large (Figure 2). This could reflect a considerable effect of environmental variability on sexual maturation in species that in turn could cover a smaller effect of adult body mass on this trait. Not only the life history of turtles but also their latitudinal distribution correlates inversely with body size (Iverson, 1992; Wilbur & Morin, 1988).

Homogeneity of allometric scaling relationships in reptile clades: Our analysis on differences in scaling of life history traits with adult weight between Squamata, Crocodilia, and Testudines (see Table 3) was only based on six traits for which sufficiently large samples were available for all clades (birth size, birthweight, and number of clutches per year had to be excluded). For most of these traits, the 95% confidence intervals (CI) of the slopes and intercepts overlapped for all three reptile clades (CI of female maturity, incubation time, maximum longevity, and size at maturity overlap; Figures 1 and 2). Crocodiles and turtles showed for none of the six traits any difference in scaling with body mass. For clutch size, only the CI of the slope differed between squamates and crocodiles. For egg weight, the CI of the slope and of the intercept of squamates differed from those seen in crocodiles and in turtles. These statistical differences in the allometries on clutch size and egg weight between squamates, crocodiles, and turtles were presumably caused by large differences in sample sizes available for clades (clutch size: squamates, *n *=* *137, crocodiles* *=* *22, turtles* *=* *49; egg weight: squamates* *=* *29, crocodiles* *=* *22, turtles* *=* *10). Larger sample sizes are expected to lead to narrower confidence intervals of estimated slopes and intercepts, which in turn increases the probability to find significant differences between slopes and intercepts. Hence, although we observed small differences in the regression coefficients between the squamate models and the crocodile or turtle models, we are convinced that scaling relationships on the six life history traits and adult body mass are reliable and that they can be compared to the respective seen in birds and mammals (Table 1).

### 4.2 Comparison of life history allometries between reptiles, mammals, and birds

Compared to birds and mammals, our life history allometries across reptiles showed similarities for time‐related traits (age at female maturity, maximum longevity) and differences for reproductive traits (clutch size, incubation time resp. gestation time), whereas size‐related traits are not comparable (size at maturity, egg weight) between amniote groups.

Reptiles, mammals, and birds showed a positive allometry for age at maturity, but slopes considerable differed between them (reptiles: *s *=* *0.118, mammals: *s *=* *0.214, birds: *s *=* *0.303). For reptiles, a positive slope is corroborated by a study on snakes and lizards by Shine and Charnov (1992). These authors found that the instantaneous mortality rate is inversely proportional to age at maturity. If we assume that larger reptile species are older at maturation than smaller reptile species, then larger species should have a lower mortality rate than smaller. For maximum longevity, the slopes were equal in reptiles and mammals (0.127 for both), but the intercepts differed clearly (reptiles: *i *=* *0.918, mammals: *i *=* *1.72). Thus, mammals have in general a higher maximum longevity than similar‐sized reptiles, and this result is corroborated by Western and Ssemakula (1982). These authors found that birds have a higher maximum longevity than similar‐sized mammals and that these, in turn, have a higher maximum longevity than similar‐sized reptiles. Our result is also supported by one of the mammalian datasets studied by Clauss et al. (2014), whereas their other dataset analyzed suggests a considerably higher slope (*s *=* *0.170) than that estimated by us for reptiles.

For clutch size and incubation/gestation time, allometries differed considerably between reptiles, birds, and mammals. We found a positive relationship between clutch size and adult weight for reptiles, whereas Werner and Griebeler (2011) found no significant effect of body mass on clutch size in precocial flightless birds and a slightly negative scaling in mammals under ordinary regression analysis. For reptiles, we observed that incubation time is independent of adult body weight. In contrast, in birds and mammals, incubation/gestation time scales positively, with the slope being steeper in birds than in mammals (Rahn, Paganelli, & Ar, 1975). However, the allometric bird model is not well constrained as Rahn et al. (1975) used ordinary regression analysis and provided no information on the number of species analyzed.

Our observed differences in the scaling of size at maturity between reptiles, birds, and mammals reflect their different growth strategies. Birds and mammals show determinate growth and reach sexual maturity when they are more or less fully grown (except for the largest mammals, e.g., elephants; Owen‐Smith, 1988). This results in an isometric relationship (*s *=* *1) between size at maturity and adult weight. In contrast, reptiles show indeterminate growth and grow considerably even after having reached sexual maturity (Reiss, 1989; Ritz, Griebeler, Huber, & Clauss, 2010). They thus show a weaker, positive nonisometric relationship (*s *=* *0.272).

For egg weight, the impact of adult weight is weaker in reptiles (*s *=* *0.450) than in birds (*s *=* *0.746). Interestingly, the egg weight of the monotreme echidna (family Tachyglossidae) is considerably smaller than expected under the reptile model and also much smaller than that of a similar‐sized bird (Figure 4). When we assume that this echidna is representative for Monotremata in general, this would suggest that the relationship between egg weight and adult weight is significantly weaker in Monotremata than in reptiles or birds.

The fast–slow continuum of life history traits of reptiles: Stearns (1983) suggested that smaller mammals mature early and have large litters, whereas large species mature late and have small litters. For squamates, he proposed a similar continuum on life histories where at the one end small, early maturing species with small, but frequent clutches per year are found, and at the other end the large, late maturing species with large, but few clutches per year are seen. For lizards, his prediction was confirmed by Promislow et al. (1992). These authors observed that the absolute value of allometric slopes of life history traits decreases with increasing clade age and that slopes are less significant at higher taxonomic levels. Consistent with the observation of Promislow et al. (1992), we observed no differences in slopes between the reptilian clades and also none between lizards and snakes (Squamata). Our results also suggest associations in life history traits in reptiles that are contrary to those seen in mammals (Stearns, 1983): Small reptiles mature earlier at smaller body sizes, have smaller clutches that are incubated for a shorter period and yield smaller hatchlings, and have a shorter lifespan than larger species showing the opposite traits. Our results thus strongly question the hypothesis that the life histories of squamates conform to the fast vs. slow continuum seen in mammals (Stearns, 1983).

Metabolic theory of ecology (MTE): The MTE refers to the relationship between body size and body temperature to metabolic rate across all organisms (Brown et al., 2004). The metabolic rate is considered to be the fundamental constraint by which all ecological processes are governed, and it is assumed to scale with body mass with an allometric exponent of 0.75 (Brown et al., 2004). Our scaling exponents (slopes) of allometric relationships found for lizards, snakes, Squamata, crocodiles, and turtles are more or less inconsistent with those predicted by the MTE (Brown et al., 2004). While only two (birthweight and egg weight) size‐related traits (Figure 2) show a scaling exponent of about 0.75, in lizards, snakes, and Squamata, only one time‐related trait (female maturity, Figure 2) in crocodiles shows an exponent consistent with the expected exponent of 0.25.

### 4.3 The degree of exceptionality of the life history of the tuatara, *Sphenodon punctatus*, and thus of the clade Rhynchocephalia, within the reptiles

Paleontological studies have shown that the clade Rhynchocephalia was widely distributed and morphological diverse in the Mesozoic (Herrera‐Flores, Stubbs, & Benton, 2017; Jones et al., 2013). Today, only one living species, the tuatara *Sphenodon punctatus*, is found on a few geographically isolated islands that are located along the coast of New Zealand (Hay et al., 2010). The species is considered to be a phylogenetic relict (Hugall, Foster, & Lee, 2007) or a living fossil, having preserved characteristics of early reptiles (Herrera‐Flores et al., 2017).

With respect to its life history, it is known that the tuatara has a long incubation time, the highest age at maturity across all known living reptiles, and a high life expectancy when compared to today's reptiles (Deckert et al., 1991). When comparing the tuatara's traits to those of a similar‐sized crocodile or turtle, six of eight life history traits (we had no estimate on its birth size, Table S1) conform better to similar‐sized species from these two reptile clades, but fewer traits to a similar‐sized squamate (see Table 2). The better fit of the tuatara's life history to crocodiles and turtles than to squamates was also corroborated in a principal component analysis (PCA). This PCA was conducted with values of six life history traits of species from these three reptile clades (Figure S3; for crocodiles, information on birth size, birthweight, and number of clutches was missing for the majority of species). These two findings are not consistent with our current understanding of the phylogenetic position of rhynchocephalians within reptiles.

Almost all molecular and morphological studies suggest that the tuatara and, thus, the rhynchocephalians are a sister group of squamates (Cree, 2014). Only a few studies like those on the structure of spermatozoa (Jamieson, 2014), microRNAs (Lyson et al., 2012), or six nuclear protein‐coding loci (Hedges & Poling, 1999) corroborate the more basal position of rhynchocephalians within the Amniota which is more consistent with the tuatara's life history traits.

On the one hand, a life history strategy is the result of an interaction of a huge amount of genes, whereas molecular studies mostly only examine comparative short DNA sequences (Crawford et al., 2012; Hugall et al., 2007; Pyron, Burbrink, & Wiens, 2013). On the other hand, it is questionable whether the tuatara is indeed a good representative of Rhynchocephalia in general. The tuatara is carnivorous and terrestrial, while some Mesozoic rhynchocephalians were herbivorous and some were aquatic (Cree, 2014). Thus, its life history could not be typical for all Mesozoic rhynchocephalians (Reynoso, 2000; Reynoso & Clark, 1998). Moreover, other squamate species, for example, geckos, living on the same islands, and in the same habitat, have evolved a life history similar to that of the tuatara (Dawbin, cited in Deckert et al., 1991). This could indicate that such a life history is an adaption to specific local environmental conditions. For incubation time, the better fit of the tuatara to the crocodile than to the reptile or turtle model was unexpected because the poorly developed tuatara embryo in a newly laid egg resembles more the situation seen in turtles (Cree, 2014).

Overall, our analysis showed that the life history of the tuatara is not necessarily exceptional within the reptiles, rather than that the life history of the Squamata seems to be autapomorphic.

## CONFLICT OF INTEREST

None declared.

## AUTHOR CONTRIBUTIONS

KH collected and analyzed the data and wrote the first draft of the manuscript. KH and EMG jointly conceived the project, designed the study, and interpreted the results. KH and EMG equally provided editorial assistance and gave final approval for publication.

## DATA ACCESSIBILITY

All data used in this manuscript are listed in the manuscript and in its Supporting Information.
