# Supplementary material for: An exploration of differences in the scaling of life history traits with body mass within reptiles and between amniotes
Source: Ecol Evol. 2018 May 2;8(11):5480–94. doi: 10.1002/ece3.4069 (PMC6010814; doi:10.1002/ece3.4069)
Supplement: Supplementary file 1 [file ECE3-8-5480-s001.docx]

Supporting information


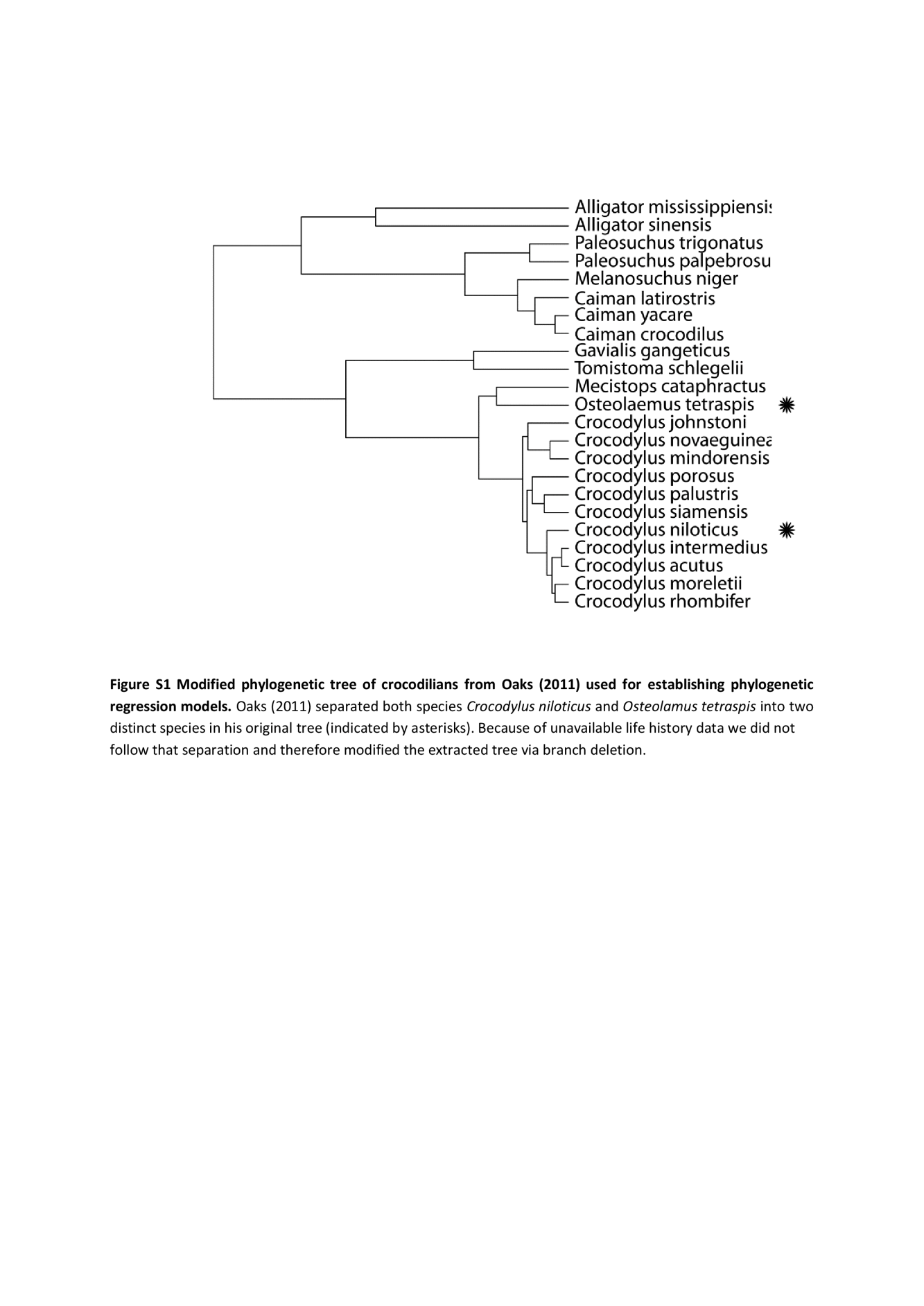


Figure S1 Phylogenetic tree on crocodilians from Oaks (2011) that is the basis of our tree used for establishing phylogenetic regression models. Oaks (2011) considered *Crocodylus niloticus* and *Osteolamus tetraspis* as two distinct species (indicated by asterisks). Because life history data on *Osteolamus tetraspis* and the new *Crocodylus niloticus* is unavailable, we did not follow his separation and therefore extracted *Osteolamus tetraspis* tree via branch deletion. Branch deletion was also done for *Caiman yacare*. For this species insufficient information on its life history traits was available to us.


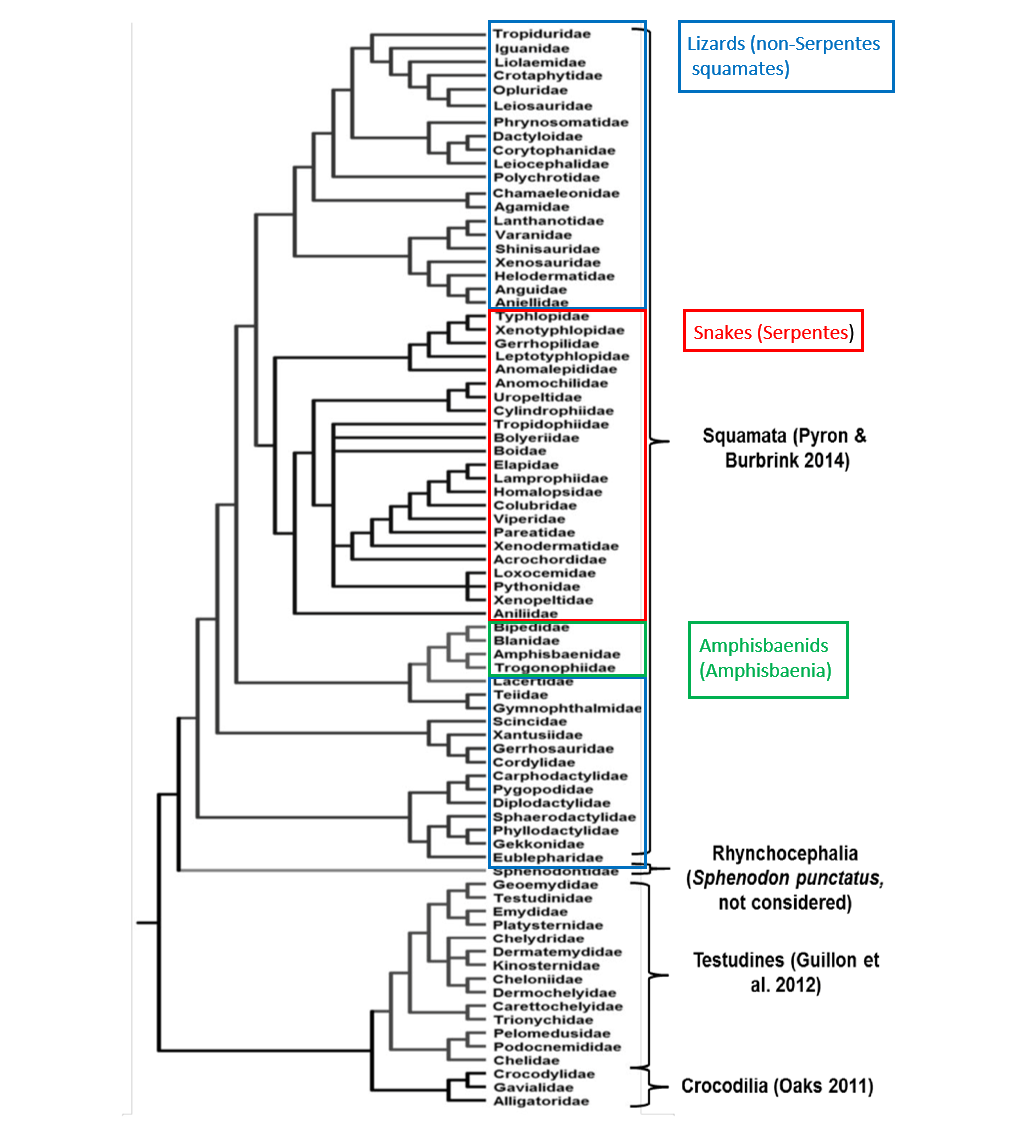


Figure S2 The phylogenetic tree from Pincheira-Donoso et al. (2013). It is the basis of our composite tree/topology on reptiles. Colors indicate the different reptile clades (blue = lizards (non-Serpentes squamates), red = snakes (Serpentes), green = amphisbaenids, black = Testudines/Crocodilia). The references for group-specific phylogenetic trees are also given. To establish our composite tree species listed in the group-specific phylogenetic trees (on Squamata, Testudines, Crocodila) were assigned to the respective families listed in the tree of Pincheira-Donoso et al. (2013). Please note that in our composite tree all branch lengths were set to unity to make the branch lengths comparable between the merged trees on clades. The family Sphenodontidae, and thus *Sphenodon punctatus*, was excluded from our reptile tree to avoid problems with a predetermined phylogenetic position of this species (including the tuatara could have changed the scaling exponents and constants). Birds and other groups covered in the original tree of Pincheira-Donoso et al. (2013) are excluded in the tree shown. Our composite tree on reptiles is coded in Newick file format and available on demand from the authors.


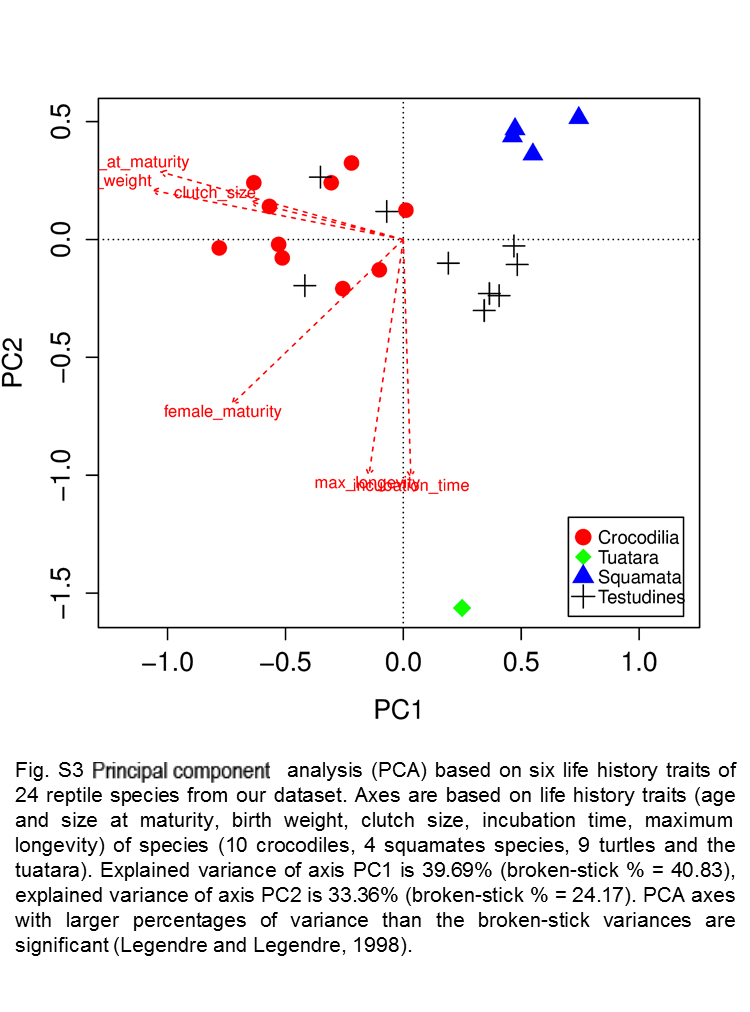


Figure S3 Principal component analysis (PCA) based on six life history traits of 24 reptile species taken from our reptile dataset. Axes are derived from six life history traits (age and size at maturity, birth weight, clutch size, incubation time, maximum longevity). Ten crocodile species (*Alligator mississippiensis*, *Crocodylus acutus*, *Crocodylus johnstoni*, *Crocodylus niloticus*, *Crocodylus palustris*, *Crocodylus porosus*, *Gavialis gangeticus*, *Mecistops cataphractus*, *Paleosuchus trigonatus*, *Tomistoma schlegelii*), four squamates (*Lacerta strigata*, *Macrovipera lebetina*, *Malpolon monspessulanus*, *Natrix natrix*), nine turtles (*Caretta caretta*, *Chelonia mydas*, *Chelydra serpentina*, *Dermochelys coriacea*, *Emys orbicularis*, *Testudo graeca*, *Testudo hermanni*, *Testudo marginata*, *Trachemys scripta*), and the tuatara (*Sphenodon punctatus*) were passed to this analysis. Axis PC1 explains 39.69% of the overall variance (broken-stick % = 40.83%), and PC 2 33.36% (broken-stick % = 24.17). PCA axes with larger percentages of variance than the broken-stick variances are significant (see Legendre L. & Legendre P. (1998): Numerical ecology. Amsterdam, Elsevier)

**Table S1a** Our database on life history traits of 369 reptile species. AW=adult weight (g), BS=birth size (total length, cm), BW=birth weight (g), CS=clutch size, NCPA=number of clutches per year, EW=egg weight (g), FM=female maturity (days), IGT=incubation/gestation time (days), Alt=maximum altitude (m), LONG=maximum longevity (years), SM=size at maturity (total length, cm), SG=squamate group (1=lizards, 2=snakes, 3=amphisbaenians, 0=no squamate), NA=not available.

| Species |  | AW | BS | BW | CS | NCPA | EW | FM | IGT | ALT | LONG | SM | Class | SG | Family | Genus | Infraorder/ Superfamily | References |
| --- | --- | --- | --- | --- | --- | --- | --- | --- | --- | --- | --- | --- | --- | --- | --- | --- | --- | --- |
| Ablepharus | kitaibelii | 0.6 | NA | NA | 3 | 1 | NA | 810 | 65 | 800 | 3.25 | NA | Squamata | 1 | Scincidae | Ablepharus | Scincomorpha | 6,18 |
| Acanthocercus | atricollis | 121.5 | NA | NA | NA | NA | NA | NA | NA | NA | 6 | NA | Squamata | 1 | Agamidae | Acanthocercus | Iguania | 1,18 |
| Acanthodactylus | erythrurus | 8.7 | 6.85 | NA | 4.5 | 1.25 | NA | 905 | 73 | 1900 | NA | NA | Squamata | 1 | Lacertidae | Acanthodactylus | Scincomorpha | 6,18 |
| Acanthophis | antarcticus | 252 | NA | NA | NA | NA | NA | NA | NA | NA | 9.3 | NA | Squamata | 2 | Elapidae | Acanthophis | Colubroidea | 1,19 |
| Agama | impalearis | 100 | NA | NA | NA | NA | NA | NA | NA | NA | 6 | NA | Squamata | 1 | Agamidae | Agama | Iguania | 1,18 |
| Agkistrodon | bilineatus | 51.1 | NA | NA | NA | NA | NA | NA | NA | NA | 24.3 | NA | Squamata | 2 | Viperidae | Agkistrodon | Colubroidea | 1,19 |
| Agkistrodon | contortrix | 258.6 | NA | NA | 7.5 | NA | NA | 730 | 117 | NA | 29.4 | NA | Squamata | 2 | Viperidae | Agkistrodon | Colubroidea | 1,4,19 |
| Agkistrodon | piscivorus | 204 | NA | NA | 8 | NA | NA | 1095 | NA | NA | 24.5 | NA | Squamata | 2 | Viperidae | Agkistrodon | Colubroidea | 1,5,19 |
| Algyroides | moreoticus | 3.2 | 22 | NA | NA | 1 | NA | NA | NA | 1000 | NA | NA | Squamata | 1 | Lacertidae | Algyroides | Scincomorpha | 6,18 |
| Alligator | mississippiensis | 47800 | 23.39 | 44.37 | 42.7 | NA | 76.6 | 4015 | 60 | NA | 73.1 | 180 | Crocodilia | 0 | Alligatoridae | Alligator | NA | 1,3,4 |
| Alligator | sinensis | 14600 | NA | NA | 25.7 | NA | 48.2 | 1642.5 | 70 | 100 | 60.7 | NA | Crocodilia | 0 | Alligatoridae | Alligator | NA | 1,2,3 |
| Alsophylax | pipiens | 1.5 | 2.65 | NA | 1.5 | 1 | NA | 600 | NA | NA | NA | NA | Squamata | 1 | Gekkonidae | Alsophylax | Gekkota | 6,18 |
| Amblyrhynchus | cristatus | 3000 | NA | NA | 3.5 | NA | NA | NA | 95 | NA | 9.2 | NA | Squamata | 1 | Iguanidae | Amblyrhynchus | Iguania | 1,4,18 |
| Ameiva | ameiva | 107.9 | NA | NA | 5.5 | NA | NA | NA | 75 | NA | 4.6 | NA | Squamata | 1 | Teiidae | Ameiva | Scincomorpha | 1,4,18 |
| Amphisbaena | alba | 210 | NA | NA | NA | NA | NA | NA | NA | NA | 15.1 | NA | Squamata | 3 | Amphisbaenidae | Amphisbaena | Amphisbaenia | 1,18 |
| Anguis | fragilis | 14 | 8 | NA | 12.25 | 1 | NA | 1825 | 84 | NA | 44.33 | NA | Squamata | 1 | Anguidae | Anguis | Diploglossa | 1,4,5,6,18 |
| Anniella | pulchra | 4.7 | NA | NA | 2.5 | NA | NA | NA | NA | NA | NA | NA | Squamata | 1 | Anniellidae | Anniella | Diploglossa | 5,18 |
| Anolis | carolinensis | 2.3 | NA | NA | NA | NA | NA | NA | NA | NA | 7.2 | NA | Squamata | 1 | Dactyloidae | Anolis | Iguania | 1,18 |
| Anolis | equestris | 56 | NA | NA | NA | NA | NA | NA | NA | NA | 16.5 | NA | Squamata | 1 | Dactyloidae | Anolis | Iguania | 1,18 |
| Arizona | elegans | 161 | NA | NA | NA | NA | NA | NA | NA | NA | 19.1 | NA | Squamata | 2 | Colubridae | Arizona | Colubroidea | 1,19 |
| Aspidelaps | scutatus | 75.7 | NA | NA | NA | NA | NA | NA | NA | NA | 13.5 | NA | Squamata | 2 | Elapidae | Aspidelaps | Colubroidea | 1,19 |
| Aspidites | melanocephalus | 1362 | NA | NA | NA | NA | NA | NA | NA | NA | 22.6 | NA | Squamata | 2 | Pythonidae | Aspidites | Pythonoidea | 1,19 |
| Aspidoscelis | tigris | 15.5 | NA | NA | NA | NA | NA | NA | NA | NA | 7.8 | NA | Squamata | 1 | Teiidae | Aspidoscelis | Scincomorpha | 1,18 |
| Astrochelys | radiata | 7700 | 3.6 | 30 | 5.1 | 5.5 | NA | NA | 188 | NA | NA | NA | Testudines | 0 | Testudinidae | Astrochelys | Testudinoidea | 1,42,43 |
| Astrochelys | yniphora | 8000 | 4.4 | 25 | 4 | 7 | NA | NA | 217 | NA | NA | NA | Testudines | 0 | Testudinidae | Astrochelys | Testudinoidea | 42,43 |
| Atractaspis | bibronii | 34.3 | NA | NA | NA | NA | NA | NA | NA | NA | 23.9 | NA | Squamata | 2 | Lamprophiidae | Atractaspis | Henophidia | 1,19 |
| Basiliscus | plumifrons | 250 | NA | NA | 10.5 | NA | NA | NA | 60 | NA | 13.7 | NA | Squamata | 1 | Corytophanidae | Basiliscus | Iguania | 1,4 |
| Basiliscus | vittatus | 70 | NA | NA | NA | NA | NA | NA | NA | NA | 9.1 | NA | Squamata | 1 | Corytophanidae | Basiliscus | Iguania | 1,18 |
| Bitis | arietans | 743.3 | NA | NA | 79 | NA | NA | NA | 105 | NA | 15.8 | NA | Squamata | 2 | Viperidae | Bitis | Colubroidea | 1,4,5,19 |
| Bitis | gabonica | 4023.7 | NA | NA | NA | NA | NA | NA | NA | NA | 18 | NA | Squamata | 2 | Viperidae | Bitis | Colubroidea | 1,5,19 |
| Bitis | peringueyi | 8.6 | NA | NA | NA | NA | NA | NA | NA | NA | NA | NA | Squamata | 2 | Viperidae | Bitis | Colubroidea | 5,19 |
| Blanus | cinereus | 6 | NA | NA | 1 | 1 | NA | NA | NA | 1400 | 16 | NA | Squamata | 3 | Blanidae | Blanus | Amphisbaenia | 1,6,18 |
| Boa | constrictor | 3532 | NA | NA | 35 | NA | NA | 1095 | 125 | NA | 40.4 | NA | Squamata | 2 | Boidae | Boa | Booidea | 1,5,19 |
| Bogertophis | subocularis | 213.4 | NA | NA | 5 | NA | NA | 730 | NA | NA | 23.8 | NA | Squamata | 2 | Colubridae | Bogertophis | Colubroidea | 1,19 |
| Boiga | cynodon | 417.3 | NA | NA | NA | NA | NA | NA | NA | NA | 9.6 | NA | Squamata | 2 | Colubridae | Boiga | Colubroidea | 1,19 |
| Boiga | dendrophila | 182 | NA | NA | NA | NA | NA | NA | NA | NA | 17 | NA | Squamata | 2 | Colubridae | Boiga | Colubroidea | 1,19 |
| Boiga | irregularis | 146.9 | NA | NA | NA | NA | NA | NA | NA | NA | 13.1 | NA | Squamata | 2 | Colubridae | Boiga | Colubroidea | 1,19 |
| Bothriechis | schlegelii | 102 | NA | NA | NA | NA | NA | NA | NA | NA | 19.5 | NA | Squamata | 2 | Viperidae | Bothriechis | Colubroidea | 1,19 |
| Brachylophus | fasciatus | 162 | NA | NA | NA | NA | NA | NA | NA | NA | 5.9 | NA | Squamata | 1 | Iguanidae | Brachylophus | Iguania | 1,5,18 |
| Broghammerus | reticulatus | 32000 | 68.58 | NA | 80 | NA | NA | 2008 | 90 | NA | 29.4 | NA | Squamata | 2 | Pythonidae | Broghammerus | Pythonoidea | 1 |
| Caiman | crocodilus | 10900 | NA | NA | 24.4 | NA | 62.9 | 2190 | 84 | 800 | 24.1 | NA | Crocodilia | 0 | Alligatoridae | Caiman | NA | 1,3,25 |
| Caiman | latirostris | 14600 | NA | 30 | 31.4 | NA | 76.2 | 1825 | 70 | 600 | 22 | NA | Crocodilia | 0 | Alligatoridae | Caiman | NA | 1,3,25 |
| Callopistes | maculatus | 77.4 | NA | NA | NA | NA | NA | NA | NA | NA | NA | NA | Squamata | 1 | Teiidae | Callopistes | Scincomorpha | 5,18 |
| Calotes | versicolor | 23.8 | NA | NA | 15 | NA | NA | NA | 46 | NA | 5 | NA | Squamata | 1 | Agamidae | Calotes | Iguania | 1,4,18 |
| Candoia | aspera | 293 | NA | NA | NA | NA | NA | NA | NA | NA | 9.9 | NA | Squamata | 2 | Boidae | Candoia | Booidea | 1,19 |
| Candoia | bibroni | 45.2 | NA | NA | NA | NA | NA | NA | NA | NA | 16.9 | NA | Squamata | 2 | Boidae | Candoia | Booidea | 1,19 |
| Candoia | carinata | 93 | NA | NA | NA | NA | NA | NA | NA | NA | 16.6 | NA | Squamata | 2 | Boidae | Candoia | Booidea | 1,19 |
| Caretta | caretta | 350250 | 5.08 | 16.1 | 97.73 | 2.03 | 34.22 | 3650 | 61 | NA | 37.6 | 88.4 | Testudines | 0 | Cheloniidae | Caretta | Chelonioidea | 1,4,5,14 |
| Causus | rhombeatus | 94.8 | NA | NA | NA | NA | NA | NA | NA | NA | 6.6 | NA | Squamata | 2 | Viperidae | Causus | Colubroidea | 1,19 |
| Cerastes | cerastes | 186.6 | NA | NA | NA | NA | NA | NA | NA | NA | 18 | NA | Squamata | 2 | Viperidae | Cerastes | Colubroidea | 1,19 |
| Chalcides | bedriagai | 7 | 4.33 | NA | 2.5 | 1 | NA | NA | NA | 1100 | 4.5 | NA | Squamata | 1 | Scincidae | Chalcides | Scincomorpha | 6,18 |
| Chalcides | chalcides | 18.8 | 9.4 | NA | 8 | 1 | NA | 730 | NA | 1523.33 | NA | NA | Squamata | 1 | Scincidae | Chalcides | Scincomorpha | 5,6,18 |
| Chalcides | ocellatus | 25 | NA | NA | 8.75 | 1 | NA | NA | 63.67 | 2500 | 13.5 | NA | Squamata | 1 | Scincidae | Chalcides | Scincomorpha | 1,6,18 |
| Chalcides | sexlineatus | 6.85 | 7.89 | 0.45 | 3.75 | 1 | NA | NA | NA | 1949 | NA | NA | Squamata | 1 | Scincidae | Chalcides | Scincomorpha | 10,18 |
| Chalcides | viridanus | 8.5 | 7.37 | 0.96 | 3.25 | 1 | NA | 730 | 89 | 2800 | NA | NA | Squamata | 1 | Scincidae | Chalcides | Scincomorpha | 10 |
| Chamaeleo | chamaeleon | 36.3 | 4.95 | NA | 35 | 1 | NA | NA | 255 | NA | 3.6 | NA | Squamata | 1 | Chamaeleonidae | Chamaeleo | Iguania | 1,6,18 |
| Chamaeleo | dilepis | 13.2 | NA | NA | 30 | NA | NA | NA | 90 | NA | 4 | NA | Squamata | 1 | Chamaeleonidae | Chamaeleo | Iguania | 1,5,18 |
| Chamaeleo | jacksonii | 37.5 | NA | NA | NA | NA | NA | NA | NA | NA | 8.2 | NA | Squamata | 1 | Chamaeleonidae | Chamaeleo | Iguania | 1,18 |
| Chamaeleo | namaquensis | 58.1 | NA | NA | 14 | 3 | NA | NA | 105 | NA | NA | NA | Squamata | 1 | Chamaeleonidae | Chamaelao | Iguania | 1,18 |
| Chamaesaura | macrolepis | 11.6 | NA | NA | 3 | NA | NA | NA | NA | NA | NA | NA | Squamata | 1 | Cordylidae | Chamaesaura | Scincomorpha | 5,18 |
| Charina | bottae | 39.8 | 19 | NA | NA | NA | NA | NA | NA | NA | 26.5 | NA | Squamata | 2 | Boidae | Charina | Booidea | 1,5,19 |
| Chelonia | mydas | 205400 | 5.01 | 26.35 | 114.8 | 3.33 | 47.5 | 6479 | 62.5 | NA | 50.96 | 99 | Testudines | 0 | Cheloniidae | Chelonia | Chelonioidea | 1,4,5,14 |
| Chelonoidis | carbonaria | 3961 | 4.2 | 30 | 8.5 | NA | NA | NA | 150 | NA | 49 | NA | Testudines | 0 | Testudinidae | Chelonoidis | Testudinoidea | 1,20,43 |
| Chelonoidis | chilensis | 2640 | 5.5 | NA | 4 | 3 | NA | 4380 | 365 | 1000 | NA | NA | Testudines | 0 | Testudinidae | Chelonoidis | Testudinoidea | 30,43 |
| Chelonoidis | denticulata | 9800 | 5.35 | 40 | 6 | NA | NA | NA | 135 | NA | NA | NA | Testudines | 0 | Testudinidae | Chelonoidis | Testudinoidea | 1,39,43 |
| Chelonoidis | nigra | 184000 | 6 | 50 | 6 | NA | NA | NA | 152.5 | NA | 177.2 | NA | Testudines | 0 | Testudinidae | Chelonoidis | Testudinoidea | 1,40,43 |
| Chelydra | serpentina | 29562.5 | 2.75 | 9 | 25 | 1 | 10 | 3285 | 100 | NA | 47 | 141.5 | Testudines | 0 | Chelydridae | Chelydra | NA | 1,5 |
| Chersina | angulata | 760 | 3.45 | 12 | NA | 1 | NA | NA | 180 | 900 | NA | NA | Testudines | 0 | Testudinidae | Chersina | Testudinoidea | 28,30,49,43 |
| Chitra | indica | 112500 | 4.1 | NA | 118 | NA | NA | NA | NA | NA | NA | NA | Testudines | 0 | Trionychidae | Chitra | Trionychoidea | 5 |
| Chlamydosaurus | kingii | 635 | NA | NA | NA | NA | NA | NA | NA | NA | 9.9 | NA | Squamata | 1 | Agamidae | Chlamydosaurus | Iguania | 1,18 |
| Chondrodactylus | angulifer | 15.7 | 6.7 | NA | 1.5 | 1 | NA | NA | 72 | NA | 10.8 | NA | Squamata | 1 | Gekkonidae | Chondrodactylus | Gekkota | 1,15,17,18 |
| Christinus | marmoratus | 3.7 | NA | NA | NA | NA | NA | NA | NA | NA | 12.8 | NA | Squamata | 1 | Gekkonidae | Christinus | Gekkota | 1,18 |
| Chrysemys | picta | 371.8 | 2.54 | NA | 11 | 4 | NA | 2750 | 74 | NA | 40.5 | 10.5 | Testudines | 0 | Emydidae | Chrysemys | Testudinoidea | 1,4 |
| Clelia | clelia | 2050 | NA | NA | NA | NA | NA | NA | NA | NA | 11.5 | NA | Squamata | 2 | Colubridae | Clelia | Colubroidea | 1,19 |
| Coluber | constrictor | 121 | NA | NA | NA | NA | NA | NA | NA | NA | 10 | NA | Squamata | 2 | Colubridae | Coluber | Colubroidea | 1,19 |
| Conolophus | pallidus | 4200 | NA | NA | NA | NA | NA | NA | NA | NA | 17.1 | NA | Squamata | 1 | Iguanidae | Conolophus | Iguania | 1,18 |
| Conolophus | subcristatus | 7000 | NA | NA | NA | NA | NA | NA | NA | NA | 15 | NA | Squamata | 1 | Iguanidae | Conolophus | Iguania | 1,18 |
| Corallus | caninus | 586.5 | NA | NA | NA | NA | NA | NA | NA | NA | 18.6 | NA | Squamata | 2 | Boidae | Corallus | Booidea | 1,19 |
| Corallus | hortulanus | 207 | NA | NA | NA | NA | NA | NA | NA | NA | 15 | NA | Squamata | 2 | Boidae | Corallus | Booidea | 1,19 |
| Cordylus | cataphractus | 5400 | NA | NA | 1.5 | NA | NA | NA | NA | NA | 20.2 | NA | Squamata | 1 | Cordylidae | Cordylus | Scincomorpha | 1,4 |
| Cordylus | cordylus | 17.3 | NA | NA | NA | NA | NA | NA | NA | NA | 15.6 | NA | Squamata | 1 | Cordylidae | Cordylus | Scincomorpha | 1,18 |
| Cordylus | giganteus | 131.2 | NA | NA | NA | NA | NA | NA | NA | NA | 24.9 | NA | Squamata | 1 | Cordylidae | Cordylus | Scincomorpha | 1,18 |
| Coronella | austriaca | 140 | 20.5 | 3 | 13.17 | 0.83 | NA | 1460.5 | 140 | 2600 | NA | 45 | Squamata | 2 | Colubridae | Coronella | Colubroidea | 5,9 |
| Coronella | girondica | 50.22 | 15.02 | NA | 6.86 | 1 | 3.52 | 1460 | 47.5 | 3200 | 14 | NA | Squamata | 2 | Colubridae | Coronella | Colubroidea | 9,19 |
| Corucia | zebrata | 1013.7 | 30.48 | NA | 1.5 | 0.5 | NA | 1095 | NA | NA | 24.3 | NA | Squamata | 1 | Scincidae | Corucia | Scincomorpha | 1,4,5,18 |
| Corytophanes | cristatus | 43.4 | NA | NA | NA | NA | NA | NA | NA | NA | 7.1 | NA | Squamata | 1 | Corytophanidae | Corytophanes | Iguania | 1,18 |
| Crocodylus | acutus | 76700 | 25 | NA | 35.6 | NA | 112.8 | 3285 | 66.5 | NA | 32.9 | 210 | Crocodilia | 0 | Crocodylidae | Crocodylus | NA | 1,2,3 |
| Crocodylus | intermedius | 107900 | NA | NA | 39.8 | NA | 110.4 | NA | 70 | NA | 21.8 | NA | Crocodilia | 0 | Crocodylidae | Crocodylus | NA | 1,2,3 |
| Crocodylus | johnstoni | 19500 | NA | 42 | 12.4 | NA | 68 | 4562.5 | 80 | NA | 20 | 118.5 | Crocodilia | 0 | Crocodylidae | Crocodylus | NA | 1,3 |
| Crocodylus | mindorensis | 36900 | NA | NA | 18.3 | NA | 73.6 | NA | 85 | NA | NA | NA | Crocodilia | 0 | Crocodylidae | Crocodylus | NA | 2,3 |
| Crocodylus | moreletii | 31700 | NA | NA | 30.2 | NA | 79.5 | 2555 | 75 | NA | 25.4 | NA | Crocodilia | 0 | Crocodylidae | Crocodylus | NA | 1,2,3 |
| Crocodylus | niloticus | 94200 | NA | NA | 47.6 | NA | 107.1 | 4927.5 | 90 | NA | 43.8 | 250 | Crocodilia | 0 | Crocodylidae | Crocodylus | NA | 1,2,3 |
| Crocodylus | novaeguineae | 39900 | 29 | NA | 29.4 | NA | 85.15 | NA | 87 | 600 | 24.6 | 205 | Crocodilia | 0 | Crocodylidae | Crocodylus | NA | 1,2,3,25 |
| Crocodylus | palustris | 42700 | 25 | NA | 41.7 | 1 | 99.5 | 2190 | 72.5 | 700 | 31.5 | 222.5 | Crocodilia | 0 | Crocodylidae | Crocodylus | NA | 1,2,3,5 |
| Crocodylus | porosus | 78700 | NA | 72 | 58.9 | NA | 117.35 | 3650 | 87.38 | NA | 41.7 | 285 | Crocodilia | 0 | Crocodylidae | Crocodylus | NA | 1,2,3,4 |
| Crocodylus | rhombifer | 57500 | NA | NA | 25.4 | NA | 112 | NA | 64 | NA | 38.2 | NA | Crocodilia | 0 | Crocodylidae | Crocodylus | NA | 1,2,3 |
| Crocodylus | siamensis | 42500 | NA | NA | 28.4 | NA | 106.9 | 3650 | 73.75 | NA | 21.9 | NA | Crocodilia | 0 | Crocodylidae | Crocodylus | NA | 1,2,3 |
| Crotalus | atrox | 1000 | NA | NA | 14 | NA | NA | 1095 | NA | NA | 27 | NA | Squamata | 2 | Viperidae | Crotalus | Colubroidea | 1,19 |
| Crotalus | cerastes | 104.79 | NA | NA | 11.5 | NA | NA | NA | NA | NA | 27.3 | NA | Squamata | 2 | Viperidae | Crotalus | Colubroidea | 1,4,19 |
| Crotalus | durissus | 2115.38 | NA | 14 | 20.5 | NA | NA | 1095 | 120 | NA | 19.8 | NA | Squamata | 2 | Viperidae | Crotalus | Colubroidea | 1,5,19 |
| Crotalus | horridus | 581.9 | NA | NA | 9 | NA | NA | 2555 | 135 | NA | 30.2 | NA | Squamata | 2 | Viperidae | Crotalus | Colubroidea | 1,19 |
| Crotalus | molossus | 438.4 | NA | NA | NA | NA | NA | NA | NA | NA | 20.7 | NA | Squamata | 2 | Viperidae | Crotalus | Colubroidea | 1,19 |
| Crotalus | pricei | 62.6 | NA | NA | NA | NA | NA | NA | NA | NA | 15.7 | NA | Squamata | 2 | Viperidae | Crotalus | Colubroidea | 1,19 |
| Crotalus | ruber | 285 | NA | NA | NA | NA | NA | NA | NA | NA | 19.2 | NA | Squamata | 2 | Viperidae | Crotalus | Colubroidea | 1,19 |
| Crotalus | viridis | 318.3 | NA | NA | NA | NA | NA | NA | NA | NA | 24.1 | NA | Squamata | 2 | Viperidae | Crotalus | Colubroidea | 1,19 |
| Ctenosaura | bakeri | 399.4 | NA | NA | NA | NA | NA | NA | NA | NA | 13.8 | NA | Squamata | 1 | Iguanidae | Ctenosaura | Iguania | 1,18 |
| Ctenosaura | hemilopha | 2800 | NA | NA | NA | NA | NA | NA | NA | NA | 9.6 | NA | Squamata | 1 | Iguanidae | Ctenosaura | Iguania | 1,18 |
| Ctenosaura | pectinata | 961.4 | NA | NA | NA | NA | NA | NA | NA | NA | 8.2 | NA | Squamata | 1 | Iguanidae | Ctenosaura | Iguania | 1,18 |
| Ctenosaura | similis | 1034 | NA | NA | NA | NA | NA | NA | NA | NA | 22.4 | NA | Squamata | 1 | Iguanidae | Ctenosaura | Iguania | 1,18 |
| Cyclura | cornuta | 4134.5 | NA | NA | NA | NA | NA | NA | NA | NA | 22.9 | NA | Squamata | 1 | Iguanidae | Cyclura | Iguania | 1,18 |
| Cyclura | cychlura | 10380 | NA | NA | NA | NA | NA | NA | NA | NA | 23.4 | NA | Squamata | 1 | Iguanidae | Cyclura | Iguania | 1,18 |
| Cyclura | nubila | 4100 | NA | NA | NA | NA | NA | NA | NA | NA | 47.6 | NA | Squamata | 1 | Iguanidae | Cyclura | Iguania | 1,18 |
| Cyclura | rileyi | 683 | NA | NA | NA | NA | NA | NA | NA | NA | NA | NA | Squamata | 1 | Iguanidae | Cyclura | Iguania | 1,18 |
| Cyrtopodion | fedtschenkoi | 9 | NA | NA | 1.2 | 1 | NA | NA | NA | NA | NA | NA | Squamata | 1 | Gekkonidae | Cyrtopodion | Gekkota | 15,18 |
| Cyrtopodion | kotschyi | 2 | 1.9 | 0.4 | 1.5 | 1 | NA | NA | 103 | 800 | 9 | NA | Squamata | 1 | Gekkonidae | Mediodactylus | Gekkota | 6,18 |
| Cyrtopodion | russowii | 3 | 35 | NA | 1.5 | 1.5 | NA | 660 | 51 | 2000 | NA | NA | Squamata | 1 | Gekkonidae | Mediodactylus | Gekkota | 6,15 |
| Daboia | russelii | 675 | NA | NA | NA | NA | NA | NA | NA | NA | 15 | NA | Squamata | 2 | Viperidae | Daboia | Colubroidea | 1,5,19 |
| Darevskia | rudis | 12.95 | 5.25 | NA | 5 | 1 | NA | NA | NA | 2100 | NA | NA | Squamata | 1 | Lacertidae | Darevskia | Scincomorpha | 7 |
| Dasypeltis | scabra | 15.7 | NA | NA | NA | NA | NA | NA | NA | NA | 22.1 | NA | Squamata | 2 | Colubridae | Dasypeltis | Colubroidea | 1,19 |
| Dendroaspis | angusticeps | 282.4 | NA | NA | NA | NA | NA | NA | NA | NA | 18.8 | NA | Squamata | 2 | Elapidae | Dendroaspis | Colubroidea | 1,19 |
| Dendroaspis | polylepis | 651.7 | NA | NA | NA | NA | NA | NA | NA | NA | 26.2 | NA | Squamata | 2 | Elapidae | Dendroaspis | Colubroidea | 1,19 |
| Dermochelys | coriacea | 428714.29 | 5.88 | 43.5 | 96.25 | 4.13 | 86.8 | 4106.5 | 64.33 | NA | 24.13 | 139 | Testudines | 0 | Dermochelyidae | Dermochelys | Chelonioidea | 1,5,14 |
| Dinodon | rufozonatum | 160.9 | NA | NA | NA | NA | NA | NA | NA | NA | 13.7 | NA | Squamata | 2 | Colubridae | Dinodon | Colubroidea | 1,19 |
| Dipsochelys | dussumieri | 167000 | 7.25 | 54 | 9 | 1.5 | NA | NA | 98 | NA | 152 | 30 | Testudines | 0 | Testudinidae | Dipsochelys | Testudinoidea | 1,30,43 |
| Dipsosaurus | dorsalis | 64.25 | NA | NA | NA | NA | NA | NA | NA | NA | 14.6 | NA | Squamata | 1 | Iguanidae | Dipsosaurus | Iguania | 1,18 |
| Dolichophis | caspius | 230 | 29.6 | NA | 9.83 | 1 | NA | NA | NA | 1600 | 9 | 67.5 | Squamata | 2 | Colubridae | Dolichophis | Colubroidea | 9 |
| Dracaena | guianensis | 1450 | NA | NA | 2 | NA | NA | NA | NA | NA | 9.3 | NA | Squamata | 1 | Teiidae | Dracaena | Scincomorpha | 1,4,5,18 |
| Draco | volans | 5.4 | NA | NA | 4.5 | NA | NA | NA | 32 | NA | NA | NA | Squamata | 1 | Agamidae | Draco | Iguania | 4,18 |
| Drymarchon | corais | 829 | NA | NA | 6 | NA | NA | NA | NA | NA | 25.9 | NA | Squamata | 2 | Colubridae | Drymarchon | Colubroidea | 1,19 |
| Echis | carinatus | 146.05 | NA | NA | NA | NA | NA | NA | NA | NA | 23.8 | NA | Squamata | 2 | Viperidae | Echis | Colubroidea | 1,19 |
| Echis | coloratus | 120.25 | NA | NA | NA | NA | NA | NA | NA | NA | 28.3 | NA | Squamata | 2 | Viperidae | Echis | Colubroidea | 1,19 |
| Egernia | cunninghami | 240 | NA | NA | NA | NA | NA | NA | NA | NA | 26.5 | NA | Squamata | 1 | Scincidae | Egernia | Scincomorpha | 1,18 |
| Egernia | kingii | 291 | NA | NA | NA | NA | NA | NA | NA | NA | NA | NA | Squamata | 1 | Scincidae | Egernia | Scincomorpha | 5,18 |
| Egernia | stokesii | 248 | NA | NA | NA | NA | NA | NA | NA | NA | 18.2 | NA | Squamata | 1 | Scincidae | Egernia | Scincomorpha | 1,18 |
| Eirenis | modestus | 15.5 | 11 | NA | 5.5 | 1 | NA | NA | NA | 2000 | NA | NA | Squamata | 2 | Colubridae | Eirenis | Colubroidea | 9,19 |
| Elaphe | quadrivirgata | 188.7 | NA | NA | NA | NA | NA | NA | NA | NA | 16.8 | NA | Squamata | 2 | Colubridae | Elaphe | Colubroidea | 1,19 |
| Elaphe | quatuorlineata | 400 | 27.14 | NA | 9.7 | 1 | 17 | 1004 | 59 | 2500 | NA | 128 | Squamata | 2 | Colubridae | Elaphe | Colubroidea | 9,19 |
| Elgaria | kingii | 10 | NA | NA | NA | NA | NA | NA | NA | NA | 12.3 | NA | Squamata | 1 | Anguidae | Elgaria | Diploglossa | 1,18 |
| Elgaria | multicarinata | 31.7 | NA | NA | NA | NA | NA | NA | NA | NA | 9.8 | NA | Squamata | 1 | Anguidae | Elgaria | Diploglossa | 1,18 |
| Emys | orbicularis | 1177.79 | 2.25 | 5.12 | 9.44 | 1.99 | 7.25 | 3262.38 | 91.45 | 1400 | 41.25 | 13.64 | Testudines | 0 | Emydidae | Emys | Testudinoidea | 1,5,13 |
| Epicrates | cenchria | 830 | NA | NA | NA | NA | NA | NA | NA | NA | 31 | NA | Squamata | 2 | Boidae | Epicrates | Booidea | 1,19 |
| Epicrates | inornatus | 1058.9 | NA | NA | NA | NA | NA | NA | NA | NA | 23.9 | NA | Squamata | 2 | Boidae | Epicrates | Booidea | 1,19 |
| Eremias | arguta | 14.65 | 4.8 | NA | 6.5 | 1.25 | 0.36 | 525 | 75 | 2000 | NA | 5.5 | Squamata | 1 | Lacertidae | Eremias | Scincomorpha | 5,6,18 |
| Eretmochelys | imbricata | 48500 | 4.45 | 13.43 | 137.6 | 2.28 | NA | 1338.33 | 60.75 | NA | NA | 66.5 | Testudines | 0 | Cheloniidae | Eretmochelys | Chelonioidea | 1,5,14 |
| Eristicophis | macmahoni | 125 | NA | NA | NA | NA | NA | NA | NA | NA | 14.6 | NA | Squamata | 2 | Viperidae | Eristicophis | Colubroidea | 1,19 |
| Erpeton | tentaculatum | 92.9 | NA | NA | NA | NA | NA | NA | NA | NA | 13.6 | NA | Squamata | 2 | Colubridae | Erpeton | Colubroidea | 1,19 |
| Eryx | jaculus | 134.5 | 16.75 | 8 | 11.33 | 1 | NA | 730 | 107 | 1700 | 24.7 | NA | Squamata | 2 | Boidae | Eryx | Booidea | 1,9,19 |
| Eryx | johnii | 531 | NA | NA | NA | NA | NA | NA | NA | NA | 24.4 | NA | Squamata | 2 | Boidae | Eryx | Booidea | 1,19 |
| Eublepharis | macularius | 59.7 | 8.18 | 3.6 | 1.75 | 4 | NA | 400 | 54.25 | NA | 24.25 | NA | Squamata | 1 | Eublepharidae | Eublepharis | Gekkota | 1,4,5,15,16,18 |
| Eumeces | algeriensis | 243.8 | NA | NA | NA | NA | NA | NA | NA | NA | 22.8 | NA | Squamata | 1 | Scincidae | Eumeces | Scincomorpha | 1,18 |
| Eumeces | schneideri | 85.4 | 6.5 | NA | 10.25 | 1 | NA | NA | NA | 1800 | NA | NA | Squamata | 1 | Scincidae | Eumeces | Scincomorpha | 6,18 |
| Eunectes | murinus | 102950 | 70 | NA | 25 | NA | NA | NA | 231 | NA | 31.8 | NA | Squamata | 2 | Boidae | Eunectes | Booidea | 1,4,5,19 |
| Gallotia | atlantica | 12.8 | 7.56 | 0.66 | 2.68 | 1.95 | 0.55 | 730 | 68.25 | 580 | 15 | NA | Squamata | 1 | Lacertidae | Gallotia | Scincomorpha | 10,18 |
| Gallotia | galloti | 72.75 | 12.33 | 1.6 | 5.24 | 1.5 | 1.82 | 730 | 65.86 | 2000 | 7.03 | NA | Squamata | 1 | Lacertidae | Gallotia | Scincomorpha | 1,5,10,18 |
| Gallotia | simonyi | 252.31 | 3.63 | NA | 10.8 | 1.5 | 5.4 | 913 | 64.5 | 500 | 5.4 | NA | Squamata | 1 | Lacertidae | Gallotia | Scincomorpha | 1,5,10,18 |
| Gallotia | stehlini | 326.5 | 13.23 | 2.1 | 10.33 | 1.5 | NA | 1346.5 | 80 | 1950 | 13 | NA | Squamata | 1 | Lacertidae | Gallotia | Scincomorpha | 10,18 |
| Gavialis | gangeticus | 147000 | 35.56 | NA | 37.2 | 1 | 139.5 | 4380 | 75 | 500 | 28.8 | 300 | Crocodilia | 0 | Gavialidae | Gavialis | NA | 1,2,3,4 |
| Gehyra | mutilata | 1.7 | NA | NA | 1 | 5 | NA | NA | 60 | NA | NA | NA | Squamata | 1 | Gekkonidae | Gehyra | Gekkota | 15,18 |
| Gehyra | oceanica | 7.9 | NA | NA | NA | NA | NA | NA | NA | NA | 10.25 | NA | Squamata | 1 | Gekkonidae | Gehyra | Gekkota | 1,15,18 |
| Gehyra | variegata | 3 | 4.8 | 0.39 | 1 | NA | NA | NA | 60 | NA | 4 | NA | Squamata | 1 | Gekkonidae | Gehyra | Gekkota | 15,18 |
| Gekko | gecko | 63.2 | 9.5 | NA | 2.5 | NA | NA | NA | 125.5 | NA | 23.5 | NA | Squamata | 1 | Gekkonidae | Gekko | Gekkota | 1,4,5,18 |
| Geochelone | elegans | 2700 | 3.75 | NA | 6 | 3 | NA | 2372.5 | 198 | NA | 24.3 | NA | Testudines | 0 | Testudinidae | Geochelone | Testudinoidea | 1 |
| Geochelone | sulcata | 29700 | 4.9 | 50 | 17 | NA | NA | NA | 166 | NA | 54.3 | NA | Testudines | 0 | Testudinidae | Geochelone | Testudinoidea | 1,41,43 |
| Gerrhosaurus | major | 284 | NA | NA | NA | NA | NA | NA | NA | NA | 24 | NA | Squamata | 1 | Gerrhosauridae | Gerrhosaurus | Scincomorpha | 1,5,18 |
| Gloydius | blomhoffii | 63.7 | NA | NA | NA | NA | NA | NA | NA | NA | 13.8 | NA | Squamata | 2 | Viperidae | Gloydius | Colubroidea | 1,19 |
| Gonatodes | albogularis | 2.2 | 3.65 | NA | 1 | 7 | 0.26 | NA | 77.5 | NA | 5.6 | NA | Squamata | 1 | Sphaerodactylidae | Gonatodes | Gekkota | 1,15,16,18 |
| Gonatodes | humeralis | 1.2 | 4 | 0.14 | 1 | 7 | 0.23 | NA | 69 | NA | NA | NA | Squamata | 1 | Sphaerodactylidae | Gonatodes | Gekkota | 15,18 |
| Gopherus | agassizii | 1147 | 4.2 | 25 | 8 | 1.5 | NA | 5500 | 104 | 1400 | 62.8 | 22.91 | Testudines | 0 | Testudinidae | Gopherus | Testudinoidea | 1,30,46 |
| Gopherus | berlandieri | 741 | 4.5 | 21 | 2.5 | 1.5 | NA | 1460 | 88 | 884 | 30.7 | 12.5 | Testudines | 0 | Testudinidae | Gopherus | Testudinoidea | 1,30,47 |
| Gopherus | polyphemus | 4400 | 4.19 | NA | 8 | 1 | NA | 3650 | 100 | NA | NA | 22.82 | Testudines | 0 | Testudinidae | Gopherus | Testudinoidea | 148 |
| Heloderma | horridum | 1088.3 | NA | NA | 9 | NA | NA | NA | 30 | NA | 34.4 | NA | Squamata | 1 | Helodermatidae | Heloderma | Platynota | 1,5,18 |
| Heloderma | suspectum | 551.3 | NA | NA | 9 | NA | NA | NA | 30 | NA | 28.9 | NA | Squamata | 1 | Helodermatidae | Heloderma | Platynota | 1,5,18 |
| Hemidactylus | turcicus | 2.8 | 2.8 | NA | 2.5 | 2.88 | NA | 650 | 58.75 | NA | 7.9 | NA | Squamata | 1 | Gekkonidae | Hemidactylus | Gekkota | 1,6,18 |
| Hemitheconyx | caudicinctus | 44 | 7 | NA | 2 | 3 | NA | NA | 68.5 | NA | 13.1 | NA | Squamata | 1 | Eublepharidae | Hemitheconyx | Gekkota | 1,15,16,17,18 |
| Hemorrhois | hippocrepis | 700 | 25.9 | NA | 11.5 | 1 | 10.95 | 2920 | 56 | 2100 | NA | NA | Squamata | 2 | Colubridae | Hemorrhois | Colubroidea | 9 |
| Hemorrhois | ravergieri | 157.75 | 2.05 | NA | 11.5 | 1 | NA | 913 | 65 | 3300 | 13.8 | 50.5 | Squamata | 2 | Colubridae | Hemorrhois | Colubroidea | 1,9 |
| Heterodon | platirhinos | 280 | NA | NA | NA | NA | NA | 730 | NA | NA | 9.1 | NA | Squamata | 2 | Colubridae | Heterodon | Colubroidea | 1,19 |
| Heteronotia | binoei | 1.8 | 3.75 | 0.2 | 1.5 | 2 | NA | NA | 68 | NA | 15.3 | NA | Squamata | 1 | Gekkonidae | Heteronotia | Gekkota | 1,15,18 |
| Hierophis | gemonensis | 95 | NA | NA | 6.5 | 1 | NA | NA | NA | 1400 | NA | NA | Squamata | 2 | Colubridae | Hierophis | Colubroidea | 9 |
| Hierophis | viridiflavus | 270 | 22.75 | NA | 15 | 1 | 4.8 | 1460 | 51 | 2000 | NA | NA | Squamata | 2 | Colubridae | Hierophis | Colubroidea | 9 |
| Homopus | areolatus | 230 | 3.1 | 6 | 3 | 2 | NA | NA | 150 | 1300 | 28.6 | NA | Testudines | 0 | Testudinidae | Homopus | Testudinoidea | 1,5,30,35 |
| Homopus | boulengeri | 177 | NA | NA | 1 | NA | NA | NA | NA | NA | NA | NA | Testudines | 0 | Testudinidae | Homopus | Testudinoidea | 35 |
| Homopus | femoralis | 393 | 2.75 | NA | 3 | NA | NA | NA | NA | NA | NA | NA | Testudines | 0 | Testudinidae | Homopus | Testudinoidea | 5,33 |
| Homopus | signatus | 151 | 3.14 | 8.5 | 1.5 | 3 | NA | NA | 109 | 1000 | NA | NA | Testudines | 0 | Testudinidae | Homopus | Testudinoidea | 5,28,34 |
| Hoplodactylus | duvaucelii | 118 | NA | NA | NA | NA | NA | NA | NA | NA | 22.5 | NA | Squamata | 1 | Gekkonidae | Hoplodactylus | Gekkota | 1,18 |
| Hoplodactylus | maculatus | 8.1 | NA | NA | NA | NA | NA | NA | NA | NA | 37 | NA | Squamata | 1 | Gekkonidae | Hoplodactylus | Gekkota | 1,15,18 |
| Hoplodactylus | pacificus | 19.6 | NA | NA | NA | NA | NA | NA | NA | NA | 13 | NA | Squamata | 1 | Gekkonidae | Hoplodactylus | Gekkota | 1,18 |
| Iguana | iguana | 5765 | NA | NA | 40 | NA | NA | 1640 | 90 | NA | 27.4 | NA | Squamata | 1 | Iguanidae | Iguana | Iguania | 1,4,5 |
| Indotestudo | elongata | 2700 | 4 | 22 | 4 | NA | NA | NA | 100 | NA | NA | NA | Testudines | 0 | Testudinidae | Indotestudo | Testudinoidea | 1,28,30 |
| Indotestudo | forstenii | 1004 | 5.5 | NA | 4 | NA | NA | NA | 140 | 1000 | 32.9 | NA | Testudines | 0 | Testudinidae | Indotestudo | Testudinoidea | 1,38 |
| Kinixys | belliana | 752 | 4.6 | 20 | 3 | NA | NA | NA | NA | NA | 26.5 | NA | Testudines | 0 | Testudinidae | Kinixys | Testudinoidea | 1,29,43 |
| Kinixys | erosa | 1200 | 4 | 30 | 2.5 | NA | NA | NA | 300 | NA | 24.8 | NA | Testudines | 0 | Testudinidae | Kinixys | Testudinoidea | 1,21,43 |
| Kinixys | homeana | 768.9 | 4.45 | NA | NA | NA | NA | NA | 122 | NA | NA | NA | Testudines | 0 | Testudinidae | Kinixys | Testudinoidea | 22 |
| Lacerta | agilis | 8.3 | 5.89 | 0.47 | 11.5 | 1.25 | NA | 365 | 50 | NA | 11 | NA | Squamata | 1 | Lacertidae | Lacerta | Scincomorpha | 7,18 |
| Lacerta | schreiberi | 21.2 | 8 | NA | 17 | 1 | NA | NA | NA | 1333.33 | NA | NA | Squamata | 1 | Lacertidae | Lacerta | Scincomorpha | 7,18 |
| Lacerta | strigata | 20.6 | 8.25 | 860 | 10.17 | 1.25 | 0.97 | 645 | 50 | 2000 | 7 | 7.7 | Squamata | 1 | Lacertidae | Lacerta | Scincomorpha | 7,18 |
| Lacerta | trilineata | 79.4 | 10.53 | 1.59 | 12.86 | 1.5 | 1.3 | NA | 95.71 | 1250 | NA | 10.65 | Squamata | 1 | Lacertidae | Lacerta | Scincomorpha | 7,18 |
| Lacerta | viridis | 36.98 | NA | NA | 14.83 | 1.5 | 0.84 | NA | 65.29 | 2020 | 10 | NA | Squamata | 1 | Lacertidae | Lacerta | Scincomorpha | 1,7,18 |
| Lampropeltis | getula | 258 | NA | NA | NA | NA | NA | 1095 | NA | NA | 33.3 | NA | Squamata | 2 | Colubridae | Lampropeltis | Colubroidea | 1,19 |
| Laudakia | stellio | 53.4 | NA | NA | 10 | 2.5 | NA | 540 | 87 | 1600 | 6 | NA | Squamata | 1 | Agamidae | Laudakia | Iguania | 1,6,18 |
| Leiocephalus | carinatus | 30 | NA | NA | NA | NA | NA | NA | NA | NA | 10.8 | NA | Squamata | 1 | Leiocephalidae | Leiocephalus | Iguania | 1,18 |
| Leiolepis | belliana | 40 | NA | NA | NA | NA | NA | NA | NA | NA | 5.6 | NA | Squamata | 1 | Agamidae | Leiolepis | Iguania | 1,18 |
| Lepidochelys | kempii | 5895.79 | NA | 16.4 | 107.5 | 1.47 | 30 | 3224.33 | 56 | NA | NA | 56.8 | Testudines | 0 | Cheloniidae | Lepidochelys | Chelonioidea | 1,5,14 |
| Lepidophyma | flavimaculatum | 11.3 | NA | NA | NA | NA | NA | NA | NA | NA | 11 | NA | Squamata | 1 | Xantusiidae | Lepidophyma | Scincomorpha | 1,18 |
| Leptophis | mexicanus | 43.1 | NA | NA | NA | NA | NA | NA | NA | NA | 7.7 | NA | Squamata | 2 | Colubridae | Leptophis | Colubroidea | 1,19 |
| Liasis | fuscus | 953 | NA | NA | NA | NA | NA | NA | NA | NA | 26.8 | NA | Squamata | 2 | Pythonidae | Liasis | Pythonoidea | 1,19 |
| Lichanura | trivirgata | 450 | NA | NA | 7 | NA | NA | NA | 130 | NA | 31 | NA | Squamata | 2 | Boidae | Lichanura | Booidea | 1 |
| Macrochelys | temminckii | 84500 | 4.03 | NA | 30 | NA | NA | 4015 | 105 | NA | 75.15 | NA | Testudines | 0 | Chelydridae | Macrochelys | NA | 1,4 |
| Macroprotodon | cucullatus | 16.6 | NA | NA | 6.33 | 1 | NA | NA | NA | 3000 | 5.9 | NA | Squamata | 2 | Colubridae | Macroprotodon | Colubroidea | 1,11,19 |
| Macrovipera | lebetina | 1037.83 | 20.04 | 16.46 | 21.67 | 1 | 17 | 1350 | 40 | 2500 | 15.15 | 72.75 | Squamata | 2 | Viperidae | Macrovipera | Colubroidea | 1,12,19 |
| Malaclemys | terrapin | 720 | 2.95 | NA | 9 | 3.5 | NA | 2190 | 84 | NA | NA | 12 | Testudines | 0 | Emydidae | Malaclemys | Testudinoidea | 1 |
| Malacochersus | tornieri | 470 | 3.85 | NA | 1.5 | NA | NA | NA | 140 | 1800 | 25.9 | NA | Testudines | 0 | Testudinidae | Malacochersus | Testudinoidea | 1,44 |
| Malpolon | monspessulanus | 649.92 | 27.26 | 9.39 | 11.95 | 1.25 | 15.62 | 1642.5 | 48.5 | 2000 | 13.5 | 90.41 | Squamata | 2 | Lamprophiidae | Malpolon | Colubroidea | 11,19 |
| Manouria | emys | 13400 | 5.8 | NA | 24.67 | 3 | NA | NA | 71 | NA | 19.9 | NA | Testudines | 0 | Testudinidae | Manouria | Testudinoidea | 1,36,37 |
| Mecistops | cataphractus | 50500 | NA | NA | 19.5 | NA | 146 | 4562.5 | 75 | NA | 56.1 | 225 | Crocodilia | 0 | Crocodylidae | Crocodylus | NA | 1,2,3 |
| Melanosuchus | niger | 82000 | NA | NA | 39.3 | NA | 143.6 | NA | 75 | NA | 15.3 | NA | Crocodilia | 0 | Alligatoridae | Melanosuchus | NA | 1,2,3,4 |
| Moloch | horridus | 31.4 | NA | NA | 6.5 | NA | NA | NA | 111 | NA | NA | NA | Squamata | 1 | Agamidae | Moloch | Iguania | 4,18 |
| Montivipera | xanthina | 383.65 | 19.58 | 7.62 | 7.85 | 1 | NA | 730 | 84 | 2500 | NA | NA | Squamata | 2 | Viperidae | Daboia | Colubroidea | 12,19 |
| Morelia | spilota | 2120.15 | NA | NA | NA | NA | NA | NA | NA | NA | 19.6 | NA | Squamata | 2 | Pythonidae | Morelia | Pythonoidea | 1,19 |
| Morelia | viridis | 563.2 | NA | NA | 18 | NA | NA | NA | 50 | NA | 20.3 | NA | Squamata | 2 | Pythonidae | Morelia | Pythonoidea | 1,4,19 |
| Naja | atra | 311.1 | NA | NA | NA | NA | NA | NA | NA | NA | 11.7 | NA | Squamata | 2 | Elapidae | Naja | Colubroidea | 1,19 |
| Naja | mossambica | 171.3 | NA | NA | NA | NA | NA | NA | NA | NA | 16.7 | NA | Squamata | 2 | Elapidae | Naja | Colubroidea | 1,19 |
| Naja | naja | 1497.5 | 22.86 | NA | 16 | NA | NA | NA | 70 | NA | 32.33 | NA | Squamata | 2 | Elapidae | Naja | Colubroidea | 1,4,5,19 |
| Naja | nigricollis | 256.7 | NA | NA | NA | NA | NA | NA | NA | NA | 23.2 | NA | Squamata | 2 | Elapidae | Naja | Colubroidea | 1,5,19 |
| Natator | depressus | 140000 | 6 | NA | 95.15 | 3.5 | NA | NA | 42 | NA | NA | NA | Testudines | 0 | Cheloniidae | Natator | Chelonioidea | 5,26 |
| Natrix | maura | 52.95 | 18.3 | NA | 8.4 | 1.75 | 9.35 | 1095 | 56.5 | 2300 | NA | 40.25 | Squamata | 2 | Natricidae | Natrix | Colubroidea | 11,19 |
| Natrix | natrix | 190.16 | 17.35 | 2.97 | 17.51 | 1.25 | 5.12 | 1369 | 50 | 2300 | 21.42 | 56.2 | Squamata | 2 | Natricidae | Natrix | Colubroidea | 1,4,5,11 |
| Natrix | tessellata | 234.25 | 19.25 | 6.05 | 23.07 | 2 | 8 | NA | 53.67 | 2800 | NA | 40 | Squamata | 2 | Natricidae | Natrix | Colubroidea | 11 |
| Nerodia | sipedon | 118.2 | NA | NA | NA | NA | NA | 730 | NA | NA | 9.6 | NA | Squamata | 2 | Natricidae | Nerodia | Colubroidea | 1,19 |
| Notechis | scutatus | 835.5 | NA | NA | NA | NA | NA | NA | NA | NA | 14.1 | NA | Squamata | 2 | Elapidae | Notechis | Colubroidea | 1,19 |
| Oedura | castelnaui | 14.1 | 5.85 | NA | 1.5 | 4 | 1.78 | 365 | 65.75 | NA | NA | NA | Squamata | 1 | Diplodactylidae | Oedura | Gekkota | 15,17,18 |
| Oedura | lesueurii | 3.7 | 5 | NA | 2 | 2 | NA | NA | NA | NA | NA | NA | Squamata | 1 | Diplodactylidae | Oedura | Gekkota | 15,17,18 |
| Oedura | marmorata | 15.4 | 7 | NA | NA | NA | NA | 365 | 95 | NA | 21.2 | NA | Squamata | 1 | Diplodactylidae | Oedura | Gekkota | 1,15,16,17,18 |
| Oedura | monilis | 6.1 | 6.15 | 0.99 | 16 | 16 | 1.54 | NA | 56 | NA | NA | NA | Squamata | 1 | Diplodactylidae | Oedura | Gekkota | 15,16,18 |
| Oligosoma | grande | 19.4 | NA | NA | NA | NA | NA | NA | NA | NA | NA | NA | Squamata | 1 | Scincidae | Oligosoma | Scincomorpha | 1,18 |
| Oligosoma | otagense | 46 | NA | NA | NA | NA | NA | NA | NA | NA | 44 | NA | Squamata | 1 | Scincidae | Oligosoma | Scincomorpha | 1,18 |
| Opheodrys | aestivus | 17 | NA | NA | NA | NA | NA | 730 | NA | NA | 7.2 | NA | Squamata | 2 | Colubridae | Opheodrys | Colubroidea | 1,19 |
| Ophisaurus | ventralis | 32.2 | NA | NA | NA | NA | NA | NA | NA | NA | 14.8 | NA | Squamata | 1 | Anguidae | Ophisaurus | Diploglossa | 1,18 |
| Ophisops | elegans | 3 | 5 | NA | 4.5 | 2 | NA | 730 | NA | 2200 | NA | NA | Squamata | 1 | Lacertidae | Ophisops | Scincomorpha | 6,18 |
| Osteolaemus | tetraspis | 18800 | 28 | NA | 11.15 | NA | 55 | 1825 | 95 | NA | 69 | NA | Crocodilia | 0 | Crocodylidae | Osteolaemus | NA | 1,2,3 |
| Oxybelis | aeneus | 57.5 | NA | NA | NA | NA | NA | NA | NA | NA | 15.2 | NA | Squamata | 2 | Colubridae | Oxybelis | Colubroidea | 1,19 |
| Oxyuranus | scutellatus | 753 | NA | NA | 35 | NA | NA | NA | NA | NA | 15.6 | NA | Squamata | 2 | Elapidae | Oxyuranus | Colubroidea | 1,5,19 |
| Pachydactylus | capensis | 4 | 41 | 0.32 | 1.5 | 9 | 0.62 | NA | 49 | NA | NA | NA | Squamata | 1 | Gekkonidae | Pachydactylus | Gekkota | 15,18 |
| Paleosuchus | palpebrosus | 5900 | NA | NA | 13.3 | NA | 68.6 | 4197.5 | 135 | NA | 24.1 | NA | Crocodilia | 0 | Alligatoridae | Paleosuchus | NA | 1,3 |
| Paleosuchus | trigonatus | 7500 | NA | NA | 15.1 | NA | 67.2 | 5475 | 115 | 1300 | 30.9 | 135 | Crocodilia | 0 | Alligatoridae | Paleosuchus | NA | 1,3,25 |
| Pantherophis | guttatus | 900 | NA | NA | 12 | NA | NA | 600 | NA | NA | 32.3 | NA | Squamata | 2 | Colubridae | Pantherophis | Colubroidea | 1 |
| Pelodiscus | sinensis | 10000 | 2.7 | NA | 17.5 | 3.5 | NA | 1825 | 60 | NA | NA | NA | Testudines | 0 | Trionychidae | Pelodiscus | Trionychoidea | 5,28 |
| Phelsuma | astriata | 3.4 | 3.5 | NA | NA | NA | NA | NA | 37.5 | NA | 7.4 | NA | Squamata | 1 | Gekkonidae | Phelsuma | Gekkota | 1,16,18 |
| Phelsuma | cepediana | 5 | 4 | NA | NA | NA | NA | NA | 42.5 | NA | 9.3 | NA | Squamata | 1 | Gekkonidae | Phelsuma | Gekkota | 1,18 |
| Phelsuma | laticauda | 2.9 | 4 | NA | 2 | 3.5 | NA | 330 | 46.88 | NA | 8.7 | NA | Squamata | 1 | Gekkonidae | Phelsuma | Gekkota | 1,16,18 |
| Phelsuma | lineata | 3.8 | 4 | NA | 1.75 | 4.5 | NA | 240 | 48.25 | NA | 10 | NA | Squamata | 1 | Gekkonidae | Phelsuma | Gekkota | 1,15,16,18 |
| Phelsuma | madagascariensis | 20.7 | 6.07 | 0.91 | 1.75 | 6 | NA | 367.5 | 56.94 | NA | 13.6 | NA | Squamata | 1 | Gekkonidae | Phelsuma | Gekkota | 1,15,16,18 |
| Phrynocephalus | guttatus | 4.1 | NA | 0.78 | 2 | 1 | NA | 365 | NA | NA | NA | NA | Squamata | 1 | Agamidae | Phrynocephalus | Iguania | 6,18 |
| Phrynocephalus | helioscopus | 6.9 | 4.55 | NA | 4.5 | 1.75 | NA | 345 | 40 | NA | 2.5 | NA | Squamata | 1 | Agamidae | Phrynocephalus | Iguania | 6,18 |
| Phrynocephalus | mystaceus | 34.3 | 4.06 | NA | 2 | 1.5 | NA | 630 | NA | NA | 6 | NA | Squamata | 1 | Agamidae | Phrynocephalus | Iguania | 6,18 |
| Physignathus | cocincinus | 288 | NA | NA | NA | NA | NA | NA | NA | NA | 15.3 | NA | Squamata | 1 | Agamidae | Physignathus | Iguania | 1,18 |
| Physignathus | lesueurii | 559.2 | NA | NA | NA | NA | NA | NA | NA | NA | 18.9 | NA | Squamata | 1 | Agamidae | Physignathus | Iguania | 1,18 |
| Platysaurus | guttatus | 12.2 | NA | NA | NA | NA | NA | NA | NA | NA | 15.6 | NA | Squamata | 1 | Cordylidae | Platysaurus | Scincomorpha | 1,19 |
| Plestiodon | obsoletus | 30 | NA | NA | NA | NA | NA | NA | NA | NA | 7.3 | NA | Squamata | 1 | Scincidae | Plestiodon | Scincomorpha | 1,18 |
| Podarcis | bocagei | 4.6 | NA | NA | 3 | 1 | NA | 730 | NA | 1500 | NA | NA | Squamata | 1 | Lacertidae | Podarcis | Scincomorpha | 8,18 |
| Podarcis | gaigeae | 10.8 | NA | NA | NA | 1 | NA | NA | NA | NA | NA | NA | Squamata | 1 | Lacertidae | Podarcis | Scincomorpha | 8,18 |
| Podarcis | hispanicus | 3.7 | 5.95 | NA | 2.5 | 1 | NA | NA | 63 | 1800 | NA | 3.9 | Squamata | 1 | Lacertidae | Podarcis | Scincomorpha | 8,18 |
| Podarcis | lilfordi | 7.3 | 9.2 | NA | 3 | 1.5 | NA | NA | 44.5 | NA | NA | NA | Squamata | 1 | Lacertidae | Podarcis | Scincomorpha | 8,18 |
| Podarcis | melisellensis | 6 | 6.42 | 0.35 | 4.5 | 3 | NA | 300 | 39 | 1370 | NA | NA | Squamata | 1 | Lacertidae | Podarcis | Scincomorpha | 8,18 |
| Podarcis | muralis | 5.39 | 6.05 | 0.35 | 6 | 2.75 | NA | 365 | 76.25 | 2700 | 8.5 | NA | Squamata | 1 | Lacertidae | Podarcis | Scincomorpha | 1,8,18 |
| Podarcis | peloponnesiacus | 11.6 | 9.83 | NA | 3.5 | 1.83 | NA | NA | 40 | 1550 | NA | NA | Squamata | 1 | Lacertidae | Podarcis | Scincomorpha | 8,18 |
| Podarcis | pityusensis | 6.8 | 9.18 | NA | 1.5 | 1 | NA | NA | NA | NA | NA | NA | Squamata | 1 | Lacertidae | Podarcis | Scincomorpha | 8,18 |
| Podarcis | siculus | 10.75 | 9.13 | 0.65 | 7.17 | 3.1 | NA | 365 | 49.11 | 2000 | 13 | 4.9 | Squamata | 1 | Lacertidae | Podarcis | Scincomorpha | 8,18 |
| Podarcis | tauricus | 7.4 | 7.85 | NA | 5 | 1.5 | NA | 555 | 58 | 800 | NA | 5.4 | Squamata | 1 | Lacertidae | Podarcis | Scincomorpha | 8,18 |
| Podarcis | tiliguerta | 5 | 5.5 | NA | 9 | 1 | NA | NA | 75 | 1800 | NA | NA | Squamata | 1 | Lacertidae | Podarcis | Scincomorpha | 8,18 |
| Podocnemis | expansa | 36250 | 5.08 | NA | 103.8 | NA | NA | NA | 52 | NA | 22.65 | NA | Testudines | 0 | Podocnemididae | Podocnemis | Pelomedusidae | 1,4,5 |
| Podocnemis | unifilis | 6750 | 4 | 15.5 | 25.75 | 2 | NA | NA | 62 | NA | NA | NA | Testudines | 0 | Podocnemididae | Podocnemis | Pelomedusidae | 1,4 |
| Pogona | vitticeps | 500 | NA | NA | NA | NA | NA | NA | NA | NA | 12 | NA | Squamata | 1 | Agamidae | Pogona | Iguania | 1 |
| Psammobates | geometricus | 474 | 3.5 | NA | 5 | 1 | NA | NA | 150 | NA | NA | NA | Testudines | 0 | Testudinidae | Psammobates | Testudinoidea | 23,35 |
| Psammobates | oculifer | 318 | NA | NA | NA | NA | NA | NA | NA | NA | NA | NA | Testudines | 0 | Testudinidae | Psammobates | Testudinoidea | 35 |
| Psammobates | pardalis | 19300 | 5.08 | 30 | 17.5 | 6 | NA | NA | 385 | 2900 | 30 | NA | Testudines | 0 | Testudinidae | Psammobates | Testudinoidea | 1,43,45 |
| Psammobates | tentorius | 508 | 2.75 | 7 | 2 | 1 | NA | NA | 220 | 900 | NA | NA | Testudines | 0 | Testudinidae | Psammobates | Testudinoidea | 28,35 |
| Psammodromus | algirus | 9.1 | NA | NA | 9.5 | 1.5 | NA | NA | 18 | 2400 | NA | NA | Squamata | 1 | Lacertidae | Psammodromus | Scincomorpha | 6,18 |
| Psammodromus | hispanicus | 3 | NA | NA | 4.17 | 1.5 | NA | NA | 48 | NA | NA | NA | Squamata | 1 | Lacertidae | Psammodromus | Scincomorpha | 6,18 |
| Psammophis | subtaeniatus | 68.15 | NA | NA | NA | NA | NA | NA | NA | NA | 5.8 | NA | Squamata | 2 | Lamprophiidae | Psammophis | Colubroidea | 1,19 |
| Pseudaspis | cana | 492.9 | NA | NA | NA | NA | NA | NA | NA | NA | 8.3 | NA | Squamata | 2 | Lamprophiidae | Pseudaspis | Colubroidea | 1,19 |
| Pseudocerastes | persicus | 215.3 | NA | NA | NA | NA | NA | NA | NA | NA | 17.1 | NA | Squamata | 2 | Viperidae | Pseudocerastes | Colubroidea | 1,19 |
| Pseudopus | apodus | 486.63 | NA | NA | 8 | 1 | NA | 730 | 43.5 | 1260 | 54 | NA | Squamata | 1 | Anguidae | Pseudopus | Diploglossa | 1,6,18 |
| Ptychozoon | kuhli | 7.7 | NA | NA | 1.75 | NA | NA | 547.5 | 94.25 | NA | NA | NA | Squamata | 1 | Gekkonidae | Ptychozoon | Gekkota | 4,16,18 |
| Ptyodactylus | hasselquistii | 9.3 | 5.3 | NA | 2 | 5 | NA | 638.75 | 99.25 | 1800 | 10.45 | 10 | Squamata | 1 | Phyllodactylidae | Ptyodactylus | Gekkota | 1,15,16 |
| Python | molurus | 51185 | NA | NA | 40 | NA | NA | 1095 | 100 | NA | 34.2 | NA | Squamata | 2 | Pythonidae | Python | Pythonoidea | 1,5 |
| Python | regius | 1324 | NA | NA | 7 | NA | NA | 1825 | 77 | NA | 47.5 | NA | Squamata | 2 | Pythonidae | Python | Pythonoidea | 1,19 |
| Python | sebae | 13250 | NA | NA | NA | NA | NA | NA | NA | NA | 27.3 | NA | Squamata | 2 | Pythonidae | Python | Pythonoidea | 1,5,19 |
| Pyxis | arachnoides | 398 | NA | NA | 1 | NA | NA | NA | NA | NA | NA | NA | Testudines | 0 | Testudinidae | Pyxis | Testudinoidea | 31 |
| Pyxis | planicauda | 475 | NA | NA | 1 | NA | NA | NA | 100 | NA | NA | NA | Testudines | 0 | Testudinidae | Pyxis | Testudinoidea | 32 |
| Rhinechis | scalaris | 1212 | 27.17 | 14 | 8.93 | 1 | 26 | NA | 58.4 | 2800 | NA | 57.5 | Squamata | 2 | Colubridae | Rhinechis | Colubroidea | 9 |
| Sauromalus | ater | 550 | NA | NA | NA | NA | NA | NA | NA | NA | 9.3 | NA | Squamata | 1 | Iguanidae | Sauromalus | Iguania | 1,18 |
| Sauromalus | hispidus | 737 | NA | NA | NA | NA | NA | NA | NA | NA | 17.2 | NA | Squamata | 1 | Iguanidae | Sauromalus | Iguania | 1,18 |
| Sauromalus | varius | 1800 | NA | NA | NA | NA | NA | NA | NA | NA | 11.3 | NA | Squamata | 1 | Iguanidae | Sauromalus | Iguania | 1,18 |
| Sceloporus | magister | 43.6 | NA | NA | NA | NA | NA | NA | NA | NA | 5.9 | NA | Squamata | 1 | Phrynosomatidae | Sceloporus | Iguania | 1,18 |
| Sceloporus | undulatus | 11.3 | NA | NA | NA | NA | NA | 1095 | NA | NA | NA | NA | Squamata | 1 | Phrynosomatidae | Sceloporus | Iguania | 1,18 |
| Scincus | scincus | 23.3 | NA | NA | 6 | NA | NA | NA | 60 | NA | NA | NA | Squamata | 1 | Scincidae | Scincus | Scincomorpha | 4,18 |
| Sistrurus | catenatus | 167.2 | NA | NA | 8 | NA | NA | 1100 | NA | NA | 20 | NA | Squamata | 2 | Viperidae | Sistrurus | Colubroidea | 1,19 |
| Sistrurus | miliarius | 40.6 | NA | NA | NA | NA | NA | NA | NA | NA | 16.1 | NA | Squamata | 2 | Viperidae | Sistrurus | Colubroidea | 1,19 |
| Spalerosophis | diadema | 211.2 | NA | NA | NA | NA | NA | 547 | NA | NA | 17 | NA | Squamata | 2 | Colubridae | Spalerosophis | Colubroidea | 1,19 |
| Sphaerodactylus | cinereus | 1 | 3.1 | 0.1 | 1 | 10 | 0.16 | NA | 77.5 | NA | NA | NA | Squamata | 1 | Sphaerodactylidae | Sphaerodactylus | Gekkota | 15,16,18 |
| Sphenodon | punctatus | 590 | NA | 4 | 12 | 0.35 | 5 | 5840 | 399 | 87 | 90 | 17.5 | Rhynchocephalia | 0 | Sphenodontidae | Sphenodon | NA | 1,4,5,25,27 |
| Spilotes | pullatus | 552.5 | NA | NA | NA | NA | NA | NA | NA | NA | 17.5 | NA | Squamata | 2 | Colubridae | Spilotes | Colubroidea | 1,19 |
| Storeria | dekayi | 4.7 | NA | NA | NA | NA | NA | 730 | NA | NA | 7 | NA | Squamata | 2 | Natricidae | Storeria | Colubroidea | 1,19 |
| Storeria | occipitomaculata | 7.3 | NA | NA | NA | NA | NA | 730 | NA | NA | 4.6 | NA | Squamata | 2 | Natricidae | Storeria | Colubroidea | 1,19 |
| Tarentola | angustimentalis | 11 | 4.62 | 0.38 | 5.58 | 9.56 | 0.62 | NA | 64.67 | NA | 14.5 | 6 | Squamata | 1 | Phyllodactylidae | Tarentola | Gekkota | 10 |
| Tarentola | annularis | 12.6 | NA | NA | NA | NA | NA | NA | NA | NA | 11.8 | NA | Squamata | 1 | Phyllodactylidae | Tarentola | Gekkota | 1,18 |
| Tarentola | boettgeri | 11.3 | 5.27 | 0.6 | 5 | 3.23 | 0.85 | NA | 90 | 1650 | 18 | 5.15 | Squamata | 1 | Phyllodactylidae | Tarentola | Gekkota | 10 |
| Tarentola | delalandii | 15.72 | 4.17 | 0.51 | 1.75 | 4 | 0.8 | NA | 82.5 | 2300 | 8.5 | NA | Squamata | 1 | Phyllodactylidae | Tarentola | Gekkota | 10,15 |
| Tarentola | gomerensis | 7.3 | NA | NA | 1 | 3.5 | 0.83 | NA | 80 | 1150 | NA | NA | Squamata | 1 | Phyllodactylidae | Tarentola | Gekkota | 10 |
| Tarentola | mauritanica | 7.3 | 5 | NA | 2 | 1 | NA | 1095 | 81.5 | 1283.33 | 10.7 | NA | Squamata | 1 | Phyllodactylidae | Tarentola | Gekkota | 1,6,18 |
| Teira | dugesii | 9 | 8.9 | NA | NA | 1.5 | NA | 450 | 86 | 1826 | 4.8 | NA | Squamata | 1 | Lacertidae | Teira | Scincomorpha | 1,10,18 |
| Telescopus | fallax | 33.2 | 17.83 | 3.5 | 7.92 | 1 | 5 | NA | 55 | 1800 | NA | NA | Squamata | 2 | Colubridae | Telescopus | Colubroidea | 11,19 |
| Telescopus | semiannulatus | 9.5 | NA | NA | NA | NA | NA | NA | NA | NA | 7.6 | NA | Squamata | 2 | Colubridae | Telescopus | Colubroidea | 1,19 |
| Terrapene | carolina | 1000 | 3.15 | NA | 5.5 | 2.5 | NA | 2190 | 85 | NA | 138 | NA | Testudines | 0 | Emydidae | Terrapene | Testudinoidea | 1,4 |
| Testudo | graeca | 1738.67 | 3.25 | 10.8 | 5.05 | 2.17 | 20.7 | 4242.5 | 78 | 1750 | 50.83 | 14.6 | Testudines | 0 | Testudinidae | Testudo | Testudinoidea | 1,13,43,49 |
| Testudo | hermanni | 1045 | 3.2 | 9.97 | 4.49 | 2.13 | 16.05 | 3989.4 | 90 | 1500 | 50 | 13.14 | Testudines | 0 | Testudinidae | Testudo | Testudinoidea | 1,13,30,49 |
| Testudo | horsfieldii | 807 | 3.95 | NA | 8.5 | 2.5 | NA | 3650 | 71.5 | NA | NA | NA | Testudines | 0 | Testudinidae | Testudo | Testudinoidea | 550 |
| Testudo | kleinmanni | 350 | 2.85 | 14 | 2 | NA | NA | NA | 85 | NA | 21 | NA | Testudines | 0 | Testudinidae | Testudo | Testudinoidea | 1,24,43 |
| Testudo | marginata | 1170 | 3.5 | 10.43 | 8.05 | 2.2 | 14.98 | 4380 | 76.25 | 1300 | 58.2 | 21.6 | Testudines | 0 | Testudinidae | Testudo | Testudinoidea | 1,13,30,43 |
| Thamnophis | butleri | 24.8 | NA | NA | 12 | NA | NA | 730 | NA | NA | 14 | NA | Squamata | 2 | Natricidae | Thamnophis | Colubroidea | 1,19 |
| Thamnophis | couchii | 61 | NA | NA | NA | NA | NA | NA | NA | NA | 7.7 | NA | Squamata | 2 | Natricidae | Thamnophis | Colubroidea | 1,19 |
| Thamnophis | marcianus | 90 | NA | NA | NA | NA | NA | 547 | NA | NA | 7 | NA | Squamata | 2 | Natricidae | Thamnophis | Colubroidea | 1,19 |
| Thamnophis | radix | 50.9 | NA | NA | NA | NA | NA | NA | NA | NA | 8.4 | NA | Squamata | 2 | Natricidae | Thamnophis | Colubroidea | 1,19 |
| Thamnophis | sirtalis | 127.1 | NA | NA | 20 | 0.75 | NA | 730 | 88 | NA | 14.1 | NA | Squamata | 2 | Natricidae | Thamnophis | Colubroidea | 1,4,19 |
| Tiliqua | nigrolutea | 800 | NA | NA | NA | NA | NA | NA | NA | NA | 11.5 | NA | Squamata | 1 | Scincidae | Tiliqua | Scincomorpha | 1,18 |
| Tiliqua | rugosa | 617 | NA | NA | NA | NA | NA | NA | NA | NA | 20.9 | NA | Squamata | 1 | Scincidae | Tiliqua | Scincomorpha | 1,5,18 |
| Tiliqua | scincoides | 499.8 | NA | 14 | 17.5 | NA | NA | NA | 110 | NA | 23.3 | NA | Squamata | 1 | Scincidae | Tiliqua | Scincomorpha | 1,4,5,18 |
| Timon | lepidus | 213.28 | 11.76 | 2.19 | 13.38 | 1.5 | NA | 1026 | 91.75 | 2100 | 18.5 | NA | Squamata | 1 | Lacertidae | Timon | Scincomorpha | 1,7,18 |
| Tomistoma | schlegelii | 119000 | NA | NA | 31.8 | NA | 139.9 | 7300 | 102.5 | 20 | 24.7 | 275 | Crocodilia | 0 | Crocodylidae | Tomistoma | NA | 1,2,3,25 |
| Trachemys | scripta | 2513.33 | 3.48 | 8.1 | 16 | 2.06 | 10.75 | 2920 | 82.25 | NA | 39.33 | 17.15 | Testudines | 0 | Emydidae | Trachemys | Testudinoidea | 1,13 |
| Trimorphodon | biscutatus | 84.3 | NA | NA | NA | NA | NA | NA | NA | NA | 11.8 | NA | Squamata | 2 | Colubridae | Trimorphodon | Colubroidea | 1,19 |
| Tupinambis | rufescens | 4700 | NA | NA | NA | NA | NA | NA | NA | NA | 10.8 | NA | Squamata | 1 | Teiidae | Tupinambis | Scincomorpha | 1,18 |
| Tupinambis | teguixin | 2212 | NA | NA | 13.75 | NA | 32.5 | NA | 77 | NA | 16.1 | NA | Squamata | 1 | Teiidae | Tupinambis | Scincomorpha | 1,4,5,18 |
| Typhlops | vermicularis | 2.2 | NA | NA | 5 | 1 | NA | NA | NA | 1900 | NA | NA | Squamata | 2 | Typhlopidae | Typhlops | Typhlopoidea | 5,9,19 |
| Uma | notata | 26.9 | NA | NA | NA | NA | NA | NA | NA | NA | 8.1 | NA | Squamata | 1 | Phrynosomatidae | Uma | Iguania | 1,18 |
| Underwoodisaurus | milii | 9.6 | NA | NA | NA | NA | NA | NA | NA | NA | 12 | NA | Squamata | 1 | Carphodactylidae | Underwoodisaurus | Gekkota | 1,18 |
| Uromastyx | acanthinura | 600 | NA | NA | NA | NA | NA | NA | NA | NA | 11.4 | NA | Squamata | 1 | Agamidae | Uromastyx | Iguania | 1,18 |
| Uromastyx | aegyptia | 851.5 | NA | NA | NA | NA | NA | NA | NA | NA | 15.3 | NA | Squamata | 1 | Agamidae | Uromastyx | Iguania | 1,18 |
| Varanus | bengalensis | 4940 | NA | NA | NA | NA | NA | NA | NA | NA | 11.2 | NA | Squamata | 1 | Varanidae | Varanus | Platynota | 1,18 |
| Varanus | brevicauda | 14.9 | NA | NA | NA | NA | NA | NA | NA | NA | NA | NA | Squamata | 1 | Varanidae | Varanus | Platynota | 5,18 |
| Varanus | caudolineatus | 14.3 | NA | NA | NA | NA | NA | NA | NA | NA | NA | NA | Squamata | 1 | Varanidae | Varanus | Platynota | 18 |
| Varanus | dumerilii | 988 | NA | NA | NA | NA | NA | NA | NA | NA | 10.7 | NA | Squamata | 1 | Varanidae | Varanus | Platynota | 1,18 |
| Varanus | exanthematicus | 18104.5 | NA | NA | NA | NA | NA | NA | NA | NA | 12.7 | NA | Squamata | 1 | Varanidae | Varanus | Platynota | 1,5,18 |
| Varanus | giganteus | 5333.4 | NA | NA | NA | NA | NA | NA | NA | NA | 19.7 | NA | Squamata | 1 | Varanidae | Varanus | Platynota | 1,5,18 |
| Varanus | gouldii | 821.1 | NA | NA | NA | NA | NA | NA | NA | NA | 18.3 | NA | Squamata | 1 | Varanidae | Varanus | Platynota | 1,5,18 |
| Varanus | griseus | 1221.5 | NA | NA | NA | NA | NA | NA | NA | NA | 17 | NA | Squamata | 1 | Varanidae | Varanus | Platynota | 1,5,18 |
| Varanus | indicus | 1287 | NA | NA | NA | NA | NA | NA | NA | NA | 17.4 | NA | Squamata | 1 | Varanidae | Varanus | Platynota | 1,5,18 |
| Varanus | komodoensis | 77820 | 30 | 100 | 26 | NA | NA | 1825 | 144.5 | NA | 25.5 | NA | Squamata | 1 | Varanidae | Varanus | Platynota | 1,4,5,18 |
| Varanus | mertensi | 1121.2 | NA | NA | NA | NA | NA | NA | NA | NA | 20.3 | NA | Squamata | 1 | Varanidae | Varanus | Platynota | 1,18 |
| Varanus | niloticus | 2890 | NA | NA | NA | NA | NA | NA | NA | NA | 14.6 | NA | Squamata | 1 | Varanidae | Varanus | Platynota | 1,5,18 |
| Varanus | olivaceus | 5420 | NA | NA | NA | NA | NA | NA | NA | NA | 15.2 | NA | Squamata | 1 | Varanidae | Varanus | Platynota | 1,18 |
| Varanus | prasinus | 237.5 | NA | NA | NA | NA | NA | NA | NA | NA | 14.2 | NA | Squamata | 1 | Varanidae | Varanus | Platynota | 1,18 |
| Varanus | salvator | 4345 | NA | NA | NA | NA | NA | NA | NA | NA | 15.7 | NA | Squamata | 1 | Varanidae | Varanus | Platynota | 1,5,18 |
| Varanus | timorensis | 290 | NA | NA | NA | NA | NA | NA | NA | NA | 14.9 | NA | Squamata | 1 | Varanidae | Varanus | Platynota | 1,18 |
| Varanus | varius | 6343 | NA | NA | NA | NA | NA | NA | NA | NA | 14.7 | NA | Squamata | 1 | Varanidae | Varanus | Platynota | 1,5,18 |
| Vipera | ammodytes | 210.9 | 18.65 | 5.67 | 9.56 | 0.75 | NA | NA | 102.5 | 2450 | 17.67 | 43.3 | Squamata | 2 | Viperidae | Vipera | Colubroidea | 1,12,19 |
| Vipera | aspis | 74.1 | 19.7 | 6.63 | 14.16 | 0.69 | NA | 1369 | 78 | 3000 | 19.5 | 45 | Squamata | 2 | Viperidae | Vipera | Colubroidea | 1,12,19 |
| Vipera | berus | 153.15 | 18.3 | NA | 10.33 | 2 | NA | 1551.5 | 94.33 | NA | 19 | 45.5 | Squamata | 2 | Viperidae | Vipera | Colubroidea | 1,4,5,12 |
| Vipera | kaznakovi | 52.1 | 15.6 | 4.12 | NA | 1 | NA | 913 | NA | 1000 | NA | 37.5 | Squamata | 2 | Viperidae | Vipera | Colubroidea | 12,19 |
| Vipera | latastei | 87 | 17.67 | 5.34 | 7.91 | 0.88 | NA | 1156 | 90 | 3030 | 9.7 | 42 | Squamata | 2 | Viperidae | Vipera | Colubroidea | 1,12,19 |
| Vipera | seoanei | 90 | 19.03 | 5.22 | 5.92 | 0.88 | NA | NA | NA | 1900 | NA | NA | Squamata | 2 | Viperidae | Vipera | Colubroidea | 12 |
| Vipera | ursinii | 65 | 13.75 | 3.03 | 8.78 | 1 | NA | NA | NA | 2000 | NA | NA | Squamata | 2 | Viperidae | Vipera | Colubroidea | 12,19 |
| Virginia | striatula | 3.3 | NA | NA | NA | NA | NA | 730 | NA | NA | 7.2 | NA | Squamata | 2 | Natricidae | Virginia | Colubroidea | 1,19 |
| Virginia | valeriae | 7.2 | NA | NA | NA | NA | NA | NA | NA | NA | 9.5 | NA | Squamata | 2 | Natricidae | Virginia | Colubroidea | 1,19 |
| Xantusia | henshawi | 3.2 | NA | NA | NA | NA | NA | NA | NA | NA | 14.3 | NA | Squamata | 1 | Xantusiidae | Xantusia | Scincomorpha | 1,18 |
| Xantusia | riversiana | 17.3 | NA | NA | NA | NA | NA | NA | NA | NA | 13.9 | NA | Squamata | 1 | Xantusiidae | Xantusia | Scincomorpha | 1,18 |
| Xantusia | vigilis | 1.5 | NA | NA | 5 | NA | NA | NA | NA | NA | 10.9 | NA | Squamata | 1 | Xantusiidae | Xantusia | Scincomorpha | 1,18 |
| Zamenis | longissimus | 783.33 | 23.85 | NA | 10.6 | 1 | NA | 1460 | 60 | 1700 | 20.33 | 96.58 | Squamata | 2 | Colubridae | Zamenis | Colubroidea | 9 |

**Table S1b** References of our reptile database (Table S1a).

(1) Tacutu R, Craig T, Budovsky A, Wuttke D, Lehmann G, Taranukha D, et al. 2013. Human Ageing Genomic Resources: integrated databases and tools for the biology and genetics of ageing. Nucleic Acids Res. Oxford Univ Press; 41: D1027–D1033.

(2) Britton A. 2012. Crocodilians: Natural history & conservation - crocodiles, caimans, alligators, gharials. Retrieved May 19, 2015, from http://crocodilian.com.

(3) Thorbjarnarson J. 1996. Reproductive characteristics of the order Crocodylia. Herpetologica 52(1):8-24.

(4) Green J., Spilsbury R., Taylor B. 2009. Exploring the world of reptiles and amphibians. 1 ed. Chelsea House, New York.

(5) Deckert K. 1991. Die große farbige Enzyklopädie Urania-Tierreich - Fische, Lurche, Kriechtiere. 1 ed. Urania-Verlagsgesellschaft mbH, Leipzig.

(6) Böhme W. 1981. Handbuch der Reptilien und Amphibien Europas, Band 1: Echsen (Sauria) I (Gekkonidae, Agamidae, Chamaeleonidae, Anguidae, Amphisbaenidae, Scincidae, Lacertidae I). 1. ed. Akademische Verlagsgesellschaft, Wiesbaden.

(7) Böhme W. 1984. Handbuch der Reptilien und Amphibien Europas, Band 2/I: Echsen (Sauria) II (Lacertidae II: Lacerta). 1. ed. AULA-Verlag, Wiesbaden.

(8) Böhme W. 1986. Handbuch der Reptilien und Amphibien Europas, Band 2/II: Echsen (Sauria) III (Lacertidae III: Podarcis). 1. ed. AULA-Verlag, Wiesbaden.

(9) Böhme W. 1993. Handbuch der Reptilien und Amphibien Europas, Band 3/I: Schlangen (Serpentes) I (Typhlopidae, Boidae, Colubridae 1: Colubrinae). 1. ed. AULA-Verlag, Wiesbaden.

(10) Bischoff W. 1998. Handbuch der Reptilien und Amphibien Europas, Band 6: Die Reptilien der Kanarischen Inseln, der Selvagens-Inseln und des Madeira Archipels. 1. ed. AULA-Verlag, Wiesbaden.

(11) Böhme W. 1999. Handbuch der Reptilien und Amphibien Europas, Band 3/IIA: Schlangen (Serpentes) II (Colubridae 2: Boiginae, Natricinae). 1. ed. AULA-Verlag, Wiebelsheim.

(12) Joger U, Stümpel N. 2005. Handbuch der Reptilien und Amphibien Europas, Band 3/IIB: Schlangen (Serpentes) III (Viperidae). 1. ed. AULA-Verlag, Wiebelsheim.

(13) Fritz U. 2001. Handbuch der Reptilien und Amphibien Europas, Band 3/III A: Bataguridae, Testudinidae, Emydidae (Land- und Sumpfschildkröten). 1 ed. AULA-Verlag, Wiebelsheim.

(14) Fritz U. 2005. Handbuch der Reptilien und Amphibien Europas, Band 3/III B: Cheloniidae, Dermochelyidae, Fossile Schildkröten Europas (See- und Lederschildkröten). 1 ed. AULA-Verlag, Wiebelsheim.

(15) Rösler H. 1995. Geckos der Welt: alle Gattungen. 1. ed. Leipzig, Jena, Berlin: Urania-Verlagsgesellschaft mbH.

(16) Rogner M. 1992. Echsen 1: Haltung, Pflege und Zucht im Terrarium - Geckos, Flossenfüsse, Agamen, Chamäleons und Leguane. 1. ed. Stuttgart: Eugen Ulmer GmbH & Co.

(17) Henkel F-W, Schmidt W. 1991. Geckos - Biologie, Haltung und Zucht. 1. ed. Stuttgart: Eugen Ulmer GmbH & Co.

(18) Meiri S. 2010. Length-weight allometries in lizards. Journal of Zoology 281:218-226.

(19) Feldman A., Meiri S. 2013. Length-mass allomtery in snakes. Biological Journal of the Linnean Society 108(1):161-172.

(20) Barros MS., Resende LC., Silva AG., Ferreira Junior PD. 2012. Morphological varations and sexual dimorphism in *Chelonoides carbonaria* (Spix, 1824) and *Chelonoides denticulata* (Linnaeus, 1766) (Testudinidae). Braz. J. Biol. 72(1):153-161.

(21) Oyewale J.O., Ebute C.P., Ogunsanmi O., Olayemi F.O., Durotoye L.A. 1998. Weights and blood profiles of the West African hinge-backed tortoise, *Kinixys erosa* and the desert tortoise, *Gopherus agassizii* . J. Vet. Med. A 45:599-605.

(22) Luisell L., Diagne T. 2013. *Kinixys homeana* Bell 1827 – Home’s Hinge-Back Tortoise. In: Rhodin, A.G.J., Pritchard, P.C.H., van Dijk, P.P., Saumure, R.A., Buhlmann, K.A., Iverson, J.B., and Mittermeier, R.A. (Eds.). Conservation biology of freshwater turtles and tortoises:

A compilation project of the IUCN/SSC tortoise and freshwater turtle specialist group. Chelonian Research Monographs No. 5, pp. 070.1–070.10, doi:10.3854/crm.5.070.homeana.v1.2013, http://www.iucn-tftsg.org/cbftt/.

(23) Groombridge B., Wright L. 1982. The IUCN Amphibia - Reptilia Red Data Book. Part 1: Testudines, Crocodylia, Rhynchocephalia. 1 ed. IUCN Conservation Monitoring Center, Cambridge.

(24) Geffen E., Mendelssohn H. 1989. Activity patterns and thermoregulatory behavior of the Egyptian tortoise *Testudo kleinmanni* in Israel. Journal of Herpetology 23(4):404-409.

(25) Myers, P., R. Espinosa, C. S. Parr, T. Jones, G. S. Hammond, and T. A. Dewey. 2015. The Animal Diversity Web (online). Accessed at http://animaldiversity.org.

(26)Burbidge, A. A. (2004). Threatened animals of Western Australia. Department of Conservation and Land Management. pp. 110, 114.

(27) Rheubert J. L., Siegel D. S., Trauth S. E. (eds.) 2014. Reproductive biology and phylogeny of lizards and tuatara. 1 ed. CRC Press, London.

(28) Ernst, C. H., and R. W. Barbour. 1989. Turtles of the world. Smithsonian Inst. Press, Washington, D. C.

(29) Coulson, I. M., and A. Hailey. 2001. Low survival rate and high predation in the African hingeback tortoise *Kinixys spekii*. Pp. 383-392.

(30) Rogner, M. 1996. Schildkröten 2. Heidi Rogner-Verlag, Hürtgenwald.

(31) Walker, R. C. J., A. J. Woods-Ballard, and C. E. Rix. 2007. Population density and seasonal activity of the threatened Madagascar spider tortoise (*Pyxis arachnoides arachnoides*) of the southern dry forests; South West Madagascar. Afr. J. Ecol. 46:67–73.

(32) http://www.arkive.org/flat-shelled-spider-tortoise/pyxis-planicauda/,Authenticated (27/10/08) by Dr Richard Young, Conservation Biologist, Durrell Wildlife Conservation Trust. http://www.durrell.org

(33) Hofmeyr, M. D. 2004. Egg production in *Chersina angulata*: an unusual pattern in a Mediterranean climate. J. Herpetol. 38:172-179.

(34) Loehr, V. J. T., B. T. Henen, and M. D. Hofmeyr. 2004. Reproduction of the smallest tortoise, the namaqualand speckled padloper, *Homopus signatus* *signatus*. Herpetologica 60:444-454.

(35) Hofmeyr, M. D., B. T. Henen, and V. J. T. Loehr. 2005. Overcoming environmental and morphological constraints: egg size and pelvic kinesis in the smallest tortoise, *Homopus signatus*. Can. J. Zool./Rev. Can. Zool. 83:1343-1352.

(36) Abou-Madi, N., and E. R. Jacobson. 2003. Effects of blood processing techniques on Sodium and Potassium values: a comparison between Aldabra tortoises (*Geochelone gigantea*) and Burmese mountain tortoises (*Manouria emys*). Vet. Clin. Path. 32:61-66.

(37) McKeown, S., D. Meier, J. Juvik. 1991. The Management and breeding of the Asian forest tortoise (*Manouria emys*) in Captivity. Proceedings of the First International Symposium on Turtles & Tortoises: Conservation and Captive Husbandry: 138-159. Accessed May 22, 2015 at http://www.tortoise.org/archives/manouria.html.

(38) Ives, I., P. Spinks, and H. Shaffer. 2007. Morphological and genetic variation in the endangered Sulawesi tortoise *Indotestudo forstenii*: evidence of distinct lineages? Conserv. Genet. 9(8):709-713.

(39) Stevenson, P., C. Borda, A. Rojas, and M. Álvarez. 2007. Population size, habitat choice and sexual dimorphism of the Amazonian tortoise (*Geochelone denticulata*) in Tinigua National Park, Colombia. Amphibia-Reptilia 28:217-226

(40) Zani, P. A., J. S. Gottschall, and R. Kram. 2005. Giant Galapagos tortoises walk without inverted pendulum mechanical-energy exchange. J. Exp. Biol. 208:1489-1494.

(41) Stearns, B. C. 1988. The captive status of the African spurred tortoise *Geochelone sulcata*: recent developments. International Zoo Yearbook 28:87-98.

(42) Burchfield, P. M., C. S. Doucette, and T. F. Beimler. 1980. Captive management of the radiated tortoise *Geochelone radiata* at Gladys Porter Zoo, Brownsville. International Zoo Yearbook 20:1-6.

(43) Bonin, F., B. Devaux, and A. Dupré. 2006. Turtles of the world. Johns Hopkins Univ. Press, Baltimore.

(44) Ewert, M. A., R. E. Hatcher, and J. M. Goode. 2004. Sex determination and ontogeny in *Malacochersus tornieri*, the pancake tortoise. J. Herpetol. 38:291-295.

(45) Hailey, A., and I. M. Coulson. 1999. The growth pattern of the African tortoise *Geochelone pardalis* and other chelonians. Canadian Journal of Zoology-Revue Canadienne De Zoologie 77:181-193.

(46) Henen, B. T. 1997. Seasonal and annual energy budgets of female desert tortoises (*Gopherus agassizii*). Ecology 78:283-296.

(47) Hellgren, E. C., R. T. Kazmaier, D. C. R. Iii, and D. R. Synatzske. 2000. Variation in tortoise life history: Demography of *Gopherus berlandieri*. Ecology 81:1297-1310.

(48) Bjorndal, K. A. 1987. Digestive Efficiency in a temperate herbivorous reptile, *Gopherus polyphemus*. Copeia:714-720.

(49) Mason, M. C., Kerley, G. I. H., Weathreby, C. A. and Branch, W. R. (2000), Angulate and leopard tortoises in the Thicket Biome, Eastern Cape, South Africa: populations and biomass estimates. African Journal of Ecology, 38: 147–153.

(49) Willemsen, R. E., and A. Hailey. 2002. Body mass condition in Greek tortoises: regional and interspecific variation. Herpetol. J. 12:105-114.

(50) Lagarde, F., X. Bonnet, B. Henen, A. Legrand, J. Corbin, K. Nagy, and G. Naulleau. 2003. Sex divergence in space utilisation in the steppe tortoise (*Testudo horsfieldi*). Can. J. Zool. 81:380-387.

Table S2 Allometric relationships on life-history traits of reptiles. Relationships are calculated by generalized least squares (GLS) regression and phylogenetic-informed GLS regression (PGLS) analysis of log_10_-log_10_-transformed data with adult weight as the independent variable. The phylogenetic tree used by PGLS is a composite tree constructed from different recently published phylogenies on the studied reptilian taxa (see main text, Figure S1). df = degree of freedom, CI_i_ = confidence interval of intercept, CI_s_ = confidence interval of slope, λ = Pagels’ lambda as measure of phylogenetic impact (Pagel 1991). The significance of λ is assessed from likelihood ratio tests between model types GLS and PGLS: Χ^2^ = Chi-squared statistic; P(Χ^2^) = corresponding p-value.

| Trait | Statistics | Intercept | P-value | 95%CI_i_ | Slope | P-value | 95%CI_s_ | df | λ | Χ^2^ | P(Χ^2^) |
| --- | --- | --- | --- | --- | --- | --- | --- | --- | --- | --- | --- |
|  |  |  |  |  |  |  |  |  |  |  |  |
| birth size TL | PGLS | 0.575 | <0.001 | 0.333, 0.817 | 0.129 | <0.001 | 0.080, 0.177 | 144 | 0.84 | 141.20 | <0.001 |
|  | GLS | 0.801 | <0.001 | 0.694, 0.908 | 0.030 | 0.127 | -0.009, 0.069 | 145 | - |  |  |
| birth weight | PGLS | -0.358 | 0.027 | -0.675, -0.041 | 0.411 | <0.001 | 0.318, 0.505 | 77 | 0.26 | 3.08 | 0.08 |
|  | GLS | -0.400 | <0.001 | -0.605, -0.195 | 0.442 | <0.001 | 0.374, 0.511 | 77 | - |  |  |
| clutch size | PGLS | 0.370 | 0.006 | 0.109, 0.631 | 0.234 | <0.001 | 0.185, 0.284 | 208 | 0.77 | 113.90 | <0.001 |
|  | GLS | 0.311 | <0.001 | 0.214, 0.408 | 0.235 | <0.001 | 0.201, 0.269 | 211 | - |  |  |
| clutches p.a. | PGLS | 0.287 | 0.012 | 0.065, 0.510 | -0.001 | 0.968 | -0.056, 0.054 | 134 | 0.67 | 58.09 | <0.001 |
|  | GLS | 0.228 | <0.001 | 0.140, 0.315 | 0.003 | 0.862 | -0.034, 0.040 | 136 | - |  |  |
| egg weight | PGLS | -0.343 | 0.029 | -0.650, -0.037 | 0.450 | <0.001 | 0.371, 0.529 | 61 | 0.85 | 33.44 | <0.001 |
|  | GLS | -0.524 | <0.001 | -0.683, -0.366 | 0.520 | <0.001 | 0.475, 0.566 | 61 | - |  |  |
| female maturity | PGLS | 2.820 | <0.001 | 2.661, 2.980 | 0.118 | <0.001 | 0.079, 0.157 | 119 | 0.55 | 32.90 | <0.001 |
|  | GLS | 2.602 | <0.001 | 2.524, 2.680 | 0.197 | <0.001 | 0.170, 0.223 | 120 | - |  |  |
| incubation time | PGLS | 1.817 | <0.001 | 1.670, 1.964 | 0.030 | 0.057 | -0.001, 0.061 | 174 | 0.72 | 65.70 | <0.001 |
|  | GLS | 1.794 | <0.001 | 1.737, 1.850 | 0.041 | <0.001 | 0.022, 0.060 | 175 | - |  |  |
| max. longevity | PGLS | 0.918 | <0.001 | 0.746, 1.090 | 0.127 | <0.001 | 0.095, 0.159 | 274 | 0.73 | 93.18 | <0.001 |
|  | GLS | 0.853 | <0.001 | 0.792, 0.914 | 0.147 | <0.001 | 0.125, 0.169 | 279 | - |  |  |
| size at maturity | PGLS | 0.751 | <0.001 | 0.459, 1.043 | 0.272 | <0.001 | 0.202, 0.343 | 53 | 0.93 | 74.74 | <0.001 |
|  | GLS | 0.704 | <0.001 | 0.472, 0.935 | 0.295 | <0.001 | 0.227, 0.363 | 53 | - |  |  |

Table S3 Allometric relationships of life-history traits in lizards. Relationships are calculated by generalized least squares (GLS) regression and phylogenetically-informed GLS regression (PGLS) analysis of log_10_-log_10_-transformed data with adult weight as the independent variable. Phylogeny taken from Pyron & Burbrink (2014). df = degree of freedom, CI_i_ = confidence interval of intercept, CI_s_ = confidence interval of slope, λ = Pagels’ lambda as measure of phylogenetic impact (Pagel 1991). The significance of λ is assessed from likelihood ratio tests between model types GLS and PGLS: Χ^2^ = Chi-squared statistic; P(Χ^2^) = corresponding p-value.

| Trait | Statistics | Intercept | P-value | 95%CI_i_ | Slope | P-value | 95%CI_s_ | df | λ | Χ^2^ | P(Χ^2^) |
| --- | --- | --- | --- | --- | --- | --- | --- | --- | --- | --- | --- |
|  |  |  |  |  |  |  |  |  |  |  |  |
| birth size TL | PGLS | 0.722 | <0.001 | 0.366, 1.077 | 0.106 | 0.020 | 0.018, 0.195 | 63 | 1.04 | 0.52 | 0.47 |
|  | GLS | 0.638 | <0.001 | 0.538, 0.739 | 0.169 | <0.001 | 0.094, 0.244 | 63 | - |  |  |
| birth weight | PGLS | -0.725 | <0.001 | -1.039, -0.412 | 0.611 | <0.001 | 0.383, 0.839 | 27 | -0.09 | 0.72 | 0.40 |
|  | GLS | -0.684 | 0.001 | -1.072, -0.296 | 0.578 | <0.001 | 0.333, 0.823 | 27 | - |  |  |
| clutch size | PGLS | 0.465 | <0.001 | 0.230, 0.700 | 0.139 | 0.002 | 0.053, 0.224 | 88 | 0.58 | 27.33 | <0.001 |
|  | GLS | 0.361 | <0.001 | 0.232, 0.490 | 0.201 | <0.001 | 0.122, 0.281 | 90 | - |  |  |
| clutches p.a. | PGLS | 0.249 | 0.030 | 0.025, 0.473 | -0.022 | 0.679 | -0.127, 0.084 | 70 | 0.57 | 21.97 | <0.001 |
|  | GLS | 0.407 | <0.001 | 0.267, 0.547 | -0.129 | 0.029 | -0.244, -0.013 | 71 | - |  |  |
| egg weight | PGLS | -0.812 | <0.001 | -1.150, -0.473 | 0.662 | <0.001 | 0.509, 0.816 | 18 | 0.87 | 8.23 | 0.004 |
|  | GLS | -0.740 | <0.001 | -0.944, -0.535 | 0.592 | <0.001 | 0.451, 0.734 | 18 | - |  |  |
| female maturity | PGLS | 2.737 | <0.001 | 2.577, 2.898 | 0.085 | 0.010 | 0.021, 0.149 | 41 | 0.58 | 4.41 | 0.036 |
|  | GLS | 2.637 | <0.001 | 2.539, 2.735 | 0.124 | <0.001 | 0.063, 0.184 | 41 | - |  |  |
| incubation time | PGLS | 1.724 | <0.001 | 1.580, 1.869 | 0.068 | 0.012 | 0.016, 0.120 | 74 | 0.70 | 6.78 | 0.009 |
|  | GLS | 1.765 | <0.001 | 1.697, 1.832 | 0.043 | 0.049 | 0.000, 0.085 | 74 | - |  |  |
| max. longevity | PGLS | 0.827 | <0.001 | 0.672, 0.982 | 0.136 | <0.001 | 0.087, 0.186 | 124 | 0.70 | 31.99 | <0.001 |
|  | GLS | 0.892 | <0.001 | 0.807, 0.976 | 0.098 | <0.001 | 0.062, 0.135 | 125 | - |  |  |
| size at maturity | PGLS | 0.547 | 0.006 | 0.213, 0.881 | 0.253 | <0.001 | 0.110, 0.396 | 9 | 1.10 | 3.43 | 0.06 |
|  | GLS | 0.471 | 0.006 | 0.188, 0.755 | 0.294 | 0.003 | 0.048, 0.540 | 9 | - |  |  |

Table S4 Allometric relationships of life-history traits in snakes. Relationships are calculated by generalized least squares (GLS) regression and phylogenetically-informed GLS regression (PGLS) analysis of log_10_-log_10_-transformed data with adult weight as the independent variable. Phylogeny taken from Pyron & Burbrink (2014). df = degree of freedom, CI_i_ = confidence interval of intercept, CI_s_ = confidence interval of slope, λ = Pagels’ lambda as measure of phylogenetic impact (Pagel 1991). The significance of λ is assessed from likelihood ratio tests between model types GLS and PGLS: Χ^2^ = Chi-squared statistic; P(Χ^2^) = corresponding p-value.

| Trait | Statistics | Intercept | P-value | 95%CI_i_ | Slope | P-value | 95%CI_s_ | df | λ | Χ^2^ | P(Χ^2^) |
| --- | --- | --- | --- | --- | --- | --- | --- | --- | --- | --- | --- |
|  |  |  |  |  |  |  |  |  |  |  |  |
| birth size TL | PGLS | 0.844 | 0.007 | 0.251, 1.436 | 0.202 | 0.009 | 0.056, 0.349 | 28 | 1.05 | 31.99 | <0.001 |
|  | GLS | 0.816 | <0.001 | 0.579, 1.053 | 0.199 | <0.001 | 0.106, 0.292 | 29 | - |  |  |
| birth weight | PGLS | -0.117 | 0.539 | -0.519, 0.284 | 0.398 | <0.001 | 0.240, 0.558 | 15 | 0.40 | 0.33 | 0.57 |
|  | GLS | -0.077 | 0.650 | -0.433, 0.279 | 0.377 | <0.001 | 0.226, 0.528 | 16 | - |  |  |
| clutch size | PGLS | 0.522 | 0.023 | 0.075, 0.969 | 0.243 | <0.001 | 0.154, 0.332 | 48 | 0.93 | 3.92 | <0.05 |
|  | GLS | 0.553 | <0.001 | 0.357, 0.749 | 0.211 | <0.001 | 0.137, 0.285 | 49 | - |  |  |
| clutches p.a. | PGLS | -0.011 | 0.892 | -0.174, 0.153 | 0.013 | 0.719 | -0.061, 0.087 | 28 | 0.09 | 0.34 | 0.56 |
|  | GLS | -0.010 | 0.899 | -0.165, 0.146 | 0.012 | 0.737 | -0.059, 0.082 | 29 | - |  |  |
| egg weigth | PGLS | -0.081 | 0.751 | -0.638, 0.476 | 0.433 | 0.002 | 0.201, 0.666 | 11 | -0.44 | 0.86 | 0.35 |
|  | GLS | 0.004 | 0.988 | -0.598, 0.606 | 0.401 | 0.005 | 0.155, 0.646 | 11 | - |  |  |
| female maturity | PGLS | 2.814 | <0.001 | 2.662, 2.966 | 0.089 | 0.005 | 0.028, 0.151 | 39 | -0.01 | 0.21 | 0.65 |
|  | GLS | 2.814 | <0.001 | 2.660, 2.968 | 0.088 | 0.006 | 0.026, 0.149 | 40 | - |  |  |
| incubation time | PGLS | 1.775 | <0.001 | 1.485, 2.065 | 0.059 | 0.142 | -0.021, 0.138 | 32 | 0.83 | 5.91 | 0.015 |
|  | GLS | 1.664 | <0.001 | 1.457, 1.871 | 0.086 | 0.023 | 0.013, 0.159 | 33 | - |  |  |
| max. longevity | PGLS | 1.002 | <0.001 | 0.805, 1.198 | 0.106 | <0.001 | 0.055, 0.158 | 99 | 0.70 | 7.21 | 0.007 |
|  | GLS | 0.851 | <0.001 | 0.743, 0.960 | 0.155 | <0.001 | 0.111, 0.199 | 103 | - |  |  |
| size at maturity | PGLS | 1.107 | <0.001 | 0.771, 1.444 | 0.272 | 0.001 | 0.130, 0.414 | 16 | -0.08 | 16.60 | <0.001 |
|  | GLS | 1.139 | <0.001 | 0.791, 1.487 | 0.260 | 0.002 | 0.114, 0.406 | 16 | - |  |  |
|  |  |  |  |  |  |  |  |  |  |  |  |

Table S5 Allometric relationships of life history traits of the crocodiles. Relationships are calculated by generalized least squares (GLS) regression and phylogenetically-informed GLS regression (PGLS) analysis of log_10_-log_10_-transformed data with adult weight as the independent variable. Phylogeny taken from Oaks (2011). df = degree of freedom. CI_i_ = confidence interval of intercept, CI_s_ = confidence interval of slope, λ = Pagels’ lambda as measure of phylogenetic impact (Pagel 1991). The significance of λ is assessed from likelihood ratio tests between model types GLS and PGLS: Χ^2^ = Chi-squared statistic; P(Χ^2^) = corresponding p-value.

| Trait | Statistics | Intercept | P-value | CI_i_ | Slope | P-value | CI_s_ | df | λ | Χ^2^ | P(Χ^2^) |
| --- | --- | --- | --- | --- | --- | --- | --- | --- | --- | --- | --- |
|  |  |  |  |  |  |  |  |  |  |  |  |
| birth size TL | PGLS | - | - | - | - | - | - | 6 | - |  |  |
|  | GLS | - | - | - | - | - | - | 6 | - |  |  |
| birth weight | PGLS | - | - | - | - | - | - | 4 | - |  |  |
|  | GLS | - | - | - | - | - | - | 4 | - |  |  |
| clutch size | PGLS | -0.655 | 0.142 | -1.547, 0.238 | 0.461 | <0.001 | 0.268, 0.653 | 22 | 0.70 | 6.51 | 0.011 |
|  | GLS | -0.152 | 0.684 | -0.922, 0.618 | 0.348 | <0.001 | 0.180, 0.515 | 22 | - |  |  |
| clutches p.a. | PGLS | - | - | - | - | - | - | 2 | - |  |  |
|  | GLS | - | - | - | - | - | - | 2 | - |  |  |
| egg weigth | PGLS | 0.347 | 0.158 | -0.147, 0.841 | 0.350 | <0.001 | 0.246, 0.453 | 22 | 0.95 | 2.22 | 0.136 |
|  | GLS | 0.609 | 0.010 | 0.177, 1.042 | 0.295 | <0.001 | 0.201, 0.389 | 22 | - |  |  |
| female maturity | PGLS | 2.825 | <0.001 | 1.934, 3.716 | 0.154 | 0.110 | -0.039, 0.347 | 17 | -0.10 | 0.35 | 0.56 |
|  | GLS | 2.756 | <0.001 | 1.725, 3.786 | 0.171 | 0.130 | -0.057, 0.398 | 17 | - |  |  |
| incubation time | PGLS | 2.198 | <0.001 | 1.715, 2.681 | -0.064 | 0.203 | -0.166, 0.038 | 22 | 0.93 | 5.47 | 0.019 |
|  | GLS | 2.331 | <0.001 | 1.924, 2.739 | -0.093 | 0.041 | -0.182, -0.004 | 22 | - |  |  |
| max. longevity | PGLS | 1.683 | 0.006 | 0.553, 2.813 | -0.026 | 0.822 | -0.267, 0.214 | 21 | 0.87 | 0.07 | 0.79 |
|  | GLS | 1.513 | 0.006 | 0.490, 2.535 | -0.003 | 0.980 | -0.225, 0.220 | 21 | - |  |  |
| size at maturity | PGLS | 1.053 | <0.001 | 0.662, 1.444 | 0.273 | <0.001 | 0.192, 0.355 | 11 | -0.28 | 5.73 | 0.017 |
|  | GLS | 0.868 | <0.001 | 0.302, 1.433 | 0.309 | <0.001 | 0.190, 0.429 | 11 | - |  |  |
|  |  |  |  |  |  |  |  |  |  |  |  |

Table S6 Allometric relationships of life history traits of the turtles. Relationships are calculated by generalized least squares (GLS) regression and phylogenetically-informed GLS regression (PGLS) analysis of log_10_-log_10_-transformed data with adult weight as the independent variable. Phylogeny taken from Guillon et al. (2012). df = degree of freedom. CI_i_ = confidence interval of intercept, CI_s_ = confidence interval of slope, λ = Pagels’ lambda as measure of phylogenetic impact (Pagel 1991). The significance of λ is assessed from likelihood ratio tests between model types GLS and PGLS: Χ^2^ = Chi-squared statistic; P(Χ^2^) = corresponding p-value.

| Trait | Statistics | Intercept | p-value | CI_i_ | Slope | p-value | CI_s_ | df | λ | Χ^2^ | P(Χ^2^) |
| --- | --- | --- | --- | --- | --- | --- | --- | --- | --- | --- | --- |
|  |  |  |  |  |  |  |  |  |  |  |  |
| birth size TL | PGLS | 0.205 | 0.044 | 0.007, 0.405 | 0.098 | <0.001 | 0.069, 0.128 | 47 | 0.90 | 18.70 | <0.001 |
|  | GLS | 0.323 | <0.001 | 0.216, 0.430 | 0.076 | <0.001 | 0.048, 0.104 | 47 | - |  |  |
| birth weight | PGLS | 0.347 | 0.225 | -0.226, 0.920 | 0.207 | <0.001 | 0.130, 0.284 | 30 | 0.97 | 17.10 | <0.001 |
|  | GLS | 0.600 | 0.002 | 0.250, 0.949 | 0.176 | <0.001 | 0.085, 0.267 | 30 | - |  |  |
| clutch size | PGLS | 0.315 | 0.391 | -0.417, 1.047 | 0.311 | <0.001 | 0.214, 0.408 | 49 | 0.94 | 31.65 | <0.001 |
|  | GLS | -0.844 | <0.001 | -1.258, -0.430 | 0.498 | <0.001 | 0.387, 0.609 | 49 | - |  |  |
| clutches p.a. | PGLS | 0.242 | 0.067 | -0.018, 0.501 | 0.054 | 0.150 | -0.021, 0.129 | 33 | -0.16 | 1.19 | 0.27 |
|  | GLS | 0.160 | 0.318 | -0.161, 0.481 | 0.056 | 0.190 | -0.029, 0.142 | 33 | - |  |  |
| egg weigth | PGLS | 0.737 | 0.056 | -0.023, 1.497 | 0.136 | 0.082 | -0.022, 0.293 | 10 | 1.09 | 3.21 | 0.07 |
|  | GLS | 0.381 | 0.240 | -0.311, 1.072 | 0.232 | 0.012 | 0.067, 0.397 | 10 | - |  |  |
| female maturity | PGLS | 3.315 | <0.001 | 2.982, 3.647 | 0.048 | 0.235 | -0.034, 0.131 | 22 | 0.05 | 0.04 | 0.85 |
|  | GLS | 3.362 | <0.001 | 3.039, 3.684 | 0.037 | 0.365 | -0.047, 0.121 | 22 | - |  |  |
| incubation time | PGLS | 1.826 | <0.001 | 1.374, 2.279 | -0.005 | 0.895 | -0.065, 0.056 | 46 | 0.95 | 26.25 | <0.001 |
|  | GLS | 2.253 | <0.001 | 1.991, 2.514 | -0.061 | 0.083 | -0.131, 0.008 | 46 | - |  |  |
| max. longevity | PGLS | 1.246 | <0.001 | 0.762, 1.730 | 0.077 | 0.131 | -0.023, 0.177 | 28 | 0.32 | 1.99 | 0.158 |
|  | GLS | 1.351 | <0.001 | 0.986, 1.717 | 0.073 | 0.122 | -0.020, 1.68 | 28 | - |  |  |
| size at maturity | PGLS | 0.809 | 0.003 | 0.312, 1.306 | 0.210 | <0.001 | 0.107, 0.312 | 17 | 1.02 | 5.25 | 0.02 |
|  | GLS | 0.219 | 0.270 | -0.188, 0.627 | 0.328 | <0.001 | 0.226, 0.430 | 17 | - |  |  |
|  |  |  |  |  |  |  |  |  |  |  |  |

Table S7 Allometric relationships of life-history traits for the order Squamata (lizards, snakes, and amphisbaenians). Relationships are calculated by generalized least squares (GLS) regression and phylogenetically-informed GLS regression (PGLS) analysis of log_10_-log_10_-transformed data with adult weight as the independent variable. Phylogeny from Pyron & Burbrink (2014). df = degree of freedom. CI_i_ = confidence interval of intercept, CI_s_ = confidence interval of slope, λ = Pagels’ lambda as measure of phylogenetic impact (Pagel 1991). Significance of λ is assessed from likelihood ratio tests between model types GLS and PGLS: Χ^2^ = Chi-squared statistic; P(Χ^2^) = corresponding p-value.

| Trait | Statistics | Intercept | p-value | CI_i_ | Slope | p-value | CI_s_ | df | λ | Χ^2^ | P(Χ^2^) |
| --- | --- | --- | --- | --- | --- | --- | --- | --- | --- | --- | --- |
|  |  |  |  |  |  |  |  |  |  |  |  |
| birth size TL | PGLS | 0.645 | <0.001 | 0.483, 0.806 | 0.191 | <0.001 | 0.128, 0.254 | 91 | 0.39 | 4.78 | 0.03 |
|  | GLS | 0.598 | <0.001 | 0.510, 0.687 | 0.248 | <0.001 | 0.199, 0.297 | 92 | - |  |  |
| birth weight | PGLS | -0.696 | <0.001 | -0.959, -0.433 | 0.626 | <0.001 | 0.488, 0.764 | 42 | -0.06 | 0.39 | 0.53 |
|  | GLS | -0.657 | <0.001 | -0.952, -0.363 | 0.594 | <0.001 | 0.440, 0.748 | 43 | - |  |  |
| clutch size | PGLS | 0.404 | <0.001 | 0.190, 0.617 | 0.179 | <0.001 | 0.116, 0.243 | 137 | 0.68 | 41.97 | <0.001 |
|  | GLS | 0.337 | <0.001 | 0.232, 0.441 | 0.257 | <0.001 | 0.206, 0.309 | 140 | - |  |  |
| clutches p.a. | PGLS | 0.189 | 0.047 | 0.002, 0.375 | -0.009 | 0.810 | -0.086, 0.067 | 99 | 0.63 | 36.71 | <0.001 |
|  | GLS | 0.393 | <0.001 | 0.286, 0.500 | -0.146 | <0.001 | -0.215, -0.078 | 101 | - |  |  |
| egg weigth | PGLS | -0.636 | <0.001 | -0.944, -0.328 | 0.587 | <0.001 | 0.457, 0.717 | 29 | 0.70 | 8.63 | 0.003 |
|  | GLS | -0.753 | <0.001 | -0.949, -0.556 | 0.665 | <0.001 | 0.561, 0.769 | 29 | - |  |  |
| female maturity | PGLS | 2.735 | <0.001 | 2.613, 2.860 | 0.090 | <0.001 | 0.045, 0.135 | 80 | 0.41 | 6.46 | 0.011 |
|  | GLS | 2.663 | <0.001 | 2.583, 2.742 | 0.134 | <0.001 | 0.097, 0.172 | 81 | - |  |  |
| incubation time | PGLS | 1.738 | <0.001 | 1.596, 1.880 | 0.063 | 0.006 | 0.018, 0.107 | 106 | 0.73 | 11.31 | <0.001 |
|  | GLS | 1.750 | <0.001 | 1.690, 1.810 | 0.054 | 0.001 | 0.025, 0.083 | 107 | - |  |  |
| max. longevity | PGLS | 0.877 | <0.001 | 0.733, 1.021 | 0.121 | <0.001 | 0.086, 0.156 | 225 | 0.77 | 63.00 | <0.001 |
|  | GLS | 0.886 | <0.001 | 0.820, 0.952 | 0.121 | <0.001 | 0.093, 0.149 | 230 | - |  |  |
| size at maturity | PGLS | 0.741 | <0.001 | 0.392, 1.091 | 0.262 | <0.001 | 0.127, 0.397 | 25 | 0.97 | 10.68 | 0.001 |
|  | GLS | 0.239 | 0.047 | 0.003, 0.475 | 0.614 | <0.001 | 0.498, 0.731 | 25 | - |  |  |
|  |  |  |  |  |  |  |  |  |  |  |  |

Table S8 Allometric relationships of life-history traits for Squamata clade Gekkota. Relationships are calculated by generalized least squares (GLS) regression and phylogenetically-informed GLS regression (PGLS) analysis of log_10_-log_10_-transformed data with adult weight as the independent variable. Phylogeny taken from Pyron & Burbrink (2014). df = degree of freedom, CI_i_ = confidence interval of intercept, CI_s_ = confidence interval of slope, λ = Pagels’ lambda as measure of phylogenetic impact (Pagel 1991).

| Trait | Statistics | Intercept | P-value | 95%CI_i_ | Slope | P-value | 95%CI_s_ | df | λ |
| --- | --- | --- | --- | --- | --- | --- | --- | --- | --- |
|  |  |  |  |  |  |  |  |  |  |
| birth size TL | PGLS | 0.578 | <0.001 | 0.424, 0.733 | 0.187 | 0.08 | -0.021, 0.395 | 28 | -0.15 |
|  | GLS | 0.581 | <0.001 | 0.379, 0.783 | 0.190 | 0.09 | -0.028, 0.408 | 28 | - |
| birth weight | PGLS | -0.803 | <0.001 | -1.086, -0.520 | 0.683 | <0.001 | 0.432, 0.934 | 12 | 0.91 |
|  | GLS | -0.853 | <0.001 | -1.075, -0.632 | 0.671 | <0.001 | 0.427, 0.915 | 12 | - |
| clutch size | PGLS | 0.143 | 0.145 | -0.052, 0.339 | 0.164 | 0.11 | -0.037, 0.365 | 28 | 0.08 |
|  | GLS | 0.133 | 0.144 | -0.048, 0.314 | 0.171 | 0.088 | -0.027, 0.369 | 28 | - |
| clutches p.a. | PGLS | 0.596 | <0.001 | 0.274, 0.919 | -0.005 | 0.977 | -0.329, 0.320 | 25 | 0.23 |
|  | GLS | 0.541 | <0.001 | 0.276, 0.807 | 0.013 | 0.928 | -0.291, 0.318 | 25 | - |
| egg weight | PGLS | -0.498 | 0.010 | -0.843, -0.154 | 0.420 | 0.029 | 0.057, 0.783 | 10 | 0.98 |
|  | GLS | -0.708 | <0.001 | -0.979, -0.437 | 0.680 | 0.001 | 0.351, 1.009 | 10 | - |
| female maturity | PGLS | 2.824 | <0.001 | 2.533, 3.116 | -0.118 | 0.17 | -0.295, 0.059 | 12 | 1.73 |
|  | GLS | 2.760 | <0.001 | 2.498, 3.022 | -0.091 | 0.473 | -0.363, 0.181 | 12 | - |
| incubation time | PGLS | 1.731 | <0.001 | 1.596, 1.867 | 0.109 | 0.028 | 0.013, 0.205 | 29 | 0.82 |
|  | GLS | 1.773 | <0.001 | 1.682, 1.865 | 0.070 | 0.158 | -0.029, 0.168 | 29 | - |
| max. longevity | PGLS | 0.833 | <0.001 | 0.682, 0.983 | 0.261 | <0.001 | 0.122, 0.399 | 27 | 0.02 |
|  | GLS | 0.830 | <0.001 | 0.682, 0.977 | 0.263 | <0.001 | 0.125, 0.401 | 27 | - |
| size at maturity | PGLS | - | - | - | - | - | - | 3 | - |
|  | GLS | - | - | - | - | - | - | 3 | - |

Table S9 Allometric relationships of life-history traits for Squamata clade Scincomorpha. Relationships are calculated by generalized least squares (GLS) regression and phylogenetically-informed GLS regression (PGLS) analysis of log_10_-log_10_-transformed data with adult weight as the independent variable. Phylogeny taken from Pyron & Burbrink (2014). df = degree of freedom, CI_i_ = confidence interval of intercept, CI_s_ = confidence interval of slope, λ = Pagels’ lambda as measure of phylogenetic impact (Pagel 1991).

| Trait | Statistics | Intercept | P-value | 95%CI_i_ | Slope | P-value | 95%CI_s_ | df | λ |
| --- | --- | --- | --- | --- | --- | --- | --- | --- | --- |
|  |  |  |  |  |  |  |  |  |  |
| birth size TL | PGLS | - | - | - | - | - | - | 6 | - |
|  | GLS | - | - | - | - | - | - | 6 | - |
| birth weight | PGLS | - | - | - | - | - | - | 3 | - |
|  | GLS | - | - | - | - | - | - | 3 | - |
| clutch size | PGLS | 0.755 | <0.001 | 0.506, 1.003 | -0.042 | 0.637 | -0.232, 0.149 | 12 | -0.27 |
|  | GLS | 0.706 | 0.002 | 0.332, 1.080 | -0.032 | 0.726 | -0.233, 0.168 | 12 | - |
| clutches p.a. | PGLS | 0.027 | 0.643 | -0.109, 0.163 | -0.068 | 0.023 | -0.123, -0.013 | 8 | 1.15 |
|  | GLS | 0.069 | 0.185 | -0.044, 0.183 | -0.086 | 0.030 | -0.160, -0.011 | 8 | - |
| egg weight | PGLS | - | - | - | - | - | - | - | - |
|  | GLS | - | - | - | - | - | - | - | - |
| female maturity | PGLS | - | - | - | - | - | - | 4 | - |
|  | GLS | - | - | - | - | - | - | 4 | - |
| incubation time | PGLS | - | - | - | - | - | - | 5 | - |
|  | GLS | - | - | - | - | - | - | 5 | - |
| max. longevity | PGLS | 0.874 | <0.001 | 0.661, 1.087 | 0.167 | 0.003 | 0.064, 0.269 | 19 | 0.06 |
|  | GLS | 0.877 | <0.001 | 0.669, 1.086 | 0.164 | 0.003 | 0.062, 0.265 | 19 | - |
| size at maturity | PGLS | - | - | - | - | - | - | - | - |
|  | GLS | - | - | - | - | - | - | - | - |

Table S10 Allometric relationships of life-history traits for Squamata clade Lacertoidea (including Amphisbaenia). Relationships are calculated by generalized least squares (GLS) regression and phylogenetically-informed GLS regression (PGLS) analysis of log_10_-log_10_-transformed data with adult weight as the independent variable. Phylogeny from Pyron & Burbrink (2014). df = degree of freedom, CI_i_ = confidence interval of intercept, CI_s_ = confidence interval of slope, λ = Pagels’ lambda as measure of phylogenetic impact (Pagel 1991).

| Trait | Statistics | Intercept | P-value | 95%CI_i_ | Slope | P-value | 95%CI_s_ | df | λ |
| --- | --- | --- | --- | --- | --- | --- | --- | --- | --- |
|  |  |  |  |  |  |  |  |  |  |
| birth size TL | PGLS | - | - | - | - | - | - | - | - |
|  | GLS | 0.838 | <0.001 | 0.673, 1.003 | 0.047 | 0.448 | -0.079, 0.172 | 24 | - |
| birth weight | PGLS | - | - | - | - | - | - | - | - |
|  | GLS | -0.277 | 0.746 | -0.853, 1.566 | 0.357 | 0.516 | -0.853, 1.566 | 10 | - |
| clutch size | PGLS | - | - | - | - | - | - | - | - |
|  | GLS | 0.556 | <0.001 | 0.334, 0.777 | 0.147 | <0.05 | 0.002, 0.292 | 30 | - |
| clutches p.a. | PGLS | - | - | - | - | - | - | - | - |
|  | GLS | 0.135 | 0.032 | 0.012, 0.257 | 0.016 | 0.740 | -0.082, 0.114 | 30 | - |
| egg weigth | PGLS | - | - | - | - | - | - | - | - |
|  | GLS | -1.279 | <0.001 | -1.593, -0.966 | 0.822 | <0.001 | 0.662, 0.982 | 8 | - |
| female maturity | PGLS | - | - | - | - | - | - | - | - |
|  | GLS | 2.553 | <0.001 | 2.380, 2.726 | 0.187 | 0.006 | 0.064, 0.311 | 16 | - |
| incubation time | PGLS | - | - | - | - | - | - | - | - |
|  | GLS | 1.662 | <0.001 | 1.531, 1.794 | 0.088 | <0.05 | 0.002, 0.174 | 24 | - |
| max. longevity | PGLS | - | - | - | - | - | - | - | - |
|  | GLS | 0.938 | <0.001 | 0.720, 1.156 | 0.030 | 0.55 | -0.076, 0.136 | 18 | - |
| size at maturity | PGLS | - | - | - | - | - | - | - | - |
|  | GLS | - | - | - | - | - | - | 6 | - |
|  |  |  |  |  |  |  |  |  |  |

Table S11 Allometric relationships of life history traits for Squamata clade Iguania. Relationships are calculated by generalized least squares (GLS) regression and phylogenetically-informed GLS regression (PGLS) analysis of log_10_-log_10_-transformed data with adult weight as the independent variable. Phylogeny taken from Pyron & Burbrink (2014). df = degree of freedom. CI_i_ = confidence interval of intercept, CI_s_ = confidence interval of slope, λ = Pagels’ lambda as measure of phylogenetic impact (Pagel 1991).

| Trait | Statistics | Intercept | P-value | CI_i_ | Slope | P-value | CI_s_ | df | λ |
| --- | --- | --- | --- | --- | --- | --- | --- | --- | --- |
|  |  |  |  |  |  |  |  |  |  |
| birth size TL | PGLS | - | - | - | - | - | - | 6 | - |
|  | GLS | - | - | - | - | - | - | 6 | - |
| birth weight | PGLS | - | - | - | - | - | - | 4 | - |
|  | GLS | - | - | - | - | - | - | 4 | - |
| clutch size | PGLS | -0.655 | 0.142 | -1.547, 0.238 | 0.461 | <0.001 | 0.268, 0.653 | 22 | 0.70 |
|  | GLS | -0.152 | 0.684 | -0.922, 0.618 | 0.348 | <0.001 | 0.180, 0.515 | 22 | - |
| clutches p.a. | PGLS | - | - | - | - | - | - | 2 | - |
|  | GLS | - | - | - | - | - | - | 2 | - |
| egg weigth | PGLS | 0.347 | 0.158 | -0.147, 0.841 | 0.350 | <0.001 | 0.246, 0.453 | 22 | 0.95 |
|  | GLS | 0.609 | 0.010 | 0.177, 1.042 | 0.295 | <0.001 | 0.201, 0.389 | 22 | - |
| female maturity | PGLS | 2.825 | <0.001 | 1.934, 3.716 | 0.154 | 0.110 | -0.039, 0.347 | 17 | -0.10 |
|  | GLS | 2.756 | <0.001 | 1.725, 3.786 | 0.171 | 0.130 | -0.057, 0.398 | 17 | - |
| incubation time | PGLS | 2.198 | <0.001 | 1.715, 2.681 | -0.064 | 0.203 | -0.166, 0.038 | 22 | 0.93 |
|  | GLS | 2.331 | <0.001 | 1.924, 2.739 | -0.093 | 0.041 | -0.182, -0.004 | 22 | - |
| max. longevity | PGLS | 1.683 | 0.006 | 0.553, 2.813 | -0.026 | 0.822 | -0.267, 0.214 | 21 | 0.87 |
|  | GLS | 1.513 | 0.006 | 0.490, 2.535 | -0.003 | 0.980 | -0.225, 0.220 | 21 | - |
| size at maturity | PGLS | 1.053 | <0.001 | 0.662, 1.444 | 0.273 | <0.001 | 0.192, 0.355 | 11 | -0.28 |
|  | GLS | 0.868 | <0.001 | 0.302, 1.433 | 0.309 | <0.001 | 0.190, 0.429 | 11 | - |
|  |  |  |  |  |  |  |  |  |  |

Table S12 Allometric relationships of life history traits for Squamata clade Anguimorpha. Relationships are calculated by generalized least squares (GLS) regression and phylogenetically-informed GLS regression (PGLS) analysis of log_10_-log_10_-transformed data with adult weight as the independent variable. Phylogeny taken from Guillon et al. (2012). df = degree of freedom. CI_i_ = confidence interval of intercept, CI_s_ = confidence interval of slope, λ = Pagels’ lambda as measure of phylogenetic impact (Pagel 1991).

| Trait | Statistics | Intercept | p-value | CI_i_ | Slope | p-value | CI_s_ | df | λ |
| --- | --- | --- | --- | --- | --- | --- | --- | --- | --- |
|  |  |  |  |  |  |  |  |  |  |
| birth size TL | PGLS | - | - | - | - | - | - | - | - |
|  | GLS | - | - | - | - | - | - | - | - |
| birth weight | PGLS | - | - | - | - | - | - | - | - |
|  | GLS | - | - | - | - | - | - | - | - |
| clutch size | PGLS | - | - | - | - | - | - | 6 | - |
|  | GLS | - | - | - | - | - | - | 6 | - |
| clutches p.a. | PGLS | - | - | - | - | - | - | - | - |
|  | GLS | - | - | - | - | - | - | - | - |
| egg weigth | PGLS | - | - | - | - | - | - | - | - |
|  | GLS | - | - | - | - | - | - | - | - |
| female maturity | PGLS | - | - | - | - | - | - | 3 | - |
|  | GLS | - | - | - | - | - | - | 3 | - |
| incubation time | PGLS | - | - | - | - | - | - | - | - |
|  | GLS | - | - | - | - | - | - | 5 | - |
| max. longevity | PGLS | 1.064 | <0.001 | 0.734, 1.394 | 0.079 | <0.05 | 0.002, 0.157 | 22 | 1.02 |
|  | GLS | 1.280 | <0.001 | 0.997, 1.563 | -0.009 | 0.84 | -0.100, 0.082 | 22 | - |
| size at maturity | PGLS | - | - | - | - | - | - | - | - |
|  | GLS | - | - | - | - | - | - | - | - |
|  |  |  |  |  |  |  |  |  |  |

Table S13 Tests on different trait evolution models conducted in order to identify the one working best for all six studied reptile groups. The phylogenetic signal was quantified in the trait adult weight by establishing intercept-only models by GLS assuming different phylogenetic correlation structures (Paradis 2011). The GLS models were then compared across all models based on their respective Akaike Information criterion (AIC) values. ∆AIC scores are shown as the difference between the best model (smallest AIC) and each model (so the best model has a ∆AIC of zero). Null = no correlation structure specified; BM = Brownian motion model (Felsenstein 1985)*; Martins = covariance matrix as deﬁned in Martins and Hansen (1997)*; Grafen = covariance matrix as deﬁned in Grafen (1989)*; Pagel = covariance matrix as deﬁned in Freckelton et al. (2002)*; Blomberg = covariance matrix as deﬁned in Blomberg et al. (2003)*. Based on AIC model selection Pagel’s correlation structure was either the best model (lowest AIC value) for all six taxa, it revealed a model not distinguishable from the best model (∆AIC<2), or revealed a model that was only moderately better (∆AIC≤10) than the best model. *Felsenstein J. (1985): Phylogenies and the comparative method. The American Naturalist. 125:1-15; Martins E., Hansen T. (1997): Phylogenies and the comparative method: a general approach to incorporating phylogenetic information into the analysis of interspecific data. The American Naturalist. 149:646-667; Grafen (1989): The phylogenetic regression. Philosophical transactions of the Royal Society of London. Series B, Biological sciences. 326:119-157; Freckleton R.P., Harvey P.H., Pagel M. (2002): Phylogenetic analysis and comparative data: a test and review of evidence. The American Naturalist. 160:712-726.;

Blomberg S. P., Garland Th., Ives A. R. (2003): Testing for phylogenetic signal in comparative data: behavioral traits are more labile. Evolution: International Journal of Organic Evolution. 57:717-745.

| Correlation structures |  | Reptilia (without *Sphenodon punctatus*) |  | Squamata | Lizards | Snakes | Crocodilia | Testudines |
| --- | --- | --- | --- | --- | --- | --- | --- | --- |
|  |  |  |  |  |  |  |  |  |
| Null | AIC | 8611.637 |  | 5976.36 | 3443.457 | 2434.78 | 510.986 | 1312.568 |
|  | (∆AIC) | (-127.61) |  | (-4.069) | (-2.037) | (-1.964) | (-8.339) | (-14.958) |
| BM | AIC | 8519.064 |  | 6190.323 | 3607.196 | 2470.093 | 502.647 | 1302.144 |
|  | (∆AIC) | (-35.037) |  | (-218.032) | (-165.78) | (-37.277) | (0) | (-4.534) |
| Pagel (λ) | AIC | 8492.276 |  | 5974.351 | 3442.266 | 2432.816 | 504.549 | 1297.61 |
|  | (∆AIC) | (-8.249) |  | (-2.06) | (-0.846) | (0) | (-1.902) | (0) |
| Martins (α) | AIC | 8594.655 |  | 5974.456 | 3442.751 | 2433.804 | 509.132 | 1310.68 |
|  | (∆AIC) | (-110.628) |  | (-2.165) | (-1.3351) | (-0.988) | (-6.485) | (-13.07) |
| Grafen (ρ) | AIC | 8484.027 |  | 5972.291 | 3441.42 | 2433.687 | 502.944 | 1303.031 |
|  | (∆AIC) | (0) |  | (0) | (0) | (-0.871) | (-0.297) | (-5.421) |
| Blomberg (κ) | AIC | 8519.064 |  | 6190.323 | 3607.196 | 2470.093 | 502.647 | 1302.144 |
|  | (∆AIC) | (-35.037) |  | (-218.032) | (-165.78) | (-37.277) | (0) | (-4.534) |
|  |  |  |  |  |  |  |  |  |
