# Supplementary material for: An exploration of differences in the scaling of life history traits with body mass within reptiles and between amniotes
Source: Ecol Evol. 2018 May 2;8(11):5480–94. doi: 10.1002/ece3.4069 (PMC6010814; doi:10.1002/ece3.4069)
Supplement: Supplementary file 2 [file ECE3-8-5480-s002.docx]

Table S14. Allometric relationships on life history traits, maximum altitude and adult body mass in different taxa (R output). Tests whether maximum altitude affects allometric scaling of life history traits.

Crocodilia-nonpgls

******************

clutch_size

--------------------------------

Generalized least squares fit by REML

Model: log10(trait2) ~ log10(max_altitude) + log10(adult_weight)

Data: DF.rep_tree

AIC BIC logLik

3.114405 1.552156 2.442798

Coefficients:

Value Std.Error t-value p-value

(Intercept) 0.3759631 0.5515918 0.6815967 0.5258

log10(max_altitude) 0.0318142 0.0770712 0.4127897 0.6969

log10(adult_weight) 0.2233594 0.0959920 2.3268546 0.0675

Correlation:

(Intr) lg10(m_)

log10(max_altitude) -0.727

log10(adult_weight) -0.947 0.477

Standardized residuals:

Min Q1 Med Q3 Max

-1.50335176 -0.43021041 -0.02977472 0.51872770 1.11095582

Residual standard error: 0.1074831

Degrees of freedom: 8 total; 5 residual

egg_weight

--------------------------------

Generalized least squares fit by REML

Model: log10(trait2) ~ log10(max_altitude) + log10(adult_weight)

Data: DF.rep_tree

AIC BIC logLik

-0.2231757 -1.785424 4.111588

Coefficients:

Value Std.Error t-value p-value

(Intercept) 0.2865926 0.3950650 0.725432 0.5007

log10(max_altitude) 0.0513145 0.0552005 0.929603 0.3952

log10(adult_weight) 0.3379186 0.0687521 4.915033 0.0044

Correlation:

(Intr) lg10(m_)

log10(max_altitude) -0.727

log10(adult_weight) -0.947 0.477

Standardized residuals:

Min Q1 Med Q3 Max

-1.472896258 -0.434074915 -0.004588171 0.676461111 1.004665945

Residual standard error: 0.07698234

Degrees of freedom: 8 total; 5 residual

female_maturity

--------------------------------

Generalized least squares fit by REML

Model: log10(trait2) ~ log10(max_altitude) + log10(adult_weight)

Data: DF.rep_tree

AIC BIC logLik

12.07887 9.62405 -2.039436

Coefficients:

Value Std.Error t-value p-value

(Intercept) 2.6308580 1.4541046 1.8092633 0.1447

log10(max_altitude) -0.0364246 0.2037427 -0.1787772 0.8668

log10(adult_weight) 0.2129995 0.2524693 0.8436652 0.4464

Correlation:

(Intr) lg10(m_)

log10(max_altitude) -0.742

log10(adult_weight) -0.950 0.504

Standardized residuals:

Min Q1 Med Q3 Max

-0.8314009 -0.5949432 -0.1617098 0.3750288 1.4329396

Residual standard error: 0.2760506

Degrees of freedom: 7 total; 4 residual

incubation_time

--------------------------------

Generalized least squares fit by REML

Model: log10(trait2) ~ log10(max_altitude) + log10(adult_weight)

Data: DF.rep_tree

AIC BIC logLik

1.752318 0.1900695 3.123841

Coefficients:

Value Std.Error t-value p-value

(Intercept) 2.0961979 0.4813524 4.354810 0.0073

log10(max_altitude) -0.0215820 0.0672570 -0.320888 0.7613

log10(adult_weight) -0.0271058 0.0837684 -0.323581 0.7594

Correlation:

(Intr) lg10(m_)

log10(max_altitude) -0.727

log10(adult_weight) -0.947 0.477

Standardized residuals:

Min Q1 Med Q3 Max

-1.0134476 -0.5999700 -0.1207373 0.4375425 1.4578590

Residual standard error: 0.0937963

Degrees of freedom: 8 total; 5 residual

max_longevity

--------------------------------

Generalized least squares fit by REML

Model: log10(trait2) ~ log10(max_altitude) + log10(adult_weight)

Data: DF.rep_tree

AIC BIC logLik

6.425678 4.863429 0.7871612

Coefficients:

Value Std.Error t-value p-value

(Intercept) 2.2246143 0.7681120 2.8962108 0.0339

log10(max_altitude) -0.0975577 0.1073245 -0.9089977 0.4050

log10(adult_weight) -0.1138447 0.1336724 -0.8516699 0.4333

Correlation:

(Intr) lg10(m_)

log10(max_altitude) -0.727

log10(adult_weight) -0.947 0.477

Standardized residuals:

Min Q1 Med Q3 Max

-0.91580159 -0.71218610 -0.09542041 0.53712863 1.52183444

Residual standard error: 0.1496743

Degrees of freedom: 8 total; 5 residual

size_at_maturity

--------------------------------

Generalized least squares fit by REML

Model: log10(trait2) ~ log10(max_altitude) + log10(adult_weight)

Data: DF.rep_tree

AIC BIC logLik

-1.670806 -6.898217 4.835403

Coefficients:

Value Std.Error t-value p-value

(Intercept) 1.0404654 0.10036193 10.367132 0.0092

log10(max_altitude) 0.0105770 0.01202400 0.879654 0.4718

log10(adult_weight) 0.2727626 0.01675634 16.278173 0.0038

Correlation:

(Intr) lg10(m_)

log10(max_altitude) -0.789

log10(adult_weight) -0.969 0.621

Standardized residuals:

93 94 157 245 330

-0.970714586 1.022333272 -0.099685759 -0.002942379 0.051009452

attr(,"std")

[1] 0.01346372 0.01346372 0.01346372 0.01346372 0.01346372

attr(,"label")

[1] "Standardized residuals"

Residual standard error: 0.01346372

Degrees of freedom: 5 total; 2 residual

***************************************

Crocodilia-pgls

***************

birth_size_wt

--------------------------------

Generalized least squares fit by REML

Model: log10(trait2) ~ log10(max_altitude) + log10(adult_weight)

Data: DF.rep_tree

AIC BIC logLik

-0.4412305 -6.975495 5.220615

Correlation Structure: corPagel

Formula: ~1

Parameter estimate(s):

lambda

-1.106307

Coefficients:

Value Std.Error t-value p-value

(Intercept) 1.0094617 0.11518628 8.763732 0.0128

log10(max_altitude) 0.0153488 0.02160944 0.710283 0.5512

log10(adult_weight) 0.2766998 0.01448961 19.096423 0.0027

Correlation:

(Intr) lg10(m_)

log10(max_altitude) -0.896

log10(adult_weight) -0.919 0.649

Standardized residuals:

Crocodylus_palustris Crocodylus_novaeguineae Tomistoma_schlegelii Gavialis_gangeticus Paleosuchus_trigonatus

1.36069421 -1.41069911 0.57730271 -0.37395224 0.08903896

attr(,"std")

[1] 0.009525322 0.009525322 0.009525322 0.009525322 0.009525322

attr(,"label")

[1] "Standardized residuals"

Residual standard error: 0.009525322

Degrees of freedom: 5 total; 2 residual

birth_weight

--------------------------------

Generalized least squares fit by REML

Model: log10(trait2) ~ log10(max_altitude) + log10(adult_weight)

Data: DF.rep_tree

AIC BIC logLik

-0.4412305 -6.975495 5.220615

Correlation Structure: corPagel

Formula: ~1

Parameter estimate(s):

lambda

-1.106307

Coefficients:

Value Std.Error t-value p-value

(Intercept) 1.0094617 0.11518628 8.763732 0.0128

log10(max_altitude) 0.0153488 0.02160944 0.710283 0.5512

log10(adult_weight) 0.2766998 0.01448961 19.096423 0.0027

Correlation:

(Intr) lg10(m_)

log10(max_altitude) -0.896

log10(adult_weight) -0.919 0.649

Standardized residuals:

Crocodylus_palustris Crocodylus_novaeguineae Tomistoma_schlegelii Gavialis_gangeticus Paleosuchus_trigonatus

1.36069421 -1.41069911 0.57730271 -0.37395224 0.08903896

attr(,"std")

[1] 0.009525322 0.009525322 0.009525322 0.009525322 0.009525322

attr(,"label")

[1] "Standardized residuals"

Residual standard error: 0.009525322

Degrees of freedom: 5 total; 2 residual

clutch_size

--------------------------------

Generalized least squares fit by REML

Model: log10(trait2) ~ log10(max_altitude) + log10(adult_weight)

Data: DF.rep_tree

AIC BIC logLik

4.881249 2.928438 2.559376

Correlation Structure: corPagel

Formula: ~1

Parameter estimate(s):

lambda

-0.6019073

Coefficients:

Value Std.Error t-value p-value

(Intercept) 0.3114763 0.3538904 0.880149 0.4191

log10(max_altitude) 0.0390368 0.0733004 0.532559 0.6171

log10(adult_weight) 0.2334744 0.0383441 6.088924 0.0017

Correlation:

(Intr) lg10(m_)

log10(max_altitude) -0.985

log10(adult_weight) -0.974 0.921

Standardized residuals:

Min Q1 Med Q3 Max

-1.69270655 -0.49909596 -0.03111942 0.66380076 1.24203242

Residual standard error: 0.09380568

Degrees of freedom: 8 total; 5 residual

clutches_pa

--------------------------------

Generalized least squares fit by REML

Model: log10(trait2) ~ log10(max_altitude) + log10(adult_weight)

Data: DF.rep_tree

AIC BIC logLik

4.881249 2.928438 2.559376

Correlation Structure: corPagel

Formula: ~1

Parameter estimate(s):

lambda

-0.6019073

Coefficients:

Value Std.Error t-value p-value

(Intercept) 0.3114763 0.3538904 0.880149 0.4191

log10(max_altitude) 0.0390368 0.0733004 0.532559 0.6171

log10(adult_weight) 0.2334744 0.0383441 6.088924 0.0017

Correlation:

(Intr) lg10(m_)

log10(max_altitude) -0.985

log10(adult_weight) -0.974 0.921

Standardized residuals:

Min Q1 Med Q3 Max

-1.69270655 -0.49909596 -0.03111942 0.66380076 1.24203242

Residual standard error: 0.09380568

Degrees of freedom: 8 total; 5 residual

egg_weight

--------------------------------

Generalized least squares fit by REML

Model: log10(trait2) ~ log10(max_altitude) + log10(adult_weight)

Data: DF.rep_tree

AIC BIC logLik

0.1348937 -1.817917 4.932553

Correlation Structure: corPagel

Formula: ~1

Parameter estimate(s):

lambda

1.010858

Coefficients:

Value Std.Error t-value p-value

(Intercept) 0.3523317 0.5655658 0.6229721 0.5606

log10(max_altitude) 0.0136364 0.0506314 0.2693263 0.7984

log10(adult_weight) 0.3411363 0.1162873 2.9335648 0.0325

Correlation:

(Intr) lg10(m_)

log10(max_altitude) -0.444

log10(adult_weight) -0.971 0.244

Standardized residuals:

Min Q1 Med Q3 Max

-1.0305127 -0.1133549 0.2484864 0.4485504 0.9731782

Residual standard error: 0.1137002

Degrees of freedom: 8 total; 5 residual

female_maturity

--------------------------------

Generalized least squares fit by REML

Model: log10(trait2) ~ log10(max_altitude) + log10(adult_weight)

Data: DF.rep_tree

AIC BIC logLik

12.87101 9.802479 -1.435504

Correlation Structure: corPagel

Formula: ~1

Parameter estimate(s):

lambda

1.099025

Coefficients:

Value Std.Error t-value p-value

(Intercept) 5.634957 1.8602767 3.0290962 0.0388

log10(max_altitude) -0.071177 0.1946158 -0.3657298 0.7331

log10(adult_weight) -0.446818 0.3546261 -1.2599706 0.2762

Correlation:

(Intr) lg10(m_)

log10(max_altitude) -0.622

log10(adult_weight) -0.961 0.429

Standardized residuals:

Min Q1 Med Q3 Max

-0.83568512 -0.60122059 -0.04625574 0.56595988 1.18174659

Residual standard error: 0.4982599

Degrees of freedom: 7 total; 4 residual

incubation_time

--------------------------------

Generalized least squares fit by REML

Model: log10(trait2) ~ log10(max_altitude) + log10(adult_weight)

Data: DF.rep_tree

AIC BIC logLik

3.679038 1.726228 3.160481

Correlation Structure: corPagel

Formula: ~1

Parameter estimate(s):

lambda

1.01626

Coefficients:

Value Std.Error t-value p-value

(Intercept) 2.8891965 0.8114244 3.560648 0.0162

log10(max_altitude) -0.0544837 0.0724404 -0.752118 0.4859

log10(adult_weight) -0.1878842 0.1669317 -1.125515 0.3115

Correlation:

(Intr) lg10(m_)

log10(max_altitude) -0.442

log10(adult_weight) -0.971 0.243

Standardized residuals:

Min Q1 Med Q3 Max

-0.9326899 -0.3891177 0.1901672 0.4755257 0.8918479

Residual standard error: 0.1637359

Degrees of freedom: 8 total; 5 residual

max_altitude

--------------------------------

Generalized least squares fit by REML

Model: log10(trait2) ~ log10(max_altitude) + log10(adult_weight)

Data: DF.rep_tree

AIC BIC logLik

3.679038 1.726228 3.160481

Correlation Structure: corPagel

Formula: ~1

Parameter estimate(s):

lambda

1.01626

Coefficients:

Value Std.Error t-value p-value

(Intercept) 2.8891965 0.8114244 3.560648 0.0162

log10(max_altitude) -0.0544837 0.0724404 -0.752118 0.4859

log10(adult_weight) -0.1878842 0.1669317 -1.125515 0.3115

Correlation:

(Intr) lg10(m_)

log10(max_altitude) -0.442

log10(adult_weight) -0.971 0.243

Standardized residuals:

Min Q1 Med Q3 Max

-0.9326899 -0.3891177 0.1901672 0.4755257 0.8918479

Residual standard error: 0.1637359

Degrees of freedom: 8 total; 5 residual

max_longevity

--------------------------------

Generalized least squares fit by REML

Model: log10(trait2) ~ log10(max_altitude) + log10(adult_weight)

Data: DF.rep_tree

AIC BIC logLik

4.93821 2.985399 2.530895

Correlation Structure: corPagel

Formula: ~1

Parameter estimate(s):

lambda

1.104951

Coefficients:

Value Std.Error t-value p-value

(Intercept) 2.6156201 0.7091263 3.688511 0.0142

log10(max_altitude) 0.0425255 0.1012867 0.419853 0.6920

log10(adult_weight) -0.2700879 0.0997006 -2.708989 0.0423

Correlation:

(Intr) lg10(m_)

log10(max_altitude) -0.977

log10(adult_weight) -0.977 1.000

Standardized residuals:

Min Q1 Med Q3 Max

-1.0049600 -0.8491208 -0.1655525 0.3792263 0.7812760

Residual standard error: 0.2652826

Degrees of freedom: 8 total; 5 residual

size_at_maturity

--------------------------------

Generalized least squares fit by REML

Model: log10(trait2) ~ log10(max_altitude) + log10(adult_weight)

Data: DF.rep_tree

AIC BIC logLik

-0.4412305 -6.975495 5.220615

Correlation Structure: corPagel

Formula: ~1

Parameter estimate(s):

lambda

-1.106307

Coefficients:

Value Std.Error t-value p-value

(Intercept) 1.0094617 0.11518628 8.763732 0.0128

log10(max_altitude) 0.0153488 0.02160944 0.710283 0.5512

log10(adult_weight) 0.2766998 0.01448961 19.096423 0.0027

Correlation:

(Intr) lg10(m_)

log10(max_altitude) -0.896

log10(adult_weight) -0.919 0.649

Standardized residuals:

Crocodylus_palustris Crocodylus_novaeguineae Tomistoma_schlegelii Gavialis_gangeticus Paleosuchus_trigonatus

1.36069421 -1.41069911 0.57730271 -0.37395224 0.08903896

attr(,"std")

[1] 0.009525322 0.009525322 0.009525322 0.009525322 0.009525322

attr(,"label")

[1] "Standardized residuals"

Residual standard error: 0.009525322

Degrees of freedom: 5 total; 2 residual

***************************************

Squamata-nonpgls

****************

birth_size_wt

--------------------------------

Generalized least squares fit by REML

Model: log10(trait2) ~ log10(max_altitude) + log10(adult_weight)

Data: DF.rep_tree

AIC BIC logLik

15.92306 23.80423 -3.96153

Coefficients:

Value Std.Error t-value p-value

(Intercept) -0.2081607 0.6346540 -0.327991 0.7442

log10(max_altitude) 0.2751918 0.2002507 1.374236 0.1752

log10(adult_weight) 0.2112555 0.0449756 4.697118 0.0000

Correlation:

(Intr) lg10(m_)

log10(max_altitude) -0.993

log10(adult_weight) 0.210 -0.315

Standardized residuals:

Min Q1 Med Q3 Max

-3.7701858 -0.4431851 0.1592461 0.4129179 3.0693134

Residual standard error: 0.2420795

Degrees of freedom: 56 total; 53 residual

birth_weight

--------------------------------

Generalized least squares fit by REML

Model: log10(trait2) ~ log10(max_altitude) + log10(adult_weight)

Data: DF.rep_tree

AIC BIC logLik

60.41217 65.44455 -26.20608

Coefficients:

Value Std.Error t-value p-value

(Intercept) -1.5440544 2.2993837 -0.6715079 0.5078

log10(max_altitude) 0.3341508 0.7332074 0.4557384 0.6524

log10(adult_weight) 0.5030500 0.1696966 2.9644079 0.0064

Correlation:

(Intr) lg10(m_)

log10(max_altitude) -0.992

log10(adult_weight) 0.322 -0.432

Standardized residuals:

Min Q1 Med Q3 Max

-0.83706568 -0.47541124 -0.03048876 0.18130050 4.56552305

Residual standard error: 0.5945803

Degrees of freedom: 29 total; 26 residual

clutch_size

--------------------------------

Generalized least squares fit by REML

Model: log10(trait2) ~ log10(max_altitude) + log10(adult_weight)

Data: DF.rep_tree

AIC BIC logLik

4.592037 13.10057 1.703981

Coefficients:

Value Std.Error t-value p-value

(Intercept) -0.6633427 0.5475466 -1.211482 0.2303

log10(max_altitude) 0.3250187 0.1722506 1.886894 0.0639

log10(adult_weight) 0.2649984 0.0368555 7.190196 0.0000

Correlation:

(Intr) lg10(m_)

log10(max_altitude) -0.994

log10(adult_weight) 0.207 -0.304

Standardized residuals:

Min Q1 Med Q3 Max

-2.56883517 -0.58243910 0.05129483 0.60137664 2.39271415

Residual standard error: 0.2201062

Degrees of freedom: 65 total; 62 residual

clutches_pa

--------------------------------

Generalized least squares fit by REML

Model: log10(trait2) ~ log10(max_altitude) + log10(adult_weight)

Data: DF.rep_tree

AIC BIC logLik

-22.18213 -13.48458 15.09106

Coefficients:

Value Std.Error t-value p-value

(Intercept) 0.3376440 0.4314574 0.7825664 0.4367

log10(max_altitude) -0.0446652 0.1361849 -0.3279751 0.7440

log10(adult_weight) -0.0554703 0.0296434 -1.8712495 0.0658

Correlation:

(Intr) lg10(m_)

log10(max_altitude) -0.994

log10(adult_weight) 0.216 -0.312

Standardized residuals:

Min Q1 Med Q3 Max

-1.3341170 -0.7132593 -0.2577553 0.4668759 3.1184160

Residual standard error: 0.1797212

Degrees of freedom: 68 total; 65 residual

egg_weight

--------------------------------

Generalized least squares fit by REML

Model: log10(trait2) ~ log10(max_altitude) + log10(adult_weight)

Data: DF.rep_tree

AIC BIC logLik

15.72449 19.28598 -3.862245

Coefficients:

Value Std.Error t-value p-value

(Intercept) -1.9652816 0.9352191 -2.101413 0.0500

log10(max_altitude) 0.3430271 0.2973592 1.153578 0.2638

log10(adult_weight) 0.6971626 0.0869983 8.013520 0.0000

Correlation:

(Intr) lg10(m_)

log10(max_altitude) -0.983

log10(adult_weight) 0.130 -0.299

Standardized residuals:

Min Q1 Med Q3 Max

-1.62581793 -0.45647222 -0.05447798 0.37356560 2.23070446

Residual standard error: 0.2605071

Degrees of freedom: 21 total; 18 residual

female_maturity

--------------------------------

Generalized least squares fit by REML

Model: log10(trait2) ~ log10(max_altitude) + log10(adult_weight)

Data: DF.rep_tree

AIC BIC logLik

-14.64483 -8.310756 11.32242

Coefficients:

Value Std.Error t-value p-value

(Intercept) 2.4221477 0.4477130 5.410045 0.0000

log10(max_altitude) 0.0799967 0.1403982 0.569785 0.5724

log10(adult_weight) 0.1514560 0.0323686 4.679097 0.0000

Correlation:

(Intr) lg10(m_)

log10(max_altitude) -0.992

log10(adult_weight) 0.133 -0.247

Standardized residuals:

Min Q1 Med Q3 Max

-1.9625135 -0.5979227 0.1541425 0.6306105 2.1673376

Residual standard error: 0.1599019

Degrees of freedom: 39 total; 36 residual

incubation_time

--------------------------------

Generalized least squares fit by REML

Model: log10(trait2) ~ log10(max_altitude) + log10(adult_weight)

Data: DF.rep_tree

AIC BIC logLik

-28.18847 -20.78788 18.09423

Coefficients:

Value Std.Error t-value p-value

(Intercept) 1.9440399 0.4002480 4.857088 0.0000

log10(max_altitude) -0.0348091 0.1264939 -0.275184 0.7844

log10(adult_weight) -0.0072479 0.0282730 -0.256355 0.7988

Correlation:

(Intr) lg10(m_)

log10(max_altitude) -0.993

log10(adult_weight) 0.237 -0.342

Standardized residuals:

Min Q1 Med Q3 Max

-3.72109305 -0.51968501 -0.02800659 0.71040752 2.21961462

Residual standard error: 0.1516098

Degrees of freedom: 50 total; 47 residual

max_longevity

--------------------------------

Generalized least squares fit by REML

Model: log10(trait2) ~ log10(max_altitude) + log10(adult_weight)

Data: DF.rep_tree

AIC BIC logLik

7.862776 13.46757 0.06861181

Coefficients:

Value Std.Error t-value p-value

(Intercept) 0.4570602 0.6204834 0.736620 0.4671

log10(max_altitude) 0.1031003 0.1964821 0.524731 0.6036

log10(adult_weight) 0.1596757 0.0499231 3.198434 0.0033

Correlation:

(Intr) lg10(m_)

log10(max_altitude) -0.990

log10(adult_weight) 0.167 -0.291

Standardized residuals:

Min Q1 Med Q3 Max

-1.789982987 -0.750765176 0.008184935 0.693858327 2.439083155

Residual standard error: 0.2159024

Degrees of freedom: 33 total; 30 residual

size_at_maturity

--------------------------------

Generalized least squares fit by REML

Model: log10(trait2) ~ log10(max_altitude) + log10(adult_weight)

Data: DF.rep_tree

AIC BIC logLik

7.428168 11.4111 0.2859162

Coefficients:

Value Std.Error t-value p-value

(Intercept) -0.4174312 1.0345836 -0.403478 0.6909

log10(max_altitude) 0.2154551 0.3277611 0.657354 0.5184

log10(adult_weight) 0.5856945 0.0680071 8.612253 0.0000

Correlation:

(Intr) lg10(m_)

log10(max_altitude) -0.993

log10(adult_weight) 0.330 -0.436

Standardized residuals:

Min Q1 Med Q3 Max

-1.75408125 -0.84398488 0.07055715 0.71619952 1.60282041

Residual standard error: 0.2118483

Degrees of freedom: 23 total; 20 residual

*****************************************

Squamata-pgls

*************

birth_size_wt

--------------------------------

Generalized least squares fit by REML

Model: log10(trait2) ~ log10(max_altitude) + log10(adult_weight)

Data: DF.rep_tree

AIC BIC logLik

-10.12714 -8.137668 10.06357

Correlation Structure: corPagel

Formula: ~1

Parameter estimate(s):

lambda

1.22097

Coefficients:

Value Std.Error t-value p-value

(Intercept) 0.9802289 0.09474795 10.345648 0.0000

log10(max_altitude) -0.3120446 0.04910086 -6.355176 0.0001

log10(adult_weight) 0.1704264 0.05168433 3.297449 0.0071

Correlation:

(Intr) lg10(m_)

log10(max_altitude) -0.078

log10(adult_weight) 0.079 -1.000

Standardized residuals:

Min Q1 Med Q3 Max

-1.06638287 -0.03818683 0.49015284 0.64924002 1.16056296

Residual standard error: 0.1591409

Degrees of freedom: 14 total; 11 residual

birth_weight

--------------------------------

Generalized least squares fit by REML

Model: log10(trait2) ~ log10(max_altitude) + log10(adult_weight)

Data: DF.rep_tree

AIC BIC logLik

0.3383049 0.7355127 4.830848

Correlation Structure: corPagel

Formula: ~1

Parameter estimate(s):

lambda

1.22097

Coefficients:

Value Std.Error t-value p-value

(Intercept) -0.23142310 0.13423332 -1.724036 0.1230

log10(max_altitude) 0.08384579 0.07308859 1.147180 0.2845

log10(adult_weight) 0.31096308 0.07692953 4.042181 0.0037

Correlation:

(Intr) lg10(m_)

log10(max_altitude) -0.078

log10(adult_weight) 0.079 -1.000

Standardized residuals:

Min Q1 Med Q3 Max

-1.248107130 0.003298405 0.154950604 0.894951064 1.858962786

Residual standard error: 0.2228056

Degrees of freedom: 11 total; 8 residual

clutch_size

--------------------------------

Generalized least squares fit by REML

Model: log10(trait2) ~ log10(max_altitude) + log10(adult_weight)

Data: DF.rep_tree

AIC BIC logLik

8.831961 10.34489 0.5840195

Correlation Structure: corPagel

Formula: ~1

Parameter estimate(s):

lambda

0.02168431

Coefficients:

Value Std.Error t-value p-value

(Intercept) -1.7452413 1.3905124 -1.2551066 0.2380

log10(max_altitude) 0.3349011 0.5171860 0.6475448 0.5319

log10(adult_weight) 0.4428724 0.1464004 3.0250756 0.0128

Correlation:

(Intr) lg10(m_)

log10(max_altitude) -0.967

log10(adult_weight) 0.394 -0.612

Standardized residuals:

Min Q1 Med Q3 Max

-1.6906197 -0.5497757 -0.2321904 0.6409786 1.4709055

Residual standard error: 0.2083616

Degrees of freedom: 13 total; 10 residual

clutches_pa

--------------------------------

Generalized least squares fit by REML

Model: log10(trait2) ~ log10(max_altitude) + log10(adult_weight)

Data: DF.rep_tree

AIC BIC logLik

8.831961 10.34489 0.5840195

Correlation Structure: corPagel

Formula: ~1

Parameter estimate(s):

lambda

0.02168431

Coefficients:

Value Std.Error t-value p-value

(Intercept) -1.7452413 1.3905124 -1.2551066 0.2380

log10(max_altitude) 0.3349011 0.5171860 0.6475448 0.5319

log10(adult_weight) 0.4428724 0.1464004 3.0250756 0.0128

Correlation:

(Intr) lg10(m_)

log10(max_altitude) -0.967

log10(adult_weight) 0.394 -0.612

Standardized residuals:

Min Q1 Med Q3 Max

-1.6906197 -0.5497757 -0.2321904 0.6409786 1.4709055

Residual standard error: 0.2083616

Degrees of freedom: 13 total; 10 residual

egg_weight

--------------------------------

Generalized least squares fit by REML

Model: log10(trait2) ~ log10(max_altitude) + log10(adult_weight)

Data: DF.rep_tree

AIC BIC logLik

2.526977 -7.473023 3.736511

Correlation Structure: corPagel

Formula: ~1

Parameter estimate(s):

lambda

1.000001

Coefficients:

Value Std.Error t-value p-value

(Intercept) -2.6769634 4.448112 -0.6018202 0.6551

log10(max_altitude) 0.9842384 2.332729 0.4219256 0.7458

log10(adult_weight) 0.2040011 1.460097 0.1397175 0.9116

Correlation:

(Intr) lg10(m_)

log10(max_altitude) -0.821

log10(adult_weight) 0.359 -0.827

Standardized residuals:

Testudo_hermanni Testudo_marginata Testudo_graeca Emys_orbicularis

0.5601265 0.6446207 0.5581288 -0.7404581

attr(,"std")

[1] 0.2508443 0.2508443 0.2508443 0.2508443

attr(,"label")

[1] "Standardized residuals"

Residual standard error: 0.2508443

Degrees of freedom: 4 total; 1 residual

female_maturity

--------------------------------

Generalized least squares fit by REML

Model: log10(trait2) ~ log10(max_altitude) + log10(adult_weight)

Data: DF.rep_tree

AIC BIC logLik

1.749325 -1.319203 4.125337

Correlation Structure: corPagel

Formula: ~1

Parameter estimate(s):

lambda

-0.8696305

Coefficients:

Value Std.Error t-value p-value

(Intercept) -1.8118949 1.6310574 -1.110871 0.3289

log10(max_altitude) 1.1236633 0.4576637 2.455216 0.0701

log10(adult_weight) 0.6156103 0.2629811 2.340892 0.0793

Correlation:

(Intr) lg10(m_)

log10(max_altitude) -0.861

log10(adult_weight) -0.491 -0.020

Standardized residuals:

Min Q1 Med Q3 Max

-2.1397076 -1.0823371 -0.2576484 0.2731824 1.4341895

Residual standard error: 0.09313289

Degrees of freedom: 7 total; 4 residual

incubation_time

--------------------------------

Generalized least squares fit by REML

Model: log10(trait2) ~ log10(max_altitude) + log10(adult_weight)

Data: DF.rep_tree

AIC BIC logLik

-2.295123 -0.3056462 6.147561

Correlation Structure: corPagel

Formula: ~1

Parameter estimate(s):

lambda

1.384862

Coefficients:

Value Std.Error t-value p-value

(Intercept) 1.8329594 0.7186798 2.550454 0.0270

log10(max_altitude) -0.0826069 0.2087082 -0.395801 0.6998

log10(adult_weight) 0.2081682 0.0299456 6.951547 0.0000

Correlation:

(Intr) lg10(m_)

log10(max_altitude) -0.995

log10(adult_weight) -0.669 0.686

Standardized residuals:

Min Q1 Med Q3 Max

-2.1763450 -1.5297806 -0.2041265 0.6151237 1.6590454

Residual standard error: 0.1596611

Degrees of freedom: 14 total; 11 residual

max_altitude

--------------------------------

Generalized least squares fit by REML

Model: log10(trait2) ~ log10(max_altitude) + log10(adult_weight)

Data: DF.rep_tree

AIC BIC logLik

-2.295123 -0.3056462 6.147561

Correlation Structure: corPagel

Formula: ~1

Parameter estimate(s):

lambda

1.384862

Coefficients:

Value Std.Error t-value p-value

(Intercept) 1.8329594 0.7186798 2.550454 0.0270

log10(max_altitude) -0.0826069 0.2087082 -0.395801 0.6998

log10(adult_weight) 0.2081682 0.0299456 6.951547 0.0000

Correlation:

(Intr) lg10(m_)

log10(max_altitude) -0.995

log10(adult_weight) -0.669 0.686

Standardized residuals:

Min Q1 Med Q3 Max

-2.1763450 -1.5297806 -0.2041265 0.6151237 1.6590454

Residual standard error: 0.1596611

Degrees of freedom: 14 total; 11 residual

max_longevity

--------------------------------

Generalized least squares fit by REML

Model: log10(trait2) ~ log10(max_altitude) + log10(adult_weight)

Data: DF.rep_tree

AIC BIC logLik

-2.295123 -0.3056462 6.147561

Correlation Structure: corPagel

Formula: ~1

Parameter estimate(s):

lambda

1.384862

Coefficients:

Value Std.Error t-value p-value

(Intercept) 1.8329594 0.7186798 2.550454 0.0270

log10(max_altitude) -0.0826069 0.2087082 -0.395801 0.6998

log10(adult_weight) 0.2081682 0.0299456 6.951547 0.0000

Correlation:

(Intr) lg10(m_)

log10(max_altitude) -0.995

log10(adult_weight) -0.669 0.686

Standardized residuals:

Min Q1 Med Q3 Max

-2.1763450 -1.5297806 -0.2041265 0.6151237 1.6590454

Residual standard error: 0.1596611

Degrees of freedom: 14 total; 11 residual

size_at_maturity

--------------------------------

Generalized least squares fit by REML

Model: log10(trait2) ~ log10(max_altitude) + log10(adult_weight)

Data: DF.rep_tree

AIC BIC logLik

0.5199617 -3.986977 4.740019

Correlation Structure: corPagel

Formula: ~1

Parameter estimate(s):

lambda

-1.05208

Coefficients:

Value Std.Error t-value p-value

(Intercept) 0.9126904 2.110118 0.4325304 0.6946

log10(max_altitude) -0.4807257 1.631236 -0.2947003 0.7874

log10(adult_weight) 0.5955353 1.382227 0.4308522 0.6957

Correlation:

(Intr) lg10(m_)

log10(max_altitude) -0.597

log10(adult_weight) 0.226 -0.916

Standardized residuals:

Min Q1 Med Q3 Max

-1.1074123 -0.9764005 -0.7428574 0.4868669 1.2813722

Residual standard error: 0.1074483

Degrees of freedom: 6 total; 3 residual

***************************************

Testudines-nonpgls

******************

birth_size_wt

--------------------------------

Generalized least squares fit by REML

Model: log10(trait2) ~ log10(max_altitude) + log10(adult_weight)

Data: DF.rep_tree

AIC BIC logLik

-8.447331 -6.85575 8.223666

Coefficients:

Value Std.Error t-value p-value

(Intercept) 0.9362329 0.6565918 1.425898 0.1817

log10(max_altitude) -0.2714394 0.2454923 -1.105694 0.2925

log10(adult_weight) 0.1584941 0.0727395 2.178927 0.0520

Correlation:

(Intr) lg10(m_)

log10(max_altitude) -0.963

log10(adult_weight) 0.366 -0.600

Standardized residuals:

Min Q1 Med Q3 Max

-2.0809663 -0.4962222 0.2673767 0.5802361 1.3695381

Residual standard error: 0.1041889

Degrees of freedom: 14 total; 11 residual

birth_weight

--------------------------------

Generalized least squares fit by REML

Model: log10(trait2) ~ log10(max_altitude) + log10(adult_weight)

Data: DF.rep_tree

AIC BIC logLik

6.902388 7.220154 0.5488059

Coefficients:

Value Std.Error t-value p-value

(Intercept) 1.6583432 1.8219561 0.9101993 0.3893

log10(max_altitude) -0.5975868 0.7322082 -0.8161433 0.4380

log10(adult_weight) 0.4220507 0.2082959 2.0262080 0.0773

Correlation:

(Intr) lg10(m_)

log10(max_altitude) -0.979

log10(adult_weight) 0.664 -0.802

Standardized residuals:

Min Q1 Med Q3 Max

-1.7196031 -0.3028120 -0.1393782 0.5259004 1.5465540

Residual standard error: 0.2123369

Degrees of freedom: 11 total; 8 residual

clutch_size

--------------------------------

Generalized least squares fit by REML

Model: log10(trait2) ~ log10(max_altitude) + log10(adult_weight)

Data: DF.rep_tree

AIC BIC logLik

6.832539 8.042879 0.5837307

Coefficients:

Value Std.Error t-value p-value

(Intercept) -1.7434242 1.3933983 -1.2512031 0.2393

log10(max_altitude) 0.3331978 0.5181415 0.6430634 0.5346

log10(adult_weight) 0.4432137 0.1467044 3.0211338 0.0129

Correlation:

(Intr) lg10(m_)

log10(max_altitude) -0.967

log10(adult_weight) 0.393 -0.611

Standardized residuals:

Min Q1 Med Q3 Max

-1.6832865 -0.5410977 -0.2207670 0.6562538 1.4883367

Residual standard error: 0.2075967

Degrees of freedom: 13 total; 10 residual

clutches_pa

--------------------------------

Generalized least squares fit by REML

Model: log10(trait2) ~ log10(max_altitude) + log10(adult_weight)

Data: DF.rep_tree

AIC BIC logLik

3.093423 3.882321 2.453289

Coefficients:

Value Std.Error t-value p-value

(Intercept) -2.5520270 1.2365392 -2.0638464 0.0690

log10(max_altitude) 0.8757636 0.4799655 1.8246388 0.1014

log10(adult_weight) 0.0466800 0.1357914 0.3437624 0.7389

Correlation:

(Intr) lg10(m_)

log10(max_altitude) -0.971

log10(adult_weight) 0.520 -0.706

Standardized residuals:

Min Q1 Med Q3 Max

-1.00442719 -0.69467505 -0.08353503 0.25164735 1.77371267

Residual standard error: 0.1692173

Degrees of freedom: 12 total; 9 residual

egg_weight

--------------------------------

Generalized least squares fit by REML

Model: log10(trait2) ~ log10(max_altitude) + log10(adult_weight)

Data: DF.rep_tree

AIC BIC logLik

0.5269775 -7.473023 3.736511

Coefficients:

Value Std.Error t-value p-value

(Intercept) -4.898174 9.823915 -0.4985970 0.7055

log10(max_altitude) 1.718899 4.581448 0.3751869 0.7715

log10(adult_weight) 0.189999 2.607082 0.0728781 0.9537

Correlation:

(Intr) lg10(m_)

log10(max_altitude) -0.848

log10(adult_weight) 0.309 -0.766

Standardized residuals:

140 316 317 320

-0.82908860 0.08562598 0.25149832 0.49196430

attr(,"std")

[1] 0.2808696 0.2808696 0.2808696 0.2808696

attr(,"label")

[1] "Standardized residuals"

Residual standard error: 0.2808696

Degrees of freedom: 4 total; 1 residual

female_maturity

--------------------------------

Generalized least squares fit by REML

Model: log10(trait2) ~ log10(max_altitude) + log10(adult_weight)

Data: DF.rep_tree

AIC BIC logLik

0.8352245 -1.619598 3.582388

Coefficients:

Value Std.Error t-value p-value

(Intercept) -1.3442136 1.8428381 -0.7294258 0.5062

log10(max_altitude) 1.0313727 0.5401624 1.9093751 0.1288

log10(adult_weight) 0.5464201 0.3159270 1.7295768 0.1588

Correlation:

(Intr) lg10(m_)

log10(max_altitude) -0.848

log10(adult_weight) -0.424 -0.119

Standardized residuals:

Min Q1 Med Q3 Max

-1.0611962 -0.6051608 0.1455837 0.4418535 1.2422272

Residual standard error: 0.1351918

Degrees of freedom: 7 total; 4 residual

incubation_time

--------------------------------

Generalized least squares fit by REML

Model: log10(trait2) ~ log10(max_altitude) + log10(adult_weight)

Data: DF.rep_tree

AIC BIC logLik

7.883775 9.475356 0.05811242

Coefficients:

Value Std.Error t-value p-value

(Intercept) 2.5231001 1.3793747 1.8291622 0.0946

log10(max_altitude) -0.3892448 0.5157327 -0.7547413 0.4663

log10(adult_weight) 0.2752600 0.1528119 1.8012991 0.0991

Correlation:

(Intr) lg10(m_)

log10(max_altitude) -0.963

log10(adult_weight) 0.366 -0.600

Standardized residuals:

Min Q1 Med Q3 Max

-1.2487269 -0.8127195 0.1068636 0.9132458 1.2111439

Residual standard error: 0.2188811

Degrees of freedom: 14 total; 11 residual

max_longevity

--------------------------------

Generalized least squares fit by REML

Model: log10(trait2) ~ log10(max_altitude) + log10(adult_weight)

Data: DF.rep_tree

AIC BIC logLik

2.631136 2.414776 2.684432

Coefficients:

Value Std.Error t-value p-value

(Intercept) 2.1119128 1.3038982 1.6196915 0.1493

log10(max_altitude) -0.2372841 0.4922031 -0.4820858 0.6445

log10(adult_weight) 0.0755120 0.1417123 0.5328540 0.6106

Correlation:

(Intr) lg10(m_)

log10(max_altitude) -0.969

log10(adult_weight) 0.471 -0.674

Standardized residuals:

Min Q1 Med Q3 Max

-0.9136856 -0.7918603 -0.2466987 0.7540675 1.2937445

Residual standard error: 0.1557798

Degrees of freedom: 10 total; 7 residual

size_at_maturity

--------------------------------

Generalized least squares fit by REML

Model: log10(trait2) ~ log10(max_altitude) + log10(adult_weight)

Data: DF.rep_tree

AIC BIC logLik

-0.7252631 -4.330814 4.362632

Coefficients:

Value Std.Error t-value p-value

(Intercept) 0.6780859 2.065727 0.3282553 0.7643

log10(max_altitude) -0.2581758 1.527402 -0.1690294 0.8765

log10(adult_weight) 0.4357851 1.279503 0.3405895 0.7559

Correlation:

(Intr) lg10(m_)

log10(max_altitude) -0.599

log10(adult_weight) 0.204 -0.906

Standardized residuals:

Min Q1 Med Q3 Max

-0.6165300 -0.4922536 -0.4337763 0.5478262 1.1208016

Residual standard error: 0.1435412

Degrees of freedom: 6 total; 3 residual

***************************************

Testudines-pgls

***************

birth_size_wt

--------------------------------

Generalized least squares fit by REML

Model: log10(trait2) ~ log10(max_altitude) + log10(adult_weight)

Data: DF.rep_tree

AIC BIC logLik

-10.12714 -8.137668 10.06357

Correlation Structure: corPagel

Formula: ~1

Parameter estimate(s):

lambda

1.22097

Coefficients:

Value Std.Error t-value p-value

(Intercept) 0.9802289 0.09474795 10.345648 0.0000

log10(max_altitude) -0.3120446 0.04910086 -6.355176 0.0001

log10(adult_weight) 0.1704264 0.05168433 3.297449 0.0071

Correlation:

(Intr) lg10(m_)

log10(max_altitude) -0.078

log10(adult_weight) 0.079 -1.000

Standardized residuals:

Min Q1 Med Q3 Max

-1.06638287 -0.03818683 0.49015284 0.64924002 1.16056296

Residual standard error: 0.1591409

Degrees of freedom: 14 total; 11 residual

birth_weight

--------------------------------

Generalized least squares fit by REML

Model: log10(trait2) ~ log10(max_altitude) + log10(adult_weight)

Data: DF.rep_tree

AIC BIC logLik

0.3383049 0.7355127 4.830848

Correlation Structure: corPagel

Formula: ~1

Parameter estimate(s):

lambda

1.22097

Coefficients:

Value Std.Error t-value p-value

(Intercept) -0.23142310 0.13423332 -1.724036 0.1230

log10(max_altitude) 0.08384579 0.07308859 1.147180 0.2845

log10(adult_weight) 0.31096308 0.07692953 4.042181 0.0037

Correlation:

(Intr) lg10(m_)

log10(max_altitude) -0.078

log10(adult_weight) 0.079 -1.000

Standardized residuals:

Min Q1 Med Q3 Max

-1.248107130 0.003298405 0.154950604 0.894951064 1.858962786

Residual standard error: 0.2228056

Degrees of freedom: 11 total; 8 residual

clutch_size

--------------------------------

Generalized least squares fit by REML

Model: log10(trait2) ~ log10(max_altitude) + log10(adult_weight)

Data: DF.rep_tree

AIC BIC logLik

8.831961 10.34489 0.5840195

Correlation Structure: corPagel

Formula: ~1

Parameter estimate(s):

lambda

0.02168431

Coefficients:

Value Std.Error t-value p-value

(Intercept) -1.7452413 1.3905124 -1.2551066 0.2380

log10(max_altitude) 0.3349011 0.5171860 0.6475448 0.5319

log10(adult_weight) 0.4428724 0.1464004 3.0250756 0.0128

Correlation:

(Intr) lg10(m_)

log10(max_altitude) -0.967

log10(adult_weight) 0.394 -0.612

Standardized residuals:

Min Q1 Med Q3 Max

-1.6906197 -0.5497757 -0.2321904 0.6409786 1.4709055

Residual standard error: 0.2083616

Degrees of freedom: 13 total; 10 residual

clutches_pa

--------------------------------

Generalized least squares fit by REML

Model: log10(trait2) ~ log10(max_altitude) + log10(adult_weight)

Data: DF.rep_tree

AIC BIC logLik

8.831961 10.34489 0.5840195

Correlation Structure: corPagel

Formula: ~1

Parameter estimate(s):

lambda

0.02168431

Coefficients:

Value Std.Error t-value p-value

(Intercept) -1.7452413 1.3905124 -1.2551066 0.2380

log10(max_altitude) 0.3349011 0.5171860 0.6475448 0.5319

log10(adult_weight) 0.4428724 0.1464004 3.0250756 0.0128

Correlation:

(Intr) lg10(m_)

log10(max_altitude) -0.967

log10(adult_weight) 0.394 -0.612

Standardized residuals:

Min Q1 Med Q3 Max

-1.6906197 -0.5497757 -0.2321904 0.6409786 1.4709055

Residual standard error: 0.2083616

Degrees of freedom: 13 total; 10 residual

egg_weight

--------------------------------

Generalized least squares fit by REML

Model: log10(trait2) ~ log10(max_altitude) + log10(adult_weight)

Data: DF.rep_tree

AIC BIC logLik

2.526977 -7.473023 3.736511

Correlation Structure: corPagel

Formula: ~1

Parameter estimate(s):

lambda

1.000001

Coefficients:

Value Std.Error t-value p-value

(Intercept) -2.6769634 4.448112 -0.6018202 0.6551

log10(max_altitude) 0.9842384 2.332729 0.4219256 0.7458

log10(adult_weight) 0.2040011 1.460097 0.1397175 0.9116

Correlation:

(Intr) lg10(m_)

log10(max_altitude) -0.821

log10(adult_weight) 0.359 -0.827

Standardized residuals:

Testudo_hermanni Testudo_marginata Testudo_graeca Emys_orbicularis

0.5601265 0.6446207 0.5581288 -0.7404581

attr(,"std")

[1] 0.2508443 0.2508443 0.2508443 0.2508443

attr(,"label")

[1] "Standardized residuals"

Residual standard error: 0.2508443

Degrees of freedom: 4 total; 1 residual

female_maturity

--------------------------------

Generalized least squares fit by REML

Model: log10(trait2) ~ log10(max_altitude) + log10(adult_weight)

Data: DF.rep_tree

AIC BIC logLik

1.749325 -1.319203 4.125337

Correlation Structure: corPagel

Formula: ~1

Parameter estimate(s):

lambda

-0.8696305

Coefficients:

Value Std.Error t-value p-value

(Intercept) -1.8118949 1.6310574 -1.110871 0.3289

log10(max_altitude) 1.1236633 0.4576637 2.455216 0.0701

log10(adult_weight) 0.6156103 0.2629811 2.340892 0.0793

Correlation:

(Intr) lg10(m_)

log10(max_altitude) -0.861

log10(adult_weight) -0.491 -0.020

Standardized residuals:

Min Q1 Med Q3 Max

-2.1397076 -1.0823371 -0.2576484 0.2731824 1.4341895

Residual standard error: 0.09313289

Degrees of freedom: 7 total; 4 residual

incubation_time

--------------------------------

Generalized least squares fit by REML

Model: log10(trait2) ~ log10(max_altitude) + log10(adult_weight)

Data: DF.rep_tree

AIC BIC logLik

-2.295123 -0.3056462 6.147561

Correlation Structure: corPagel

Formula: ~1

Parameter estimate(s):

lambda

1.384862

Coefficients:

Value Std.Error t-value p-value

(Intercept) 1.8329594 0.7186798 2.550454 0.0270

log10(max_altitude) -0.0826069 0.2087082 -0.395801 0.6998

log10(adult_weight) 0.2081682 0.0299456 6.951547 0.0000

Correlation:

(Intr) lg10(m_)

log10(max_altitude) -0.995

log10(adult_weight) -0.669 0.686

Standardized residuals:

Min Q1 Med Q3 Max

-2.1763450 -1.5297806 -0.2041265 0.6151237 1.6590454

Residual standard error: 0.1596611

Degrees of freedom: 14 total; 11 residual

max_altitude

--------------------------------

Generalized least squares fit by REML

Model: log10(trait2) ~ log10(max_altitude) + log10(adult_weight)

Data: DF.rep_tree

AIC BIC logLik

-2.295123 -0.3056462 6.147561

Correlation Structure: corPagel

Formula: ~1

Parameter estimate(s):

lambda

1.384862

Coefficients:

Value Std.Error t-value p-value

(Intercept) 1.8329594 0.7186798 2.550454 0.0270

log10(max_altitude) -0.0826069 0.2087082 -0.395801 0.6998

log10(adult_weight) 0.2081682 0.0299456 6.951547 0.0000

Correlation:

(Intr) lg10(m_)

log10(max_altitude) -0.995

log10(adult_weight) -0.669 0.686

Standardized residuals:

Min Q1 Med Q3 Max

-2.1763450 -1.5297806 -0.2041265 0.6151237 1.6590454

Residual standard error: 0.1596611

Degrees of freedom: 14 total; 11 residual

max_longevity

--------------------------------

Generalized least squares fit by REML

Model: log10(trait2) ~ log10(max_altitude) + log10(adult_weight)

Data: DF.rep_tree

AIC BIC logLik

-2.295123 -0.3056462 6.147561

Correlation Structure: corPagel

Formula: ~1

Parameter estimate(s):

lambda

1.384862

Coefficients:

Value Std.Error t-value p-value

(Intercept) 1.8329594 0.7186798 2.550454 0.0270

log10(max_altitude) -0.0826069 0.2087082 -0.395801 0.6998

log10(adult_weight) 0.2081682 0.0299456 6.951547 0.0000

Correlation:

(Intr) lg10(m_)

log10(max_altitude) -0.995

log10(adult_weight) -0.669 0.686

Standardized residuals:

Min Q1 Med Q3 Max

-2.1763450 -1.5297806 -0.2041265 0.6151237 1.6590454

Residual standard error: 0.1596611

Degrees of freedom: 14 total; 11 residual

size_at_maturity

--------------------------------

Generalized least squares fit by REML

Model: log10(trait2) ~ log10(max_altitude) + log10(adult_weight)

Data: DF.rep_tree

AIC BIC logLik

0.5199617 -3.986977 4.740019

Correlation Structure: corPagel

Formula: ~1

Parameter estimate(s):

lambda

-1.05208

Coefficients:

Value Std.Error t-value p-value

(Intercept) 0.9126904 2.110118 0.4325304 0.6946

log10(max_altitude) -0.4807257 1.631236 -0.2947003 0.7874

log10(adult_weight) 0.5955353 1.382227 0.4308522 0.6957

Correlation:

(Intr) lg10(m_)

log10(max_altitude) -0.597

log10(adult_weight) 0.226 -0.916

Standardized residuals:

Min Q1 Med Q3 Max

-1.1074123 -0.9764005 -0.7428574 0.4868669 1.2813722

Residual standard error: 0.1074483

Degrees of freedom: 6 total; 3 residual

***************************************

Reptilia-nonpgls

****************

birth_size_wt

--------------------------------

Generalized least squares fit by REML

Model: log10(trait2) ~ log10(max_altitude) + log10(adult_weight)

Data: DF.rep_tree

AIC BIC logLik

59.23372 68.2277 -25.61686

Coefficients:

Value Std.Error t-value p-value

(Intercept) -0.7069338 0.6663529 -1.060900 0.2924

log10(max_altitude) 0.4669438 0.2007782 2.325670 0.0229

log10(adult_weight) 0.0825266 0.0370114 2.229763 0.0290

Correlation:

(Intr) lg10(m_)

log10(max_altitude) -0.992

log10(adult_weight) -0.329 0.225

Standardized residuals:

Min Q1 Med Q3 Max

-2.4740825 -0.6180445 0.1271202 0.6862560 2.0582185

Residual standard error: 0.3256365

Degrees of freedom: 73 total; 70 residual

birth_weight

--------------------------------

Generalized least squares fit by REML

Model: log10(trait2) ~ log10(max_altitude) + log10(adult_weight)

Data: DF.rep_tree

AIC BIC logLik

73.3571 80.01135 -32.67855

Coefficients:

Value Std.Error t-value p-value

(Intercept) -1.0097447 0.9596595 -1.052191 0.2992

log10(max_altitude) 0.1816980 0.2865250 0.634144 0.5297

log10(adult_weight) 0.4898203 0.0866634 5.651984 0.0000

Correlation:

(Intr) lg10(m_)

log10(max_altitude) -0.978

log10(adult_weight) -0.307 0.116

Standardized residuals:

Min Q1 Med Q3 Max

-0.98981716 -0.54304329 -0.08769981 0.27603830 5.37710997

Residual standard error: 0.5022947

Degrees of freedom: 42 total; 39 residual

clutch_size

--------------------------------

Generalized least squares fit by REML

Model: log10(trait2) ~ log10(max_altitude) + log10(adult_weight)

Data: DF.rep_tree

AIC BIC logLik

45.72696 55.45023 -18.86348

Coefficients:

Value Std.Error t-value p-value

(Intercept) 0.05564304 0.3463344 0.160663 0.8727

log10(max_altitude) 0.11177770 0.1001868 1.115692 0.2677

log10(adult_weight) 0.21162955 0.0285447 7.413972 0.0000

Correlation:

(Intr) lg10(m_)

log10(max_altitude) -0.984

log10(adult_weight) -0.551 0.419

Standardized residuals:

Min Q1 Med Q3 Max

-2.8568591 -0.4725195 0.2072868 0.7256934 1.9241380

Residual standard error: 0.2831479

Degrees of freedom: 87 total; 84 residual

clutches_pa

--------------------------------

Generalized least squares fit by REML

Model: log10(trait2) ~ log10(max_altitude) + log10(adult_weight)

Data: DF.rep_tree

AIC BIC logLik

-5.860596 3.667511 6.930298

Coefficients:

Value Std.Error t-value p-value

(Intercept) -0.30262830 0.3293460 -0.9188765 0.3609

log10(max_altitude) 0.13206170 0.0998500 1.3226014 0.1897

log10(adult_weight) 0.00371329 0.0225769 0.1644728 0.8698

Correlation:

(Intr) lg10(m_)

log10(max_altitude) -0.990

log10(adult_weight) -0.269 0.148

Standardized residuals:

Min Q1 Med Q3 Max

-2.0179989 -0.6482666 -0.4341225 0.5016515 2.9213343

Residual standard error: 0.2079926

Degrees of freedom: 83 total; 80 residual

egg_weight

--------------------------------

Generalized least squares fit by REML

Model: log10(trait2) ~ log10(max_altitude) + log10(adult_weight)

Data: DF.rep_tree

AIC BIC logLik

12.09671 17.83266 -2.048357

Coefficients:

Value Std.Error t-value p-value

(Intercept) -1.5327250 0.3599744 -4.257872 0.0002

log10(max_altitude) 0.2417171 0.0960994 2.515283 0.0173

log10(adult_weight) 0.6372254 0.0377918 16.861466 0.0000

Correlation:

(Intr) lg10(m_)

log10(max_altitude) -0.965

log10(adult_weight) -0.720 0.538

Standardized residuals:

Min Q1 Med Q3 Max

-2.02419878 -0.57738517 0.03089747 0.53982171 2.65445372

Residual standard error: 0.2231941

Degrees of freedom: 34 total; 31 residual

female_maturity

--------------------------------

Generalized least squares fit by REML

Model: log10(trait2) ~ log10(max_altitude) + log10(adult_weight)

Data: DF.rep_tree

AIC BIC logLik

7.379626 15.10693 0.3101868

Coefficients:

Value Std.Error t-value p-value

(Intercept) 2.8847042 0.29991524 9.618398 0.0000

log10(max_altitude) -0.0763810 0.08470378 -0.901743 0.3714

log10(adult_weight) 0.2044657 0.02760407 7.407085 0.0000

Correlation:

(Intr) lg10(m_)

log10(max_altitude) -0.979

log10(adult_weight) -0.626 0.478

Standardized residuals:

Min Q1 Med Q3 Max

-1.6916509 -0.6923255 -0.1504677 0.6152160 2.1628754

Residual standard error: 0.2174823

Degrees of freedom: 54 total; 51 residual

incubation_time

--------------------------------

Generalized least squares fit by REML

Model: log10(trait2) ~ log10(max_altitude) + log10(adult_weight)

Data: DF.rep_tree

AIC BIC logLik

-3.181748 5.812233 5.590874

Coefficients:

Value Std.Error t-value p-value

(Intercept) 2.1455041 0.25872110 8.292729 0.0000

log10(max_altitude) -0.1089274 0.07415244 -1.468966 0.1463

log10(adult_weight) 0.0440721 0.02236385 1.970683 0.0527

Correlation:

(Intr) lg10(m_)

log10(max_altitude) -0.980

log10(adult_weight) -0.571 0.421

Standardized residuals:

Min Q1 Med Q3 Max

-2.73210912 -0.69239748 -0.03189333 0.43120358 3.04164270

Residual standard error: 0.2065441

Degrees of freedom: 73 total; 70 residual

max_longevity

--------------------------------

Generalized least squares fit by REML

Model: log10(trait2) ~ log10(max_altitude) + log10(adult_weight)

Data: DF.rep_tree

AIC BIC logLik

21.5193 29.08658 -6.759648

Coefficients:

Value Std.Error t-value p-value

(Intercept) 0.9275974 0.3376809 2.746965 0.0084

log10(max_altitude) -0.0309412 0.0957391 -0.323182 0.7479

log10(adult_weight) 0.1716295 0.0308964 5.554996 0.0000

Correlation:

(Intr) lg10(m_)

log10(max_altitude) -0.976

log10(adult_weight) -0.610 0.447

Standardized residuals:

Min Q1 Med Q3 Max

-2.09482909 -0.73922249 0.03850128 0.64144089 2.44331202

Residual standard error: 0.2501097

Degrees of freedom: 52 total; 49 residual

size_at_maturity

--------------------------------

Generalized least squares fit by REML

Model: log10(trait2) ~ log10(max_altitude) + log10(adult_weight)

Data: DF.rep_tree

AIC BIC logLik

36.11785 41.98079 -14.05892

Coefficients:

Value Std.Error t-value p-value

(Intercept) -0.3303348 0.5555710 -0.594586 0.5563

log10(max_altitude) 0.2568874 0.1493987 1.719476 0.0952

log10(adult_weight) 0.4090770 0.0557715 7.334872 0.0000

Correlation:

(Intr) lg10(m_)

log10(max_altitude) -0.971

log10(adult_weight) -0.682 0.508

Standardized residuals:

Min Q1 Med Q3 Max

-2.0336964 -0.6589384 0.1611054 0.8423616 1.5322691

Residual standard error: 0.326453

Degrees of freedom: 35 total; 32 residual

*****************************************

Reptilia-pgls

*************

birth_size_wt

--------------------------------

Generalized least squares fit by REML

Model: log10(trait2) ~ log10(max_altitude) + log10(adult_weight)

Data: DF.rep_tree

AIC BIC logLik

19.71245 30.88298 -4.856223

Correlation Structure: corPagel

Formula: ~1

Parameter estimate(s):

lambda

0.6099179

Coefficients:

Value Std.Error t-value p-value

(Intercept) 0.5487720 0.5403807 1.0155284 0.3134

log10(max_altitude) 0.0228887 0.1671683 0.1369198 0.8915

log10(adult_weight) 0.1327679 0.0427208 3.1078031 0.0027

Correlation:

(Intr) lg10(m_)

log10(max_altitude) -0.964

log10(adult_weight) -0.188 -0.001

Standardized residuals:

Min Q1 Med Q3 Max

-2.2427054 -0.3797175 0.4757077 1.0787719 2.8397548

Residual standard error: 0.301573

Degrees of freedom: 72 total; 69 residual

birth_weight

--------------------------------

Generalized least squares fit by REML

Model: log10(trait2) ~ log10(max_altitude) + log10(adult_weight)

Data: DF.rep_tree

AIC BIC logLik

73.83347 82.0214 -31.91674

Correlation Structure: corPagel

Formula: ~1

Parameter estimate(s):

lambda

-0.09586235

Coefficients:

Value Std.Error t-value p-value

(Intercept) -1.1541404 0.9326565 -1.237476 0.2235

log10(max_altitude) 0.2216470 0.2692614 0.823167 0.4155

log10(adult_weight) 0.5072727 0.0673478 7.532129 0.0000

Correlation:

(Intr) lg10(m_)

log10(max_altitude) -0.992

log10(adult_weight) -0.460 0.343

Standardized residuals:

Min Q1 Med Q3 Max

-1.0622670 -0.5926156 -0.1492117 0.2356790 5.4119741

Residual standard error: 0.4971359

Degrees of freedom: 41 total; 38 residual

clutch_size

--------------------------------

Generalized least squares fit by REML

Model: log10(trait2) ~ log10(max_altitude) + log10(adult_weight)

Data: DF.rep_tree

AIC BIC logLik

-10.12427 1.969932 10.06214

Correlation Structure: corPagel

Formula: ~1

Parameter estimate(s):

lambda

0.7640003

Coefficients:

Value Std.Error t-value p-value

(Intercept) -0.2528448 0.28414090 -0.889857 0.3761

log10(max_altitude) 0.1332946 0.08404635 1.585965 0.1165

log10(adult_weight) 0.2963569 0.03268710 9.066477 0.0000

Correlation:

(Intr) lg10(m_)

log10(max_altitude) -0.899

log10(adult_weight) -0.367 0.087

Standardized residuals:

Min Q1 Med Q3 Max

-2.9196363 -0.2353300 0.4214550 0.9690241 2.4683150

Residual standard error: 0.2729342

Degrees of freedom: 86 total; 83 residual

clutches_pa

--------------------------------

Generalized least squares fit by REML

Model: log10(trait2) ~ log10(max_altitude) + log10(adult_weight)

Data: DF.rep_tree

AIC BIC logLik

-40.26984 -28.4226 25.13492

Correlation Structure: corPagel

Formula: ~1

Parameter estimate(s):

lambda

0.7278892

Coefficients:

Value Std.Error t-value p-value

(Intercept) -0.8912407 0.29413831 -3.030006 0.0033

log10(max_altitude) 0.3134475 0.09147968 3.426416 0.0010

log10(adult_weight) 0.0207045 0.02708535 0.764417 0.4469

Correlation:

(Intr) lg10(m_)

log10(max_altitude) -0.939

log10(adult_weight) -0.213 -0.006

Standardized residuals:

Min Q1 Med Q3 Max

-1.8016104 -0.7569137 -0.3712034 0.5417121 2.4855263

Residual standard error: 0.2212014

Degrees of freedom: 82 total; 79 residual

egg_weight

--------------------------------

Generalized least squares fit by REML

Model: log10(trait2) ~ log10(max_altitude) + log10(adult_weight)

Data: DF.rep_tree

AIC BIC logLik

5.77232 12.94226 2.11384

Correlation Structure: corPagel

Formula: ~1

Parameter estimate(s):

lambda

0.6627736

Coefficients:

Value Std.Error t-value p-value

(Intercept) -0.8704276 0.3575523 -2.434407 0.0209

log10(max_altitude) 0.0718938 0.0927247 0.775347 0.4440

log10(adult_weight) 0.5687432 0.0514934 11.044964 0.0000

Correlation:

(Intr) lg10(m_)

log10(max_altitude) -0.883

log10(adult_weight) -0.642 0.286

Standardized residuals:

Min Q1 Med Q3 Max

-1.9757472 -0.5214570 0.1761839 0.7278376 2.5824963

Residual standard error: 0.239735

Degrees of freedom: 34 total; 31 residual

female_maturity

--------------------------------

Generalized least squares fit by REML

Model: log10(trait2) ~ log10(max_altitude) + log10(adult_weight)

Data: DF.rep_tree

AIC BIC logLik

-6.622186 2.937929 8.311093

Correlation Structure: corPagel

Formula: ~1

Parameter estimate(s):

lambda

0.6619513

Coefficients:

Value Std.Error t-value p-value

(Intercept) 3.1029093 0.28801701 10.773354 0.0000

log10(max_altitude) -0.0931805 0.08085102 -1.152496 0.2546

log10(adult_weight) 0.1559180 0.03621749 4.305047 0.0001

Correlation:

(Intr) lg10(m_)

log10(max_altitude) -0.913

log10(adult_weight) -0.533 0.251

Standardized residuals:

Min Q1 Med Q3 Max

-2.0023796 -0.9655616 -0.2786377 0.2903656 1.9968126

Residual standard error: 0.2271471

Degrees of freedom: 53 total; 50 residual

incubation_time

--------------------------------

Generalized least squares fit by REML

Model: log10(trait2) ~ log10(max_altitude) + log10(adult_weight)

Data: DF.rep_tree

AIC BIC logLik

-22.99255 -11.82202 16.49628

Correlation Structure: corPagel

Formula: ~1

Parameter estimate(s):

lambda

0.7871671

Coefficients:

Value Std.Error t-value p-value

(Intercept) 2.0537142 0.25757075 7.973398 0.0000

log10(max_altitude) -0.0775378 0.07483408 -1.036130 0.3038

log10(adult_weight) 0.0547150 0.03200928 1.709349 0.0919

Correlation:

(Intr) lg10(m_)

log10(max_altitude) -0.889

log10(adult_weight) -0.400 0.088

Standardized residuals:

Min Q1 Med Q3 Max

-2.4367093 -0.7194990 -0.1466225 0.2634192 2.3411458

Residual standard error: 0.2416462

Degrees of freedom: 72 total; 69 residual

max_altitude

--------------------------------

Generalized least squares fit by REML

Model: log10(trait2) ~ log10(max_altitude) + log10(adult_weight)

Data: DF.rep_tree

AIC BIC logLik

-22.99255 -11.82202 16.49628

Correlation Structure: corPagel

Formula: ~1

Parameter estimate(s):

lambda

0.7871671

Coefficients:

Value Std.Error t-value p-value

(Intercept) 2.0537142 0.25757075 7.973398 0.0000

log10(max_altitude) -0.0775378 0.07483408 -1.036130 0.3038

log10(adult_weight) 0.0547150 0.03200928 1.709349 0.0919

Correlation:

(Intr) lg10(m_)

log10(max_altitude) -0.889

log10(adult_weight) -0.400 0.088

Standardized residuals:

Min Q1 Med Q3 Max

-2.4367093 -0.7194990 -0.1466225 0.2634192 2.3411458

Residual standard error: 0.2416462

Degrees of freedom: 72 total; 69 residual

max_longevity

--------------------------------

Generalized least squares fit by REML

Model: log10(trait2) ~ log10(max_altitude) + log10(adult_weight)

Data: DF.rep_tree

AIC BIC logLik

11.671 21.1301 -0.8355004

Correlation Structure: corPagel

Formula: ~1

Parameter estimate(s):

lambda

0.7112462

Coefficients:

Value Std.Error t-value p-value

(Intercept) 1.1356941 0.3305555 3.435714 0.0012

log10(max_altitude) -0.0741088 0.0954457 -0.776450 0.4412

log10(adult_weight) 0.1497994 0.0411277 3.642294 0.0007

Correlation:

(Intr) lg10(m_)

log10(max_altitude) -0.903

log10(adult_weight) -0.454 0.149

Standardized residuals:

Min Q1 Med Q3 Max

-2.02845155 -0.68216960 -0.05729996 0.51683561 1.97124988

Residual standard error: 0.2775965

Degrees of freedom: 52 total; 49 residual

size_at_maturity

--------------------------------

Generalized least squares fit by REML

Model: log10(trait2) ~ log10(max_altitude) + log10(adult_weight)

Data: DF.rep_tree

AIC BIC logLik

0.2454038 7.574083 4.877298

Correlation Structure: corPagel

Formula: ~1

Parameter estimate(s):

lambda

0.8980907

Coefficients:

Value Std.Error t-value p-value

(Intercept) 0.5745849 0.3613803 1.589973 0.1217

log10(max_altitude) -0.0053389 0.0943650 -0.056577 0.9552

log10(adult_weight) 0.3364971 0.0550856 6.108616 0.0000

Correlation:

(Intr) lg10(m_)

log10(max_altitude) -0.861

log10(adult_weight) -0.582 0.204

Standardized residuals:

Min Q1 Med Q3 Max

-1.780863 -0.753369 0.656559 1.515539 2.488207

Residual standard error: 0.2713523

Degrees of freedom: 35 total; 32 residual
